# Chemical Aspects of Human and Environmental Overload with Fluorine

**DOI:** 10.1021/acs.chemrev.0c01263

**Published:** 2021-03-16

**Authors:** Jianlin Han, Loránd Kiss, Haibo Mei, Attila Márió Remete, Maja Ponikvar-Svet, Daniel Mark Sedgwick, Raquel Roman, Santos Fustero, Hiroki Moriwaki, Vadim A. Soloshonok

**Affiliations:** † Jiangsu Co-Innovation Center of Efficient Processing and Utilization of Forest Resources, College of Chemical Engineering, Nanjing Forestry University, Nanjing 210037, China; ‡ University of Szeged, Institute of Pharmaceutical Chemistry and Interdisciplinary Excellence Centre, Eötvös u. 6, 6720 Szeged, Hungary; § Department of Inorganic Chemistry and Technology, Jožef Stefan Institute, Jamova cesta 39, 1000 Ljubljana, Slovenia; ∥ Departamento de Química Orgánica, Universidad de Valencia, 46100 Burjassot, Valencia Spain; ⊥ Hamari Chemicals Ltd., 1-19-40, Nankokita, Suminoe-ku, Osaka 559-0034, Japan; ¶ Department of Organic Chemistry I, Faculty of Chemistry, University of the Basque Country UPV/EHU, 20018 San Sebastian, Spain; △ IKERBASQUE, Basque Foundation for Science, 48011 Bilbao, Spain

## Abstract

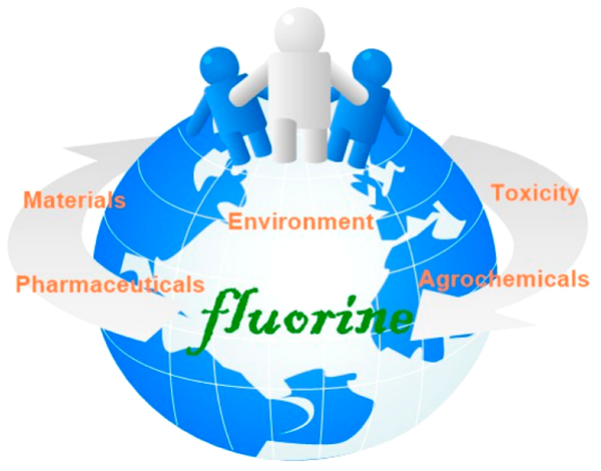

Over the last 100–120 years, due to the ever-increasing importance of fluorine-containing compounds in modern technology and daily life, the explosive development of the fluorochemical industry led to an enormous increase of emission of fluoride ions into the biosphere. This made it more and more important to understand the biological activities, metabolism, degradation, and possible environmental hazards of such substances. This comprehensive and critical review focuses on the effects of fluoride ions and organofluorine compounds (mainly pharmaceuticals and agrochemicals) on human health and the environment. To give a better overview, various connected topics are also discussed: reasons and trends of the advance of fluorine-containing pharmaceuticals and agrochemicals, metabolism of fluorinated drugs, withdrawn fluorinated drugs, natural sources of organic and inorganic fluorine compounds in the environment (including the biosphere), sources of fluoride intake, and finally biomarkers of fluoride exposure.

## 1 Introduction

The aim of the current review is to give a comprehensive, authoritative, critical, and highly appealing account of general interest to the chemistry community. Namely, it deals with the emerging issues of a high socioeconomic impact–the rapidly growing number of fluorine-containing pharmaceuticals and agrochemicals and their effects on human health and the environment.

The fluorochemical industry has greatly expanded in the last 100–150 years because numerous fluorinated products have shown their decisive importance in many areas linked to our daily life.^1−4^ Hydrogen fluoride, the key intermediate, is prepared from fluorospar (CaF_2_) via treatment with sulfuric acid, and it is mainly used to synthesize cryolite (for aluminum production) and fluorocarbons (mainly used as refrigerants). Within fluorocarbons, initially, production of chlorofluorocarbons (CFCs) was the most prominent. However, the discovery of the detrimental effect of CFCs on the ozone layer led to their phase out (Montreal Protocol, 1987), and they were replaced with hydrochlorofluorocarbons (HCFCs) and hydrofluorocarbons (HFCs). The former are mainly temporary CFC replacements, while the latter are seen as long-term alternatives (although their greenhouse effects also raised concerns).^1^ The synthesis of fluorinating reagents and fluorinated building blocks for organic synthesis as well as the production of elemental fluorine, fluoropolymers (such as Teflon), and perfluorinated compounds are minor but important applications of HF.^1−5^ Furthermore, elemental fluorine is essential for the nuclear industry (enrichment of ^235^U is achieved by gas centrifugation of uranium hexafluoride, which is obtained via treatment of UF_4_ with F_2_ gas).^6,7^ Based on their exceptional optical properties, some inorganic fluorides have small scale but important applications (such as optics for deep ultraviolet photolithography used in the manufacture of microelectronics).^5^

Fluorine-containing organic compounds not only are useful in scientific research^8−11^ but also have high practical importance. Hydrofluorocarbon refrigerants were already mentioned above. Fluorinated liquid crystal monomers are widely used in LCD devices.^3^ Fluoropolymers have many attractive properties (chemical resistance, thermal and weather stability, flame resistance, good mechanical properties, and high dielectric breakdown voltage) which resulted in their widespread use (e.g., in the electrical, chemical, automotive, and medicinal industry).^4^ Fluorine also plays a critical role in the development of modern pharmaceuticals because introduction of fluorine often enhances the bioactivity and metabolic stability.^12−14^ Currently, fluorine-containing compounds constitute around 25% of small-molecule drugs in the clinic, and 25–30% of newly introduced drugs contain fluorine atom(s).^13−15^ Among the best selling and most prescribed drugs, fluorinated preparations are even more prevalent.^16,17^ The current trend suggests that in the next 10–15 years fluorinated drugs will dominate the pharmaceutical market. An even more profound fluorine trend exists in the agricultural industry since upon “fluorination” different physicochemical properties of active ingredients like lipophilicity, water solubility, and metabolic stability could be fine-tuned.^18−20^ Fluorination of drugs and agrochemicals is well-rationalized, and most definitely, it is very beneficial, providing more potent life-saving medicines and selective crop-protection agents.^12−14,18^ This trend is very welcomed and of critical importance for our aging and overpopulated modern western society. Synthesis of ^18^F-containing radiopharmaceuticals is also an emerging area.^21,22^

However, our meticulous review of the relevant literature has pointed to an inherent potential problem. Although fluorine is the 24th most abundant element in the universe and the 13th most common element in the earth’s crust (0.059% by weight),^23^ it is virtually absent from the biosphere.^24,25^ The underlying obvious reasons are as follows: (i) the minerals fluorspar (CaF_2_), fluorapatite [Ca_5_(PO_4_)_3_F], and cryolite (Na_3_AlF_6_), the three richest natural sources of fluorine, are practically insoluble in water and, therefore, are out of the biological realm;^26^ (ii) the high oxidation potential of fluorine (−3.06 V, much higher than that of the rest of halogens) makes it impossible to form the fluorine analogues of hypohalites or other electrophilic halogen species involved in most of the known enzymatic halogenation processes; and (iii) finally, the high hydration energy of the fluoride ion (490 kJ mol^–1^) renders it a very poor nucleophile in the aqueous/biological environment, and therefore, it is unsuitable to form organic C–F bonds via typical nucleophilic substitutions.^24,25^

Thus, fluoride is xenobiotic, and the effects of fluoride and fluoroorganic compounds on the biosphere and on processes essential to human life and health were not thoroughly studied in the past. However, as a consequence of the growth of the fluorochemical industry and the increasing role of fluorine in the development of new pharmaceuticals and agrochemicals, the emission of fluoride and organofluorine compounds greatly increased, and efforts to understand the physiological effects of these substances have been renewed.

Approximately half of the fluoride intake is quickly deposited in calcified tissues like bones and teeth. Its release is a much slower process; that is, fluoride can accumulate in the body.^27^ It has long been known that excess fluoride intake can result in dental fluorosis in children or skeletal fluorosis in children and adults alike.^27^ More recent studies uncovered that fluoride can cause numerous other health problems, such as impaired thyroid and endocrine system function or developmental neurotoxicity.^28^ It is quite unnerving that aluminum(III) fluorocomplexes can activate G-protein-coupled receptors at much lower concentrations than either Al^3+^ or F^–^ acting alone.^29^ Important sources of fluoride pollution are the aluminum industry and phosphate fertilizers.^1,30^ The latter often have considerable fluoride content (1–3% in the case of superphosphate), which is released directly into the soil. Although fluoride is strongly bound in soils, it can still endanger grazing livestock.^30^ Community water fluoridation, which has a high influence on fluoride intake, is one of the most controversial topics in medicine.^28^

The increased usage of polyfluoroalkyl substances, fluorine-containing drugs, and fluorinated agrochemicals generated new dangers. On one hand, metabolism of fluorinated compounds may produce toxic fluorine-containing metabolites (e.g., fluoride or fluoroacetate), leading to increased concentrations of fluoride in body fluids and tissues. It is a major concern, in particular, because fluorinated drugs are in the most prescribed medications, and an average patient may take several fluorine-containing drugs at the same time. There are documented cases of fluorinated drugs whose side effects are at least partially based on such toxic fluoro-metabolites (for example, α-fluoro-β-alanine and fluoroacetate in the case of the anticancer drug 5-fluorouracil).^31^ Sometimes, these problems resulted in withdrawal or restriction (a good example is the heavily fluorinated anesthetic agent methoxyflurane).^32^ On the other hand, many fluorinated entities, especially perfluorinated ones, are highly resistant to degradation, leading to their accumulation in the environment.^33^ Currently, residues of various fluorinated organic compounds (polyfluoroalkyl substances, drugs, and agrochemicals) can be detected in surface waters and biological samples.^33−36^ The history of various polychlorinated compounds (DDT, γ-hexachlorocyclohexane, polychlorinated biphenyls, etc.) clearly illustrates the dangers of such persistent organic pollutants.^37^

Until now, environmental and healthcare policies concerning fluorine-containing compounds mainly focused on limiting emissions from the aluminum industry, the ban of ozone-depleting CFCs, and keeping the fluoride content in drinking water below the accepted thresholds.^1,27^ However, with the emergence of organofluorine compounds, efforts should be taken to carefully study the metabolism of various fluorine-containing groups and substances in order to identify derivatives of better biological tolerance (posing no risk to human health and the environment) and those which are problematic. The first steps are already in progress (perfluorooctanesulfonic acid and perfluorooctanoic acid were phased out because of their harmful effects), but there is much work to be done.^33^

The goal of this review is to raise these issues and provide a comprehensive discussion of all relevant data available in the literature. Section 2 covers the occurrence and available biosynthetic data of fluorine-containing natural products. Section 3 describes the beneficial effects of fluorine incorporation into drugs and agrochemicals in detail, and it includes a statistical analysis of recent fluorination trends of these compound families. Section 4 focuses on environmental sources of fluorine-containing compounds and their daily intake. Section 5 reviews available data on the metabolism of organofluorine compounds and environmental concerns related to these substances. In Section 6, dangers of fluorine-containing compounds are illustrated using withdrawn fluorinated drugs as examples. Section 7 summarizes the reasons behind the poisonous nature of fluoride and the most common consequences of acute and chronic fluoride toxicity. Finally, Section 8 discusses biomarkers of recent and historic fluoride exposure.

## 2 Fluorine in Biosphere

### 2.1 General Properties of Fluorine and Its Xenobiotic Nature

Fluorine, the 13th most common element in the earth’s crust (0.059% by weight), is the most abundant halogen on Earth. Chlorine is the 19th; bromine is the 49th; and iodine is the 62th on the list.^23^ Because of its extreme electronegativity, fluorine is highly reactive and occurs in nature almost exclusively as fluoride salts.^38,39^

Despite its abundance, fluorine is rarely incorporated into natural products. Among the more than 5000 known naturally produced halogen-substituted compounds, only a handful contain fluorine.^24,25^ Various reasons are responsible for this phenomenon. For example, the amount of bioavailable fluoride is greatly limited (e.g., seawater contains only 1.3 mg L^–1^ of F^–^ in contrast to 19 g L^–1^ of Cl^–^) because the most common fluoride sources, fluorite (CaF_2_), fluoroapatite [Ca_5_(PO_4_)_3_F], and cryolite (Na_3_AlF_6_), are insoluble in water.^26^ It is also important to note that the high electronegativity of fluorine prohibits oxidation of fluoride ions under biological conditions, excluding radical or electrophilic fluorination pathways. The only remaining possibility, nucleophilic fluorination, is hindered by the very strong hydration of F^–^, which greatly reduces its nucleophilicity.^24,25^

Despite the above difficulties, some organisms still found ways to produce fluorinated molecules. This section will discuss these compounds, their biological activity, and information available about their biosynthesis.

### 2.2 Fluoroacetate

**Scheme 1:**
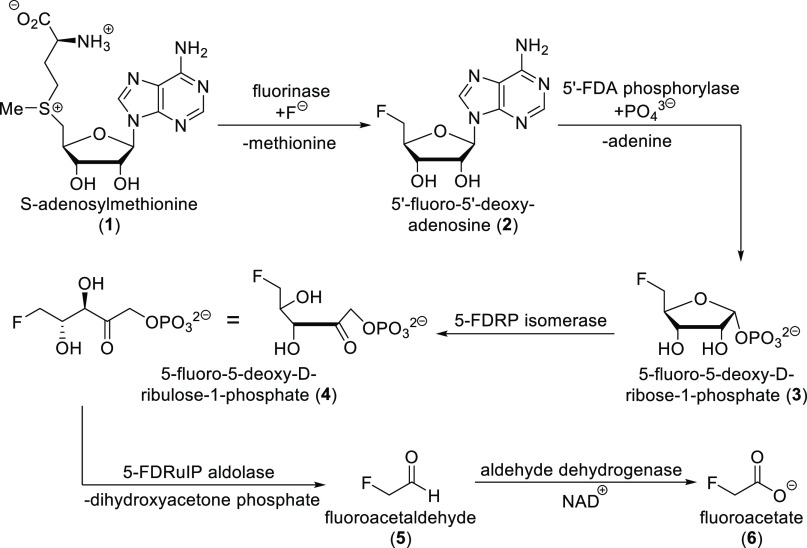
Biosynthesis of Fluoroacetate in *Streptomyces cattleya*

Fluoroacetate (**6**, Scheme 1) was first isolated in 1943 from *Dichapetalum cymosum*, a poisonous plant found in South Africa. Its leaves contain up to 250 mg kg^–1^ of fluoroacetate, which is the main component responsible for its toxicity. Since that time, more than 40 plant species were found with high fluoroacetate content. These include numerous *Dichapetalum* species, several *Amorima* species, *Palicourea marcgravii* and *P. aeneofusca*, *Gastrolobium grandiflorum* and *G. parviflorum*, *Arrabidaea bilabiata*, *Spondianthus preussii*, and *Cyamopsis tetragonolobus*. The record is held by *D. braunii* seeds (8000 mg kg^–1^ of fluoroacetate). These plants grow in tropical and semitropical areas of Africa, Australia, and South America. Their fluoroacetate accumulation is possibly related to a defense strategy against herbivores. Interestingly, nontoxic levels (trace amounts) of fluoroacetate (and the related (2*R*,3*R*)-fluorocitrate, see below) can be found in some edible plants too (for example, tea leaves, soya bean, oatmeal). This suggests that the ability to produce fluoroacetate is widespread among plants, and a limited number of species were capable of greatly amplifying this ability. It is worth mentioning that the mechanism of fluoroacetate production in plants is still unknown.^26,40^

**Scheme 2:**
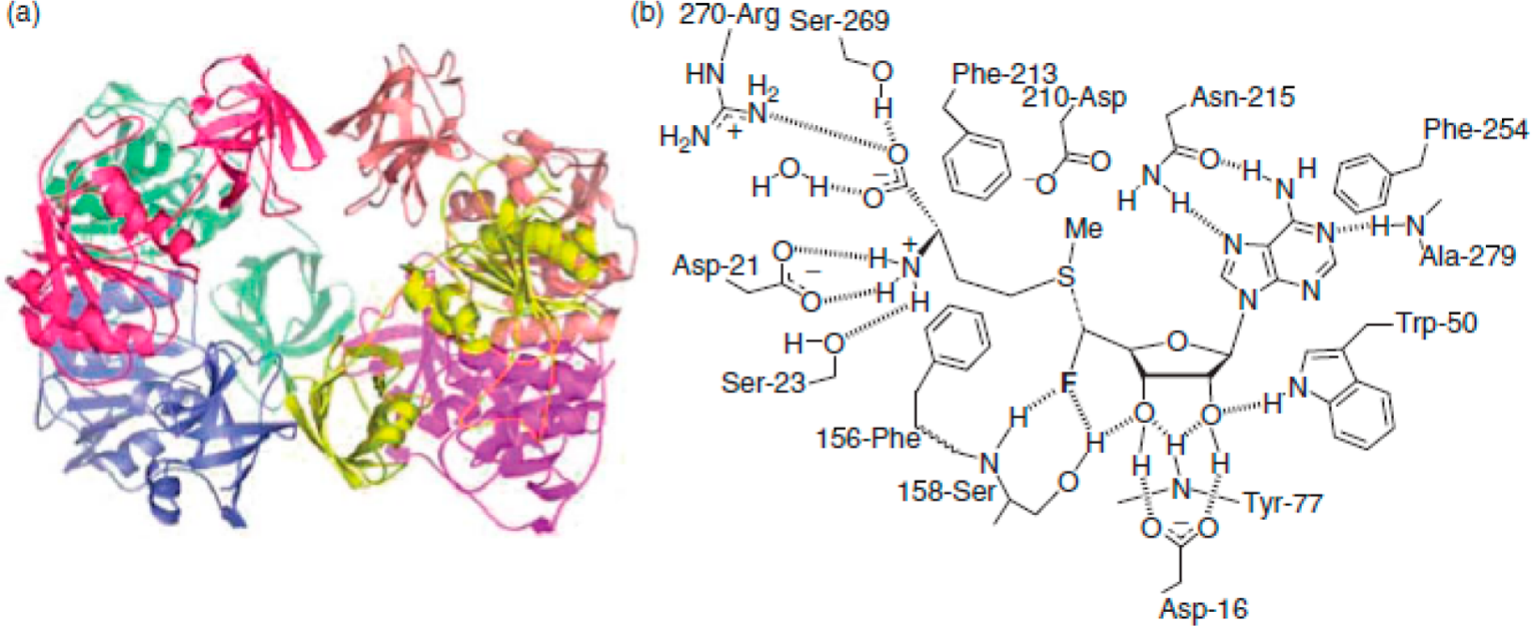
Structure of the Fluorinase Enzyme (Left) and Its Active Site (Right)

Furthermore, some microorganisms also produce fluoroacetate. An important example is *Streptomyces cattleya*. Its ability to produce fluoroacetate and (2*S*,3*S*)-4-fluorothreonine was discovered in 1986. The whole biosynthetic pathway was uncovered, supported by full genomic sequenation. The first step in the fluorometabolite synthesis by *S. cattleya* is the reaction of the F^–^ ion with *S*-adenosylmethionine **1** to produce 5′-fluoro-5′-deoxyadenosine **2**.^24,25^ This reaction is catalyzed by the fluorinase enzyme, which was isolated, and its crystal structure was determined. Three monomeric units form a trimer, and two trimers then form the final hexameric structure (Scheme 2). The monomeric unit represented a new protein fold different from any previously solved analogues. Fluorinase increases the nucleophilicity of fluoride by its desolvation (hydrogen bond donor groups of the enzyme replace water molecules around fluoride; see Scheme 2).^41^ Isotopic labeling confirmed that the catalyzed nucleophilic fluorination reaction follows an S_N_2 mechanism with configurational inversion.^42^ 5′-Fluoro-5′-deoxyadenosine **2** is then converted to 5-fluoro-5-deoxy-d-ribose-1-phosphate **3** by a purine nucleoside phosphorylase. Subsequent enzymatic isomerization produces (3*R*,4*S*)-5-fluoro-5-deoxy-d-ribulose-1-phosphate **4**, which is then cleaved by an aldolase to produce fluoroacetaldehyde **5** and followed by oxidation with a NAD^+^-dependent aldehyde dehydrogenase to fluoroacetate **6** (Scheme 1). Later, genetic sequencing utilizing *S. cattleya* fluorinase enabled the discovery of five similar fluorinases via genome mining.^24,25,43^

**Scheme 3:**
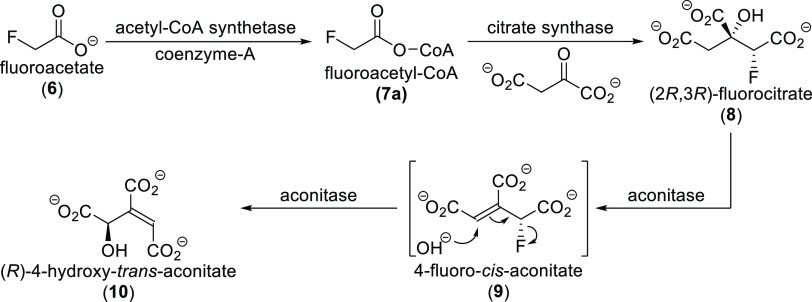
Transformation of Fluoroacetate in the Citrate Cycle

The high toxicity of fluoroacetate is caused by its metabolism (Scheme 3). Fluoroacetate is transformed into fluoroacetyl-CoA **7**, which reacts with oxaloacetate in the presence of citrate synthase to form (2*R*,3*R*)-fluorocitrate **8**, the only toxic 2-fluorocitrate stereoisomer. This compound inhibits citrate transport. In addition, the aconitase enzyme transforms (2*R*,3*R*)-fluorocitrate **8** into (*R*)-4-hydroxy-*trans*-aconitate **10**, which then inhibits aconitase. Through these actions, fluoroacetate and (2*R*,3*R*)-fluorocitrate block the citric acid cycle, impairing oxidative metabolism and causing citrate accumulation in several tissues. The reduction of ionized serum calcium concentrations also contributes to toxicity.^24,25,40,44^

Organisms producing fluoroacetate have to defend themselves against self-poisoning. *D. cymosum* achieves this by a fluoroacetyl-CoA hydrolase. Of acetyl-CoA and fluoroacetyl-CoA, this enzyme selectively hydrolyzes the latter one, preventing (2*R*,3*R*)-fluorocitrate formation. It is also worthwhile to mention that plants producing fluoroacetate seem to be capable of catabolizing fluoroacetate. *S. cattleya* also has a fluoroacetyl-CoA hydrolase, but this microorganism has an additional defense mechanism: it only starts synthesizing fluorinated metabolites after it stopped its growth, that is, when the citrate cycle is inactive.^24,25^

Herbivores, living in areas where plants synthesizing fluoroacetate occur, also developed increased resistance to fluoroacetate. It seems that they have a glutathione-dependent fluoroacetate-specific defluorinase enzyme (mostly in their livers), which converts fluoroacetate into *S*-carboxymethylcysteine and a fluoride ion. Various soil microbes are also capable of decomposing fluoroacetate into glycolate and F^–^ utilizing a fluoroacetate dehalogenase enzyme.^24,25^

### 2.3 (2*R*,3*R*)-Fluorocitrate

(2*R*,3*R*)-Fluorocitrate (**8**, Scheme 3) is formed when fluoroacetate enters the citric acid cycle. Since a large number of plants are capable of synthesizing low amounts of fluoroacetate, they also contain traces of its metabolite (2*R*,3*R*)-fluorocitrate (e.g., commercial tea can contain up to 30 mg kg^–1^ on a dry weight basis). As explained in Section 2.2, (2*R*,3*R*)-fluorocitrate is the only toxic stereoisomer among 2-fluorocitrates, and it exerts its toxicity via a number of effects (inhibition of citrate transport, reduction of ionized serum calcium concentration, and inhibition of the citric acid cycle via its metabolite (*R*)-4-hydroxy-*trans*-aconitate).^40^

### 2.4 ω-Fluorinated Fatty Acids

**Scheme 4:**
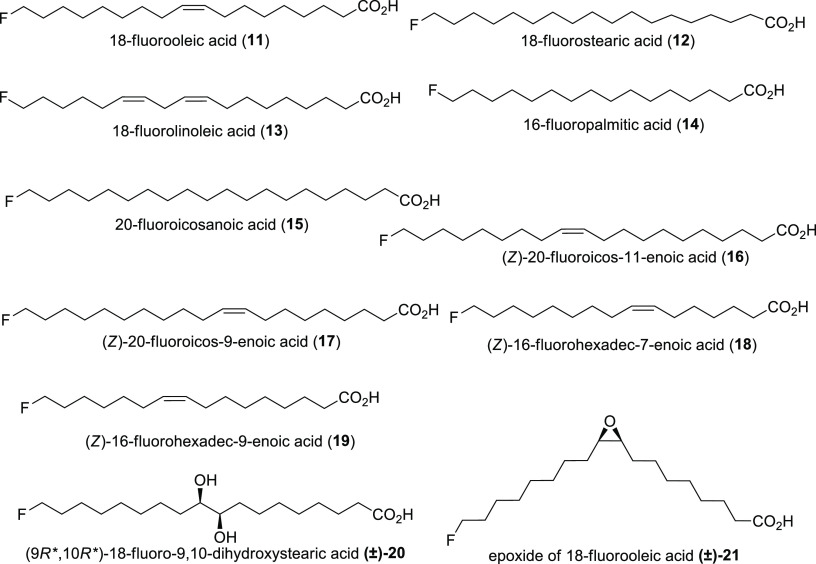
Naturally Produced ω-Fluorinated Fatty Acids

ω-Fluorinated fatty acids were discovered in the seed oil of the West African plant *Dichapetalum toxicarium*, and their production seems to be restricted to this single species.^24,25,40^ According to a GC/MS study, 12.9% of the total fatty acid content in the seed oil is comprised of ω-fluorinated compounds. The same study showed that 74% of the ω-fluorinated fatty acids is 18-fluorooleic acid **11**; 16% is 18-fluorostearic acid **12**; 6% is 18-fluorolinoleic acid **13**; and 4% is 16-fluoropalmitic acid **14**. Other ω-fluorinated fatty acids (20-fluoroicosanoic acid **15**, (*Z*)-20-fluoroicos-11-enoic acid **16**, (*Z*)-20-fluoroicos-9-enoic acid **17**, (*Z*)-16-fluorohexadec-7-enoic acid **18**, and (*Z*)-16-fluorohexadec-9-enoic acid **19**) were detected in lower amounts.^45^ Other studies found *threo*-18-fluoro-9,10-dihydroxystearic acid **20** and evidence for the presence of 18-fluoro-9,10-epoxyoleic acid **(±)-21**.^46,47^Scheme 4 summarizes these compounds. In 1964, ω-fluorocapric acid and ω-fluoromyristic acid were tentatively identified in the oil on the basis of GC retention time; however, newer studies did not confirm this information.^46^

These compounds, all of them with an even number of carbon atoms, are very toxic because their *in vitro* metabolism leads to fluoroacetate. In stark contrast with fluoroacetate, they are lipophilic and can also be toxic by direct absorption through the skin.^24,25,40^

**Scheme 5:**
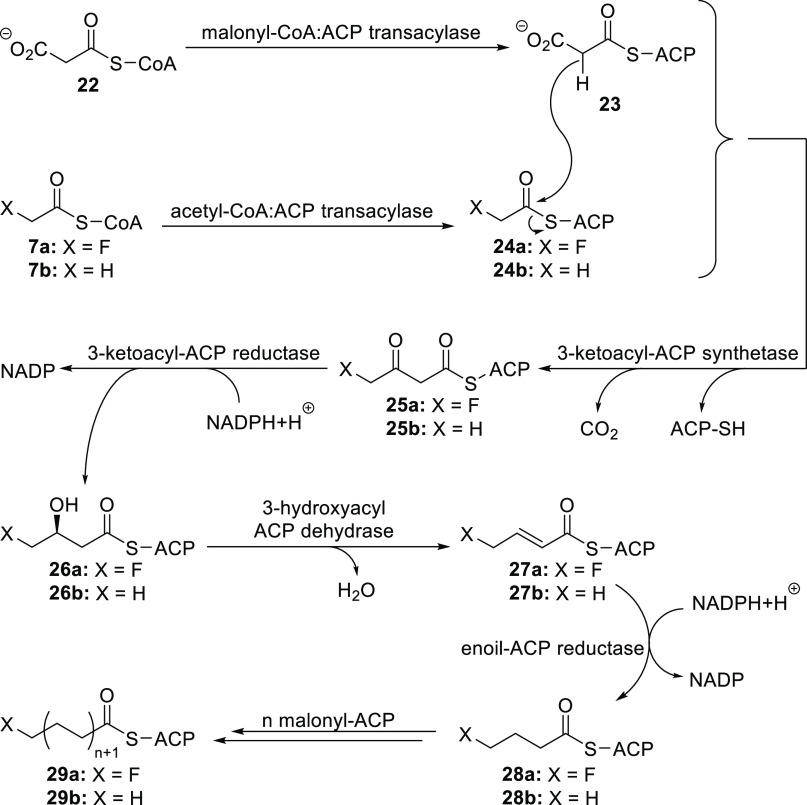
Plausible Biosynthetic Pathway toward ω-Fluorinated Fatty Acids (ACP = Acyl Carrier Protein)

Ratios of the ω-fluorinated fatty acids are similar to those of their nonfluorinated counterparts (which are present in 5 to 10 times higher amounts).^24,25,40,46^ This suggests a common origin. The most plausible explanation is that in this plant the first step of fatty acid synthesis, namely, condensation of acetyl-CoA with a malonyl acyl carrier protein or malonyl-ACP, works with fluoroacetyl-CoA too. In contrast, fluoromalonyl-ACP either is not formed or cannot be incorporated to fatty acids in the same manner as malonyl-ACP (Scheme 5).^24,25,40^

### 2.5 (2*S*,3*S*)-4-Fluorothreonine

**Scheme 6:**
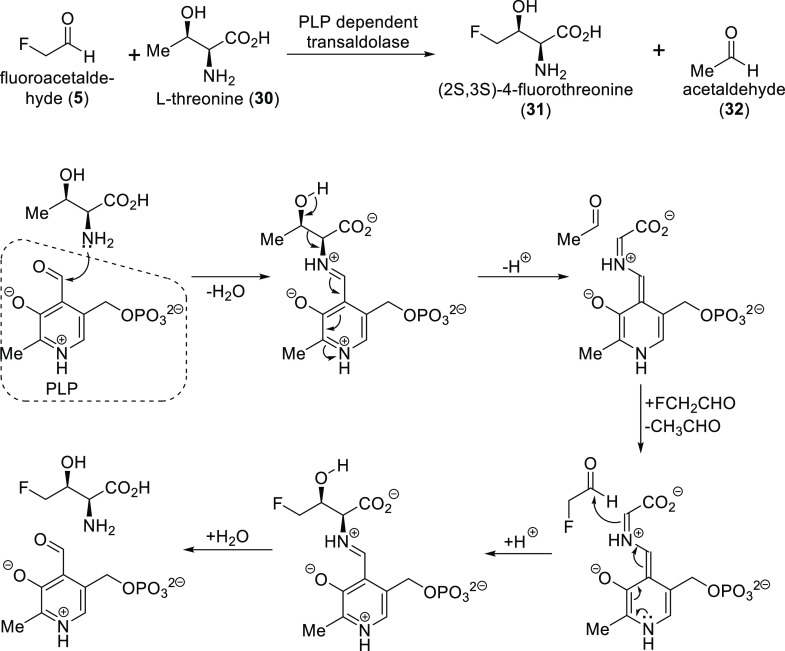
Final Step of (2*S*,3*S*)-4-Fluorothreonine Biosynthesis^,^ See Scheme 1 for previous steps and their detailed mechanism. PLP = pyridoxal phosphate.

The ability of *Streptomyces cattleya* to produce fluoroacetate **6** and (2*S*,3*S*)-4-fluorothreonine **31** was discovered in 1986. (2*S*,3*S*)-4-Fluorothreonine was noticed because of its mild antibiotic activity. As mentioned in Section 2.2, the *S. cattleya* genome is now fully sequenated, and fluorometabolite biosynthesis of this microorganism is completely understood. Production of (2*S*,3*S*)-4-fluorothreonine **31** follows largely the same pathway as that of fluoroacetate synthesis. The only difference is the final step: fluoroacetaldehyde **5** is subjected to a pyridoxal phosphate (PLP) dependent transaldolase together with l-threonine **30** to produce acetaldehyde **32** and (2*S*,3*S*)-4-fluorothreonine **31** (Scheme 6).^24,25^

### 2.6 (2*R*,3*S*,4*S*)-5-Fluoro-2,3,4-trihydroxypentanoic Acid

**Scheme 7:**
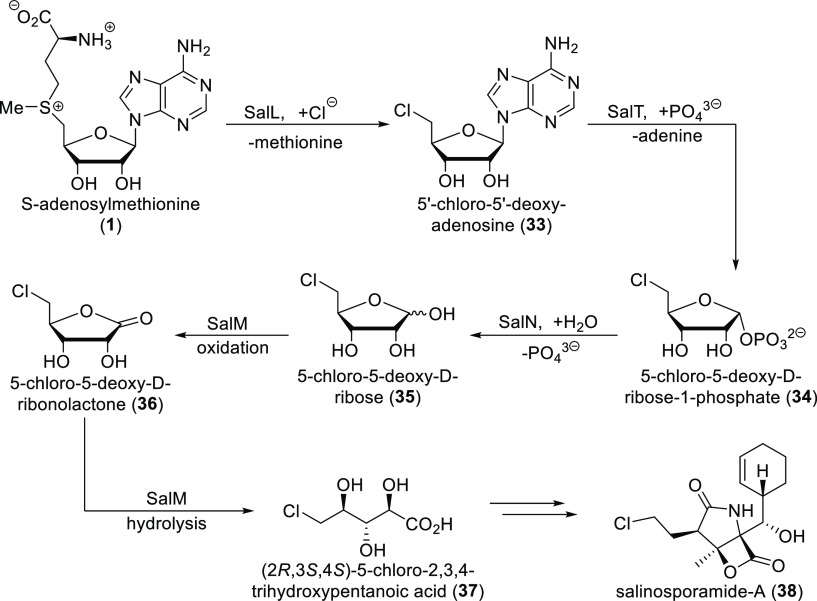
Major Steps of Salinosporamide-A Biosynthesis

After a fluorinase gene was discovered in the sequenced genome of *Streptomyces* sp. MA37, studies of its fluorometabolite production showed that, apart from fluoroacetate and (2*S*,3*S*)-4-fluorothreonine (which were the main organofluorine products), a range of unidentified fluorinated metabolites were also present. Inspired by the salinosporamide-A synthesis of *Salinispora tropica* (see a simplified version in Scheme 7), where 5-chloro-5-deoxy-d-ribose **35** is an intermediate, 5-fluoro-5-deoxy-d-ribose **37** was added to a cell-free extract of *Streptomyces* sp. MA37. This caused some of the fluorometabolites unidentified previously to become the dominant products, suggesting that they are formed via 5-fluoro-5-deoxy-d-ribose. A homologue search of the *Streptomyces* sp. MA37 showed the presence of genes similar to the ones involved in salinosporamide-A production. One of them, *fdrC*, seemed to code a short-chain dehydrogenase analogous to SalM. It oxidizes 5-chloro-5-deoxy-d-ribose **35** into 5-chloro-5-deoxy-d-ribonolactone **36** and possibly catalyzes subsequent lactone hydrolysis during salinosporamide-A production. Indeed, 5-fluoro-5-deoxy-d-ribose **39** was transformed to (2*R*,3*S*,4*S*)-5-fluoro-2,3,4-trihydroxypentanoic acid **41** in vitro in the presence of FdrC and NAD^+^ involving oxidation followed by hydrolysis. ^19^F NMR confirmed that this compound is one of the previously unidentified fluorometabolites of *Streptomyces* sp. MA37.

**Scheme 8:**
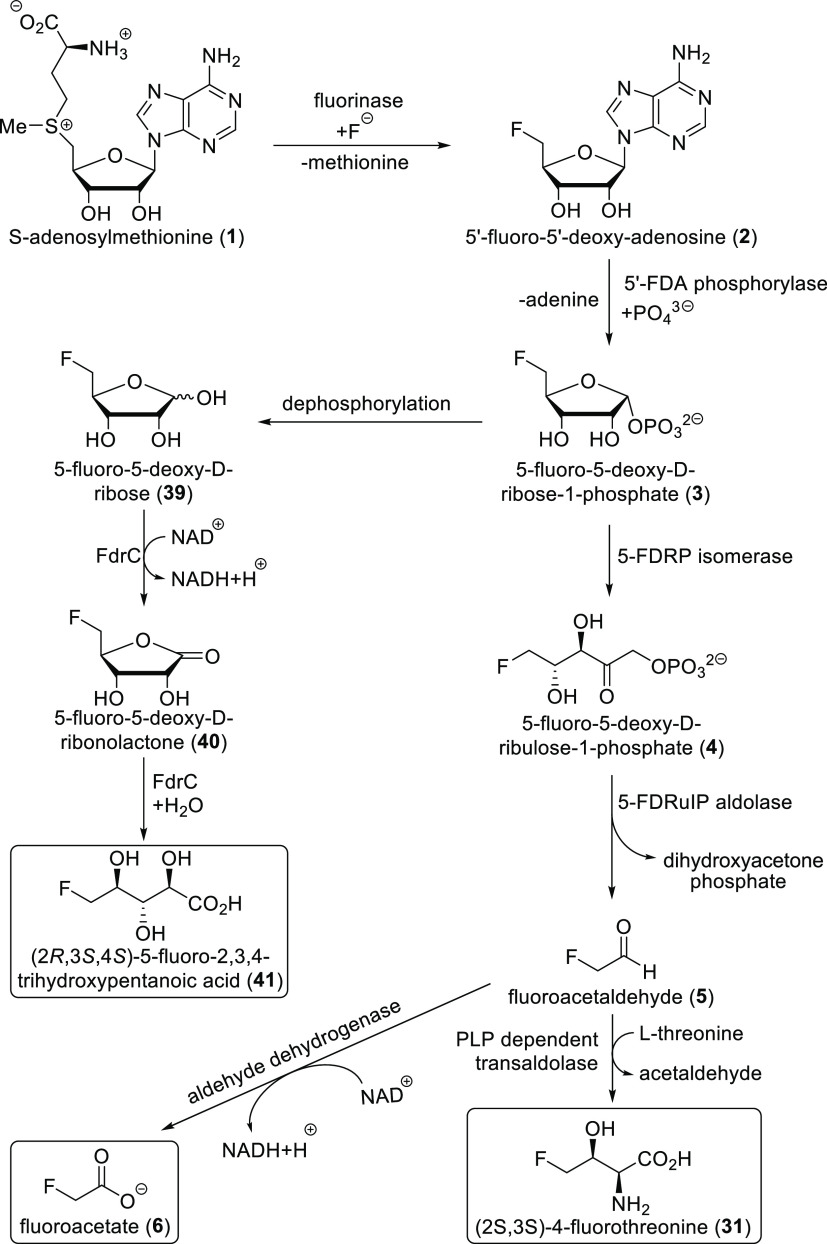
Fluorinase Pathway: Production of Fluoroacetate (**6**), (2*S*,3*S*)-4-Fluorothreonine (**31**), and (2*R*,3*S*,4*S*)-5-Fluoro-2,3,4-trihydroxypentanoic Acid (**41**)

To sum up, the production of (2*R*,3*S*,4*S*)-5-fluoro-2,3,4-trihydroxypentanoic acid is branching from the fluorinase pathway, with 5-fluoro-5-deoxy-d-ribose-1-phosphate **3** being the last common intermediate. Scheme 8 shows the full fluorinase pathway, including the biosyntheses of (2*R*,3*S*,4*S*)-5-fluoro-2,3,4-trihydroxypentanoic acid **41**, (2*S*,3*S*)-4-fluorothreonine **31**, and fluoroacetate (**6**).^24,25,48^

### 2.7 Nucleocidin and Related Fluoroorganic Compounds

**Scheme 9:**
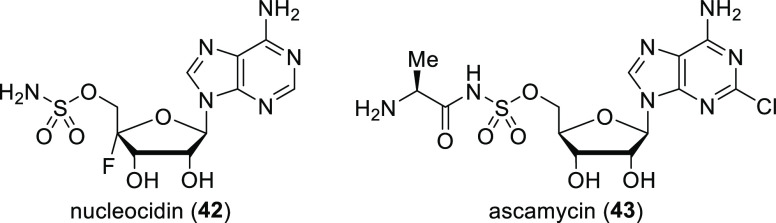
Structure of Nucleocidin and Structurally Related Ascamycin

Nucleocidin (4′-fluoro-5′-*O-*sulfamoyladenosine, **42**, Scheme 9) was isolated in 1957 from *Streptomyces calvus* ATCC 13382, but its fluorinated nature was discovered only in 1969. This compound is a broad-spectrum antibiotic, although it is too toxic for clinical use. The position of the fluorine atom (at C-4 of the ribose moiety) is remarkable because it suggests that nucleocidin is not synthesized from a fluoroacyl molecule. Unfortunately, until 2015, attempts to reisolate nucleocidin from *S. calvus* cultures failed, which seriously hindered the research of its biosynthesis.^24,25,40^

In *Streptomyces* species, disruption of the regulatory genes results in an unusual “bald” phenotype (no sporulation) and deficiencies in secondary metabolite production. The *S. calvus* ATCC 13382 strain exhibits this bald phenotype, and it was discovered in 2013 that its *bldA* gene (which encodes the tRNA for the rare TTA codon) is mutated and nonfunctional. The TTA codon usually concentrates in regulatory and structural biosynthetic genes of *Streptomyces* species; that is, their translation requires a working *bldA*. This explains the loss of nucleocidin production.^24,25,49^

It was shown in 2015 that providing a functional *bldA* copy to this strain restored both sporulation and nucleocidin synthesis. At that time, the 23-membered gene cluster responsible for the synthesis of ascamycin **43** (which has structural similarities to nucleocidin; see Scheme 9) was already known, and a search in the *S. calvus* ATCC 13382 genome found 16 homologous genes, divided between 2 gene clusters. Supported by gene disruption experiments, these were identified as the nucleocidin biosynthetic cluster.^49^ This identification was confirmed further, when a highly similar gene cluster was revealed in *Streptomyces asterosporus* DSM 41452. This microorganism also had a bald phenotype but for a different reason, namely, because of the presence of the nonfunctional pleiotropic regulator gene *adpA*. Complementation with a working *adpA* sequence restored sporulation and promoted the production of nucleocidin, doubling the number of known nucleocidin-producing organisms.^50^

**Scheme 10:**
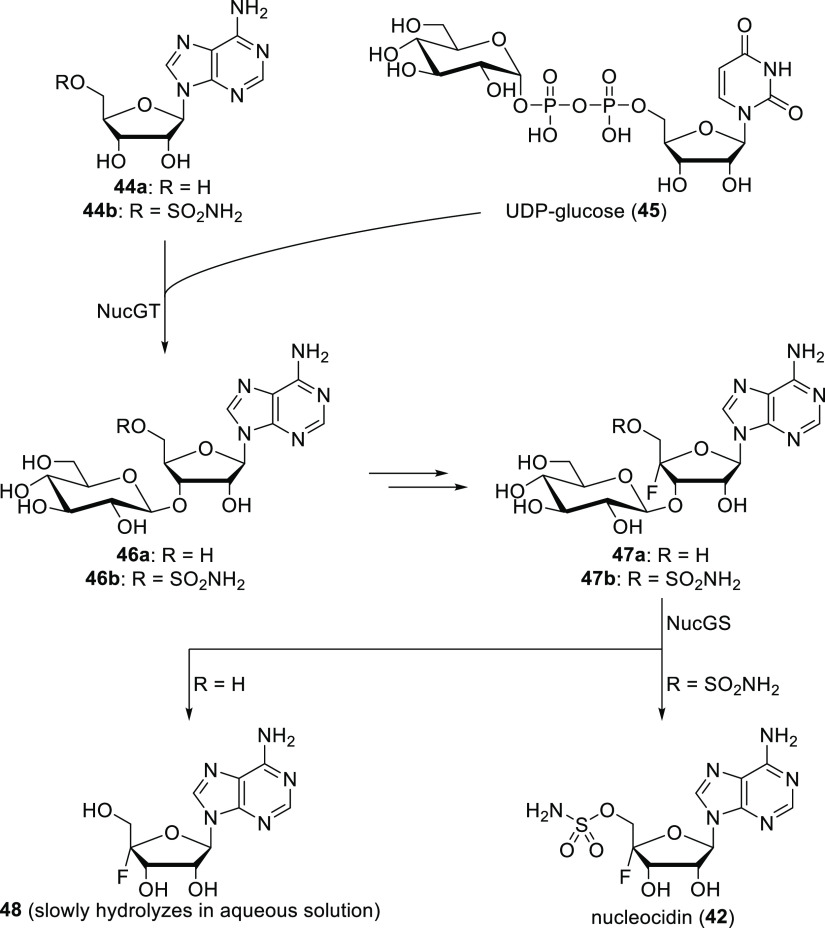
Putative Steps of Nucleocidin Production^,^ Fluorinated compounds **42** and **47a,b** were isolated from the fermentation broth. Reaction of **47a** with recombinant NucGS *in vitro* yielded known compound **48**.

**Scheme 11:**
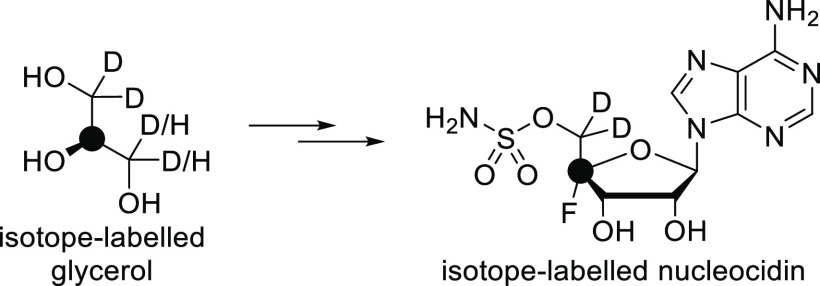
Results of Isotope-Labeling Experiments Glycerol at the left has a virtual labeling pattern, which is a composite of experimentally administered glycerols.

Armed with the knowledge above, some details of the nucleocidin biosynthesis were already uncovered. Importantly, first *S. calvus* produces two unknown fluorometabolites, which then disappear in parallel with the emergence of nucleocidin. These new metabolites were found to be 3′-*O*-β-glucosylated-4′-fluoroadenosine **47a** and 3′-*O*-β-glucosylated nucleocidin **47b**. This directed attention to a glycosyltransferase gene (*nucGT*) and a β-glucosidase gene (*nucGS*) within the biosynthetic cluster. It turned out that NucGS performs the last step of nucleocidin production (deglycosylation of **47b** into nucleocidin), while NucGT is involved in an earlier step (its knockout completely disabled fluorometabolite production). Since 5′-*O*-sulfamoyl-adenosine **44b** is a better substrate of NucGT than adenosine **44a**, it can be assumed that the formation of the sulfamoyl moiety precedes glycosylation. Scheme 10 summarizes related pieces of information.^51^ A purine nucleoside phosphorylase, encoded by the *ORF206* gene of *S. calvus*, was also necessary for fluorometabolite production.^49^ According to isotope-labeling experiments, both C5′ hydrogens of the ribose ring are derived intact from the *pro-R* CH_2_OH group of glycerol. Deuterium labels of the *pro-S* CH_2_OH group of glycerol, in turn, are lost, and the secondary carbon of glycerol becomes the C4′ of nucleocidin (Scheme 11).^52,53^ This shows that the *pro-R* CH_2_OH carbon is not oxidized as glycerol, and it is progressed along the pentose phosphate pathway, incorporating into the ribose moiety of nucleocidin.^52^ Note that the enzyme responsible for fluorine incorporation is still unknown.^51^

### 2.8 Miscellaneous Information

When homogenates of *Acacia georginae* (a known fluoroacetate accumulator) are incubated at 30 °C in the presence of 1 mM fluoride, ATP, and pyruvate, some of the fluoride seemingly disappeared, suggesting the formation of volatile organofluorine compounds. Passing the volatiles through acidic 2,4-dinitrophenylhydrazine solution resulted in the formation of a fluorine-containing 2,4-dinitrophenylhydrazone. It was originally identified as fluoroacetone-2,4-dinitrophenylhydrazone based on its retention time on paper chromatography. However, this method cannot differentiate between 2,4-dinitrophenylhydrazones of fluoroacetone fluoroacetaldehyde.^54,55^ Taking into account that FCH_2_CHO is an intermediate toward FCH_2_COO^–^ in *S. cattleya*, the presence of fluoroacetaldehyde is more plausible than that of fluoroacetone.

In 1960, in an organofluorine-containing oil extracted from *D. cymosum*, a long-chain fatty acid was found with the GC characteristics of ω-fluorooleic acid but without its in vivo toxicity. Possibly, reinvestigation of fluoroacetate-accumulating plants with modern analytical methods would be fruitful.^40^

In the marine sponge *Phakellia fusca*, obtained from the South China Sea, five 5-fluorouracil alkaloids were found. However, their natural product nature was debated. Currently, it seems more plausible that they originate from industrial contamination.^24,25^

## 3 Current Trends in Fluorine-Containing Pharmaceuticals and Agrochemicals

As mentioned in Section 2, fluorine-containing organic compounds are rare in nature. In contrast, organofluorine compounds are quite common among pharmaceuticals and agrochemicals. For example, among the drugs accepted by the FDA in 2019, the ratio of the fluorine-containing ones was 31%.

The current section will start with a summary of the main properties and changes associated with fluorination, which are the reasons behind the high abundance of fluorine within man-made bioactive molecules. This part will be illustrated by case studies of drugs. (Note: some compounds benefit more than one effect of fluorine.) Then, fluorinated agrochemicals will be discussed briefly. Finally, current trends in fluorine-containing pharmaceuticals will be studied through statistical analysis of FDA-approved drugs in the 2007–2019 time period.

### 3.1 Advantages of Fluorinated Drug Molecules

#### 3.1.1 Fluorination and Metabolism. Highlighted: Ezetimibe, Celecoxib, and Alpelisib

After taking a drug, the body tries to remove it via deactivation and excretion. Although it is possible to eliminate a drug unchanged, drug molecules are usually subjected to metabolism before elimination. These transformations often result in detoxification (although many exceptions are known) and usually decrease lipophilicity to facilitate clearance. The most important enzymes involved in drug metabolism are cytochrome P450 monooxygenases which are present mainly in the liver, and increasing the stability to oxidation processes mediated by these enzymes is required frequently during drug development.^12^ A well-established way to achieve this goal is the replacement of metabolically labile hydrogens with fluorines.^12^

The success of this strategy originates from the unique properties of fluorine and its bond with carbon. First, because of the higher bonding energy of the C–F bond (≈441 kJ mol^–1^) compared to the C–H bond (≈414 kJ mol^–1^), hydroxylation of C–H bonds can be blocked by fluorination.^12,56^ Also, the strongly electron-withdrawing nature of fluorine (its Pauling electronegativity is 4, the highest value among the elements on this scale) deactivates fluorinated aromatic rings toward oxidative metabolism.^12^ Hydroxyl and amino groups nearby to fluorine are also more resistant to oxidation (the electron withdrawal of F decreases their Lewis basicity).^56^ The bad leaving group ability of fluoride is also important. Thanks to this, fluorinated compounds rarely behave as alkylating agents.^13,14^ (As an exception, electron-deficient (hetero)aryl fluorides can undergo nucleophilic aromatic substitution reactions through the addition–elimination pathway. E1cb elimination reactions of fluoride can lead to reactive intermediates too; see the case of trifluridine in Section 3.1.6.) Finally, the size (van der Waals radius) of a fluorine atom (1.47 Å) is between the sizes of hydrogen (1.20 Å) and oxygen (1.57 Å). As a result, exchanging a H with F often retains the shape of the molecule (for exceptions, see Section 3.1.5), and improvement of metabolic stability can be achieved without compromising binding to the target protein.^12^

**Scheme 12:**
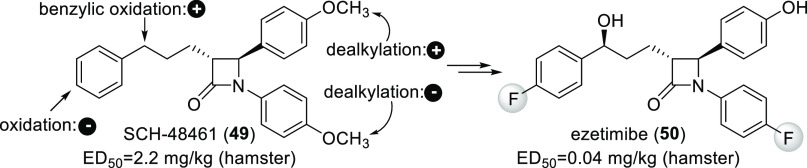
Fluorination Prevents Unwanted Metabolic Changes in the Case of Ezetimibe (**50**)

A good example is the cholesterol absorption inhibitor ezetimibe, a blockbuster drug. (Vytorine, a combination of ezetimibe and simvastatin, was the 18th best selling drug of 2008 in the USA. Zetia, which contains only ezetimibe, was the 30th on the same list.)^16^ During drug development, studying the metabolism of the lead molecule SCH-48461 (**49**) showed that benzylic hydroxylation and demethylation of the 4-methoxyphenyl group attached to the C-4 atom of the azetidinone ring increase potency, while *para* hydroxylation of the phenyl group and demethylation of the 4-methoxyphenyl group attached to the azetidinone nitrogen were nonproductive. The incorporation of beneficial changes and blocking unwanted metabolism by fluorination resulted in ezetimibe (**50**), which was 400 times more potent (Scheme 12).^12^

**Scheme 13:**
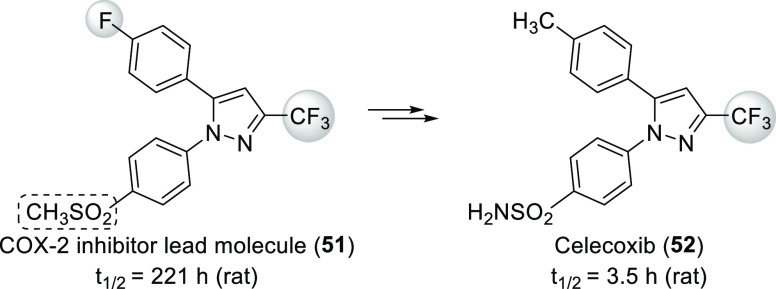
Reducing Excessive Half-Life by Fluorine Removal during the Development of Celecoxib

The extent of metabolic stability which can be achieved is illustrated well by the development of celecoxib, a COX-2-selective nonsteroidal anti-inflammatory drug. This compound was a blockbuster (it was the 20th best selling drug of 2008 in the USA).^16^ Here, the lead molecule **51** had a very long plasma half-life. Replacement of the methylsulfone part with a sulfonamide moiety helped somewhat, but to achieve an acceptable half-life, replacement of a fluorine with a methyl group was necessary (Scheme 13).^57,58^

**Scheme 14:**
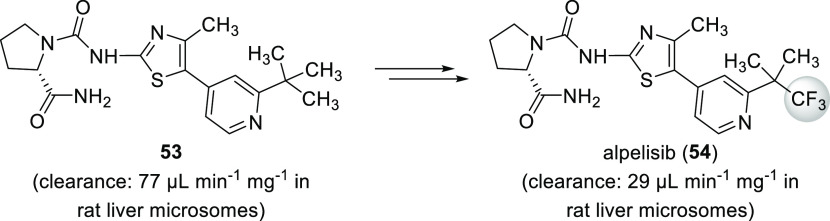
Reducing Clearance by Blocking Aliphatic Hydroxylation During the Development of Alpelisib

A recent example is alpelisib (**54**, Scheme 14). This compound, an α-selective phosphatidylinositol-3-kinase inhibitor, was approved by the FDA in 2019 for treating HR+, HER2-negative, PIK3CA-mutated advanced, or metastatic breast cancer. During its development, compound **53** (Scheme 14) was synthesized. This molecule was an effective and selective inhibitor of p110α, the catalytic subunit of phosphatidylinositol-3-kinase α (in biochemical assays, IC_50_(p110α) = 0.014 μM, IC_50_(p110β) = 4.4 μM, IC_50_(p110δ) = 0.33 μM, and IC_50_(p110γ) = 0.43 μM), but it had high clearance (77 μL min^–1^ mg^–1^ in rat liver microsomes) because of its metabolism (hydrolysis of the CONH_2_ moiety and aliphatic hydroxylation of either the *tert*-butyl group or the methyl substituent of the thiazole ring). Replacement of the *t*Bu group with the 1,1,1-trifluoro-2-methylpropan-2-yl substituent to block unwanted aliphatic hydroxylation yielded alpelisib (**54**). Clearance was significantly reduced (29 μL min^–1^ mg^–1^ in rat liver microsomes), while efficacy and selectivity were not compromised (IC_50_(p110α) = 0.005 μM, IC_50_(p110β) = 1.2 μM, IC_50_(p110δ) = 0.29 μM, and IC_50_(p110γ) = 0.25 μM in biochemical assays). In fact, potency toward p110α increased (see Section 3.1.2 for details).^59^

**Scheme 15:**
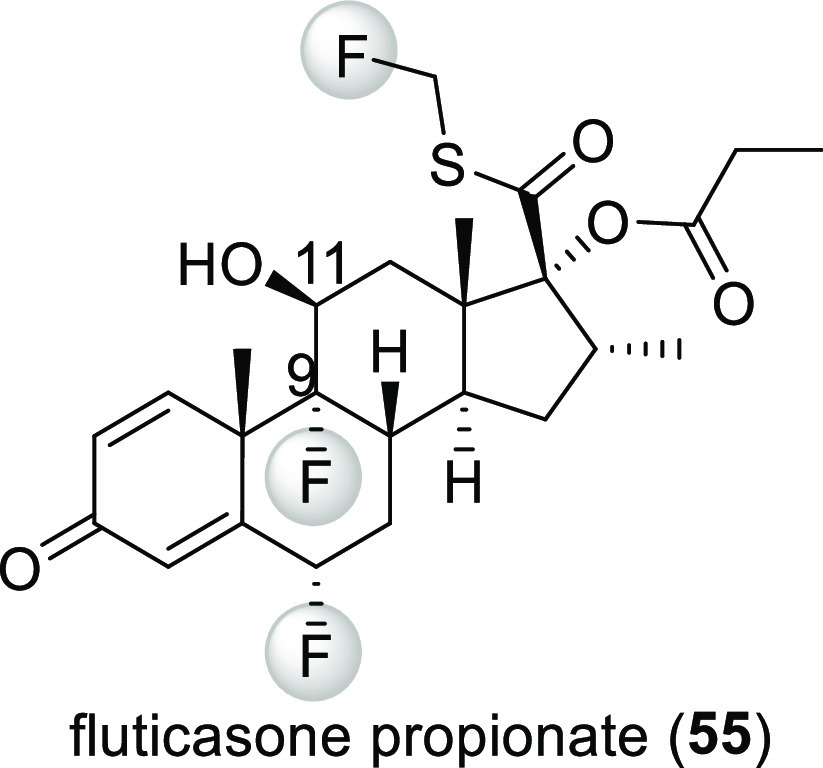
Structure of Fluticasone Propionate

Finally, in fluticasone propionate (**55**, Scheme 15), the fluorine at the 9 position increases the acidity of the hydroxyl group at the 11 position, inhibiting undesirable oxidation here.^56^ This compound is a steroidal anti-inflammatory drug which is mainly used to treat asthma. Advair Diskus, which contains fluticasone propionate and salmeterol, was the fourth best selling drug of 2008 in the USA, making compound **55** another blockbuster drug.^16^

#### 3.1.2 Fluorination and Potency. Highlighted: Type 2 Statins, Sitagliptin and Alpelisib

In contrast with the apolar C–H bond, the C–F bond is highly polarized thanks to the very high electronegativity of fluorine. However, further polarization of C–F bonds is difficult, making the C–F moiety a poor hydrogen bond acceptor. This blocks solvation by water, in contrast to the good hydrogen bond acceptor nature of the fluoride ion. As an overall result, fluorinated organic compounds show polar hydrophobicity and a clear preference for orthogonal multipolar interactions (e.g., C–F···C=O and C–F···H–N) over hydrogen bonding.^12,56,60^

**Scheme 16:**
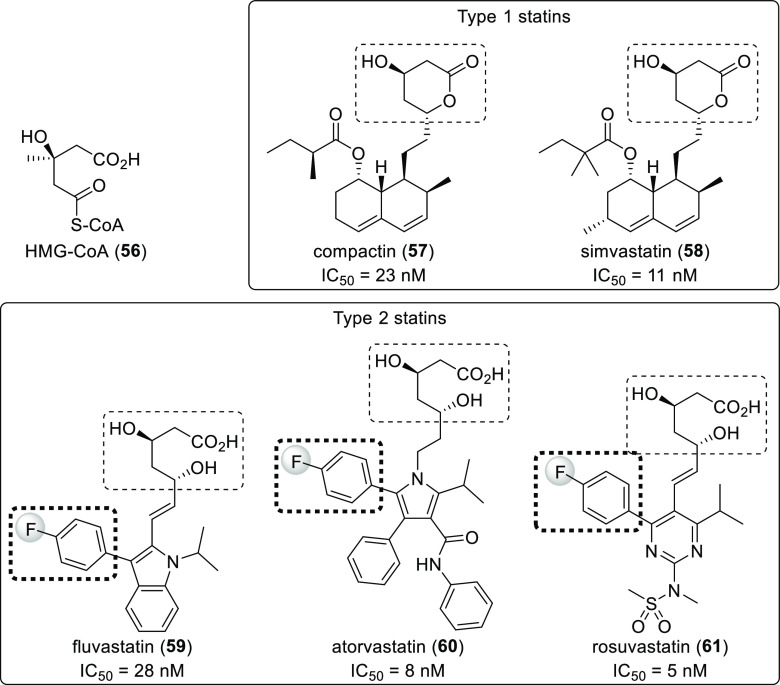
Structure of 3-Hydroxy-3-methylglutaryl-coenzyme A (**56**) and Different Statins^,^ The conserved 3-hydroxy-3-methylglutaryl-like moieties are highlighted with dashed round rectangles. The 4-fluorophenyl croups (the trademarks of type 2 statins) are highlighted with bold dashed round rectangles.

Since such attractive multipolar interactions are absent when fluorine is replaced with a hydrogen, fluorination often increases potency.^12,56,57,60^ Statins (Scheme 16) illustrate this well. These compounds are cholesterol-lowering drugs which inhibit 3-hydroxy-3-methylglutaryl-coenzyme A reductase (or HMG-CoA reductase), a key enzyme in cholesterol biosynthesis. Every statin contains a 3-hydroxy-3-methylglutaryl-like moiety. Type 2 statins also contain a 4-fluorophenyl group, which is absent in Type 1 statins.^61^

**Scheme 17:**
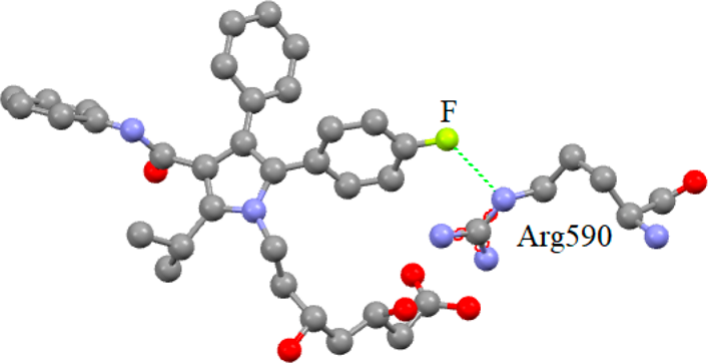
Electrostatic F^δ−^···N^δ+^ Interaction between Atorvastatin and the Arg590 Residue of HMG-CoA Reductase The average fluorine–nitrogen distance is 2.9 Å, lower than the sum of the van der Waals radius of these two atoms.

The partially negatively charged fluorine of this 4-fluorophenyl group forms an energetically favorable electrostatic interaction with the positively charged guanidinium nitrogen of the Arg590 residue within HMG-CoA reductase, thereby improving binding. Scheme 17 shows this for atorvastatin.^61^ It is worth noting that both atorvastatin (the first best selling drug of 2008 in the USA) and rosuvastatin (the 17th best selling drug of 2008 in the USA) were blockbusters.^16^

In Section 3.1.1, it was already mentioned that in fluticasone propionate (**55**, Scheme 15) the fluorine at the 9 position increases the acidity of the hydroxyl group at the 11 position. This also improves its binding to the glucocorticoid receptor.^56^

**Scheme 18:**
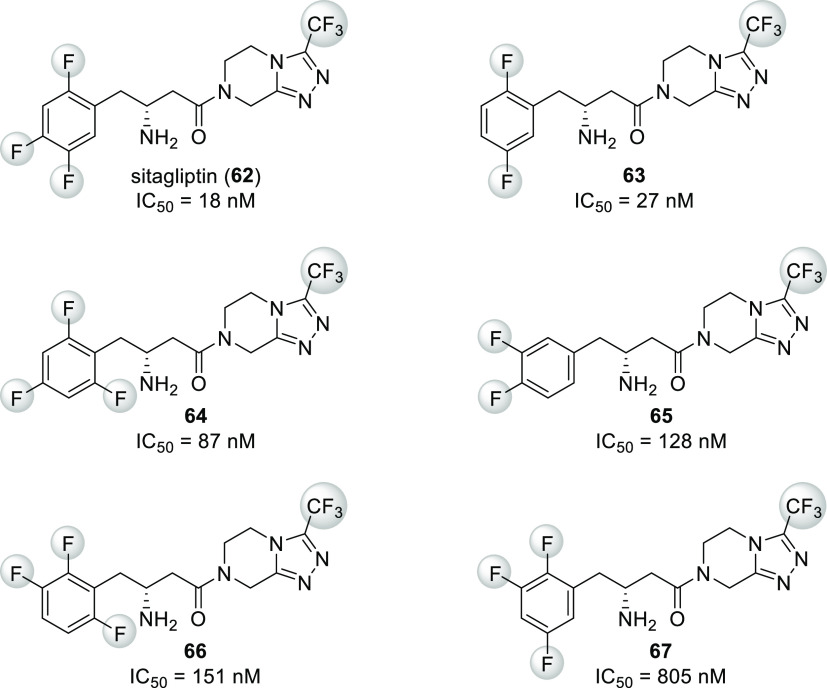
Structure and DPP-IV Inhibitory Activity of Sitagliptin (**62**) and Its Analogues **63**–**67**

Another possibility for enhancing potency via fluorination is when the slightly bigger fluorinated group fits better to a hydrophobic cavity. A good example is sitagliptin (**62**, Scheme 18). This compound inhibits dipeptidyl peptidase IV (DPP-IV), which would rapidly degrade glucagon-like peptide 1 (GLP-1). GLP-1 is released upon food intake, promotes insulin biosynthesis and secretion, and inhibits glucagon release. Increasing its concentration via blocking DPP-IV enables treatment of diabetes with little or no risk of hypoglycemia. As a result, sitagliptin became a blockbuster drug: in 2019, worldwide revenues from Januvia (sitagliptin only) and Janumet (combination of sitagliptin and metformin) were more than 5.5 billion USD.^62^

Evaluation of sitagliptin and its analogues containing different fluorinated phenyl groups showed that the 2,4,5-trifluorophenyl group provides the best IC_50_ value (Scheme 18). This was explained by the X-ray crystal structure of sitagliptin bound to DPP-IV, which showed that the 2,4,5-trifluorophenyl moiety fully occupies the S1 hydrophobic pocket of the enzyme. Notably, the pocket that accommodates the CF_3_ moiety is also quite tight.^58,63^

Another example where fluorination induces better fitting to a hydrophobic cavity is alpelisib (**54**, Scheme 14), which was already discussed in Section 3.1.1. Compared to its analogue **53** (Scheme 14), fluorination not only reduced clearance but also improved inhibitory activity (IC_50_ values against p110α: 0.014 μM for compound **53** and 0.005 μM for compound **54**). This was explained with a docking model which showed that that the *t*Bu moiety in compound **53** does not fully occupy a small cavity because there is still some space around one of its methyl groups.^59^

#### 3.1.3 Fluorination and Bioavailability. Highlighted: Sitagliptin

Fluorination influences bioavailability via modulation of p*K*_a_ values and lipophilicity, a key parameter in pharmaceutical chemistry. The first effect is based on the strong inductive electron withdrawal of fluorine, which makes nearby acidic groups (for example, alcoholic or phenolic OH groups) more acidic and decreases the basicity of nearby basic groups (mostly amino groups). This p*K*_a_ lowering effect shifts the ratio of charged and neutral drug species, affecting binding affinity, bioavailability (for neutral molecules, passive transport through membranes is easier), and lipophilicity (see below).^12,56,57,60^

Lipophilicity is quantified by measuring the partition coefficient (log *P*) of the molecule between octanol (apolar layer) and water (polar layer). When charge states need to be considered, log *D* (the logarithmic coefficient of the distribution of a molecule between octanol and water at a given pH, typically 7.4) is also used.^12^ Usually, proteins are less polar than the surrounding aqueous solution, so some lipophilicity of the ligand is often required for efficient binding. However, too lipophilic molecules tend to have insolubility issues, and during passive transport, they can get trapped in the lipid cores of membranes instead of passing through them.^12,56,57,60^

**Scheme 19:**
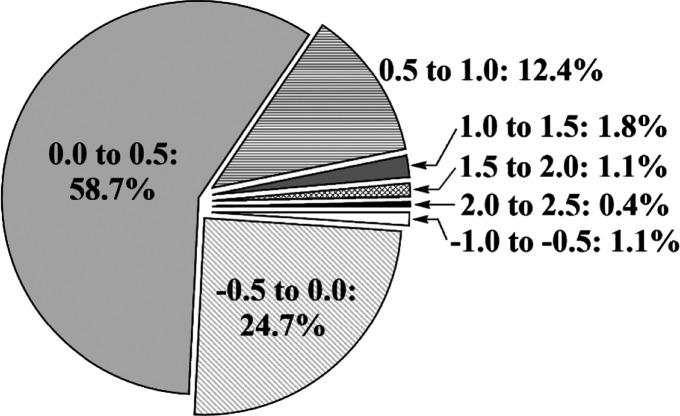
Effect of a Single H → F Exchange on Lipophilicity The log *D*_R–F_ – log *D*_R–H_ difference is shown.

A thorough study on the effect of fluorination on lipophilicity used 283 pairs of molecules from the Roche in-house database which differed by just one fluorine atom (Scheme 19). On average, a H → F exchange increased log *D* with approximately 0.25, but specific groups of molecules behaved differently.^57^ A high log *D* increase was found when fluorine was introduced near to basic nitrogens because the decrease in basicity increased the ratio of the more lipophilic unprotonated amine.^12,56,57^ It is worth noting that introduction of a CF_3_ group also considerably increased log *D*.^12,56^ The most common outcome was an increase of log *D* only with 0 to 0.5. This can be explained by the polar but hydrophobic nature of the C–F bond. However, in a number of cases, a H → F exchange reduced log *D*. In these molecules, fluorine was introduced near to ether, hydroxyl, or carbonyl groups. Possibly, the combined presence of the C–F dipole and the C–O dipole increases the overall polarity of the molecule.^57^ Incorporation of fluorine (polarity) into previously apolar regions (e.g., alkyl chains) also decreases lipophilicity.^12,56,60^

**Scheme 20:**
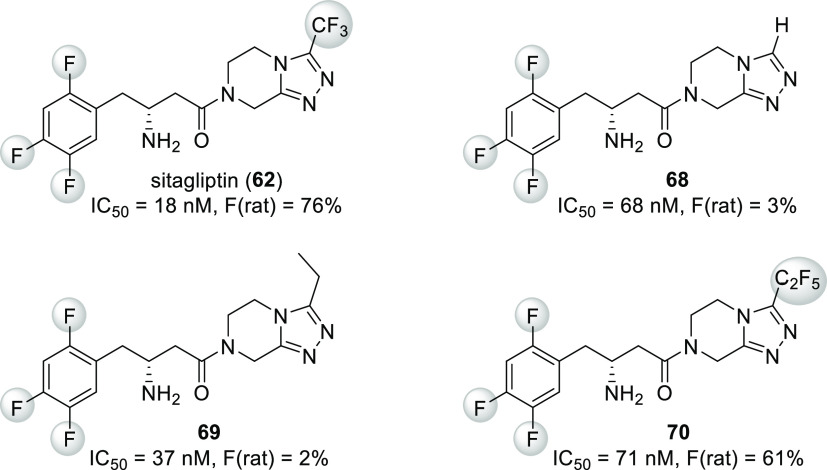
Effect of Triazole Ring Substitution on Oral Bioavailability (F) and DPP-IV Inhibitory Activity of Sitagliptin (**62**) and Its Analogues

Sitagliptin (**62**, Scheme 20) is a good example of improving bioavailability via fluorination. The effect of the fluorination pattern on its phenyl group on potency was already discussed (Scheme 18, Section 3.1.2), but the role of the CF_3_ substituent was not addressed. It was found that replacing it with a hydrogen atom (compound **68**) resulted in almost complete loss of oral bioavailability and some decrease of potency. Replacing the CF_3_ group with an ethyl group (compound **69**) produced a similar outcome. Replacement of the CF_3_ group with the CF_2_CF_3_ group (compound **70**) did not change the oral bioavailability significantly, but it also led to some loss of potency. To sum up (Scheme 20), the most important function of the CF_3_ group was providing good oral bioavailability, although its effect on DPP-IV inhibitory activity was also useful.^58,63^ The latter can be explained by the fact that the CF_3_ group fits tightly into a hydrophobic cavity,^63^ while smaller groups (hydrogen, ethyl)^56^ or larger groups (pentafluoroethyl) do not.

#### 3.1.4 Isosteric Replacement by Fluorine or Fluorinated Moieties. Highlighted: Odanacatib

Because the van der Waals radius of the fluorine atom (1.47 Å) is between the values of hydrogen (1.20 Å) and oxygen (1.57 Å),^12^ fluorine can often be used for isosteric replacement of hydrogens, hydroxyl groups, or methoxy groups. These isosteric replacements usually do not change the shape of the molecule considerably (for exceptions, see Section 3.1.5).^60^

**Scheme 21:**
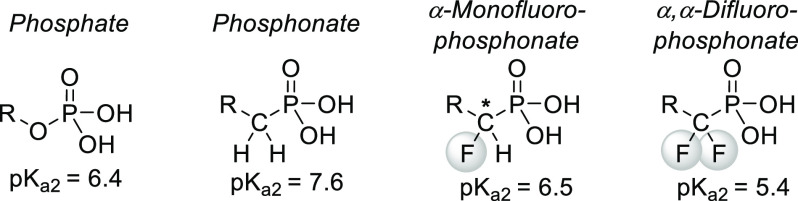
Phosphate Isosteres

It is also possible to replace other functional groups with fluorinated moieties in an isosteric manner. One important example is the case of phosphates. Since phosphorylation is common in biochemistry, nonhydrolyzable phosphate analogues of nucleotides, enzyme substrates, and enzyme inhibitors received great interest. Isosteric replacement of the oxygen linkage with a CH_2_ group yields phosphonates, which cannot be hydrolyzed; however, their second p*K*_a_ value is increased, and the CH_2_ group is ineffective at mimicking the oxygen electronically. α-Monofluorinated and α,α-difluorinated phosphonates, however, are both isosteric and isoelectronic to organophosphates, making them better phosphate mimics. In the case of α-monofluorinated phosphonates, even the p*K*_a2_ value is close to the one found in phosphates (Scheme 21).^56,64,65^

**Scheme 22:**
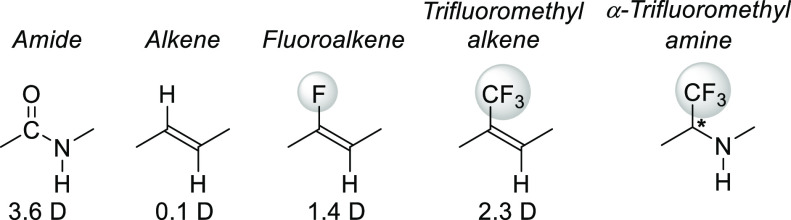
Amide Isosteres

Another important example is the amide group. This highly polar moiety can both accept and donate hydrogen bonds. Its usual problem is its susceptibility to enzymatic hydrolysis. Within nonhydrolyzable amide isosteres which have the appropriate geometry (Scheme 22), *trans*-alkenes have almost no dipole moment, and they are incapable of forming hydrogen bonds. Fluoroalkene moieties are better; however, their dipole moment is only ≈40% of the dipole moment present in amides, and they also lack the ability to form hydrogen bonds. Trifluoromethylated alkene moieties are quite good: they have ≈60% of the dipole moment of amides, and the CF_3_ group electronically mimics the carbonyl oxygen well despite its considerably larger size. However, this isostere still cannot form hydrogen bonds. α-Trifluoromethyl amines are quite promising amide isosteres: the CF_3_ group mimicks the carbonyl oxygen, and its strong inductive electron withdrawal decreases the basicity of the nitrogen atom. As a result, the NH group is relatively nonbasic and has excellent hydrogen bond forming ability.^56,58,60^

**Scheme 23:**
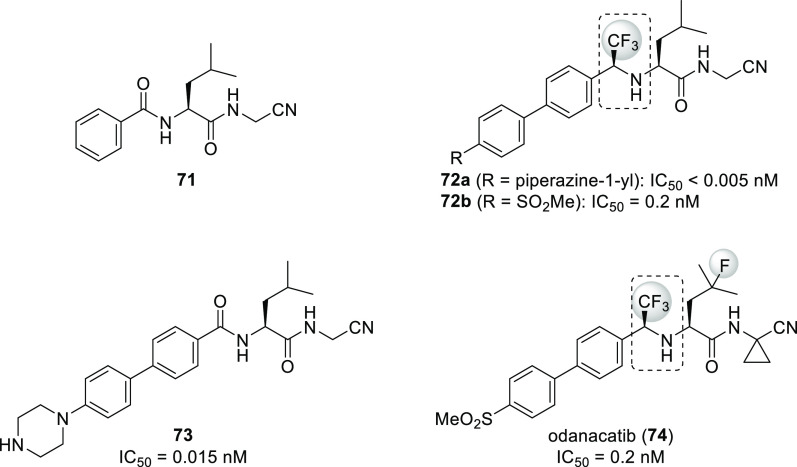
Use of α-Trifluoromethyl Amines (Highlighted with Dashed Round Rectangles) as Amide Isosteres Among Cathepsin K Inhibitors

Good examples for the use of the α-trifluoromethyl amine isostere are cathepsin K inhibitors **72a**–**b** and **74** (odanacatib). Cathepsin K, a lysosomal cysteine protease, seems to be the most important enzyme involved in osteoclastic bone resorption, and its inhibitors were promising for the treatment of osteoporosis. As shown on Scheme 23, compounds **72**–**74** originate from molecule **71**. Incorporation of the α-trifluoromethyl amine isostere and the 4-(piperazin-1-yl)phenyl group resulted in compound **72a**, an extremely potent cathepsin K inhibitor. It was more active than its peptide analogue **73**. Interestingly, the remaining amide bond in **72a** was resistant to hydrolysis. Unfortunately, thanks to the basic piperazine ring, compound **72a** accumulated in lysosomes, which decreased its cathepsin K selectivity in cell-based assays compared to the values measured in enzyme assays. Replacement of the piperazine ring with a methylsulfonyl group led to compound **72b**, which was highly selective even in cell-based assays but had a short half-life in monkeys.^56,58,66^ Incubations in human hepatocytes showed that the major cause was hydroxylation on the methine of the leucine side chain, while the minor cause was hydrolysis of the amide bond. Blocking the major pathway by H → F exchange and the minor pathway by incorporation of a cyclopropane ring yielded odanacatib (**74**), which was subjected to clinical development.^66^ Unfortunately, Phase 3 studies showed that although odanacatib is effective against osteoporosis it also increases the risk of stroke, and development was discontinued.^67^

It is also worth noting that fluorinated arenes can be used as nucleobase isosteres. Notably, several high-fidelity DNA polymerases were able to incorporate nucleotide mimics containing fluoroarene moieties into DNA.^56^

#### 3.1.5 Conformational Changes Triggered by Fluorine Incorporation

Although isosteric replacement of H, OH, or OMe with F generally does not affect the conformations of the molecule too much, stereoelectronic effects originating from the very high electronegativity of fluorine can overwrite this.

**Scheme 24:**
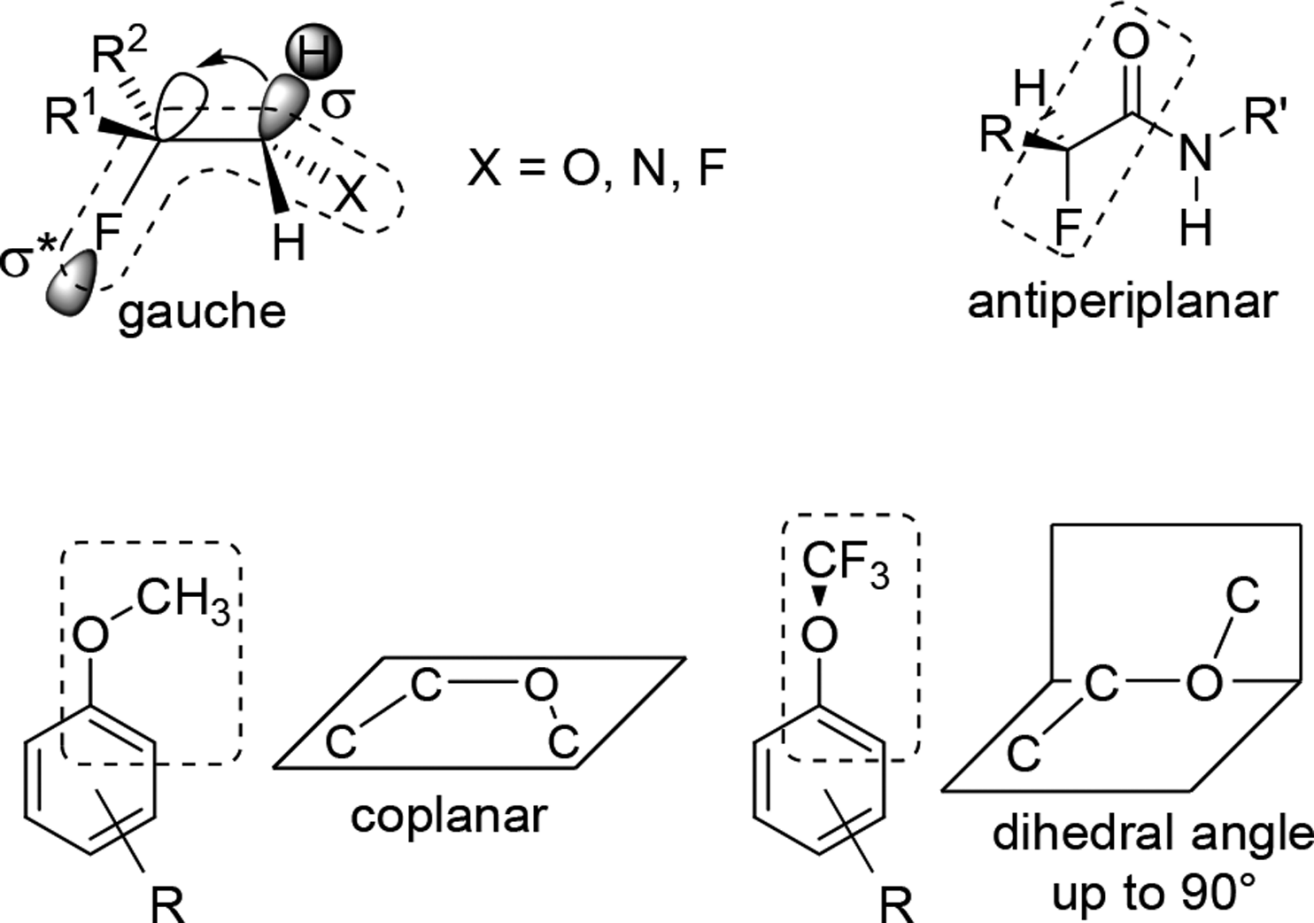
Stereoelectronic Factors Associated with Fluorination

Usually, vicinally disubstituted alkanes adopt antiperiplanar conformation to maximize the distance between the substituents (minimizing steric repulsion). However, in vicinal difluorides, 2-fluoroamines, 2-fluoroalcohols, and 2-fluoroalkyl ethers, *gauche* geometry of the two heteroatoms is favored. In this way, the C–F bond is antiperiplanar with a C–H bond, and the filled σ_C–H_ orbital can donate some electrons to the lower-lying vacant σ*_C–F_ orbital (Scheme 24). The energy gain from this hyperconjugation effect compensates for the steric repulsion between the small fluorine and the other heteroatom, as long as the other atom is not too big (1-fluoro-2-chloroethane prefers the *anti* conformation).^56,68−71^

Study of fluorinated amino acids demonstrated that in α-fluoro amides antiperiplanar arrangement of the carbonyl oxygen and the vicinal fluorine is preferred (Scheme 24). Its main causes seem to be dipole effects. Since fluorinated amino acids also contain amino nitrogens, this effect can occur together with the gauche effect, yielding conformationally restricted amino acids which are quite useful in peptide and foldamer chemistry to achieve specific secondary structures.^68−71^ They can also be applied to study conformational effects in peptides. For example, in natural collagens, many proline residues are oxidized to (4*R*)-hydroxyproline residues which stabilize the triple helix structure. Originally, it was suspected that the hydrogen bond donor/acceptor properties of the introduced OH groups are responsible for the stabilization. However, replacing (4*R*)-hydroxyproline residues with (4*R*)-fluoroproline residues (which cannot participate in such hydrogen bonding) resulted in more stable triple helices, and it was discovered that the effect of proline hydroxylation is based on changing the conformation of the proline ring via stereoelectronic effects.^72,73^

Trifluoromethoxybenzenes provide a well-known example for fluorination-induced conformational changes. Compared to methoxybenzenes, which have a coplanar structure (unless they are *ortho*-disubstituted), trifluoromethoxybenzenes clearly prefer a nonplanar structure (Scheme 24).^12,57,60^

Among recently approved drugs, sonidegib^74^ and pretomanid^75^ contain 4-trifluoromethoxyphenyl moieties. The X-ray crystal structure of the latter clearly shows the nonplanar structure of this moiety.^76^ Unfortunately, the advantage of the CF_3_O group over the CH_3_O group cannot be determined *unequivocally from the published data*.

#### 3.1.6 Fluorination- and Mechanism-Based Inhibitors. Highlighted: 5-Fluorouracil and Trifluridine

A mechanism-based enzyme inhibitor is a compound which is inactive in itself, but the target enzyme’s normal working converts it to an active species. This reactive intermediate then either binds strongly to the target enzyme or forms a covalent bond with it.^77^ Fluorination can be used to create mechanism-based inhibitors for two reasons. On one hand, fluorine and hydrogen are isosteric, so after the H → F exchange the new compound usually remains a substrate of the target enzyme. On the other hand, chemical properties of hydrogen and fluorine are very different: hydrogens in the α-position relative to conjugatively electron-withdrawing groups can be removed as protons, while fluorines can be removed as fluoride ions (usually via E1cb elimination).

**Scheme 25:**
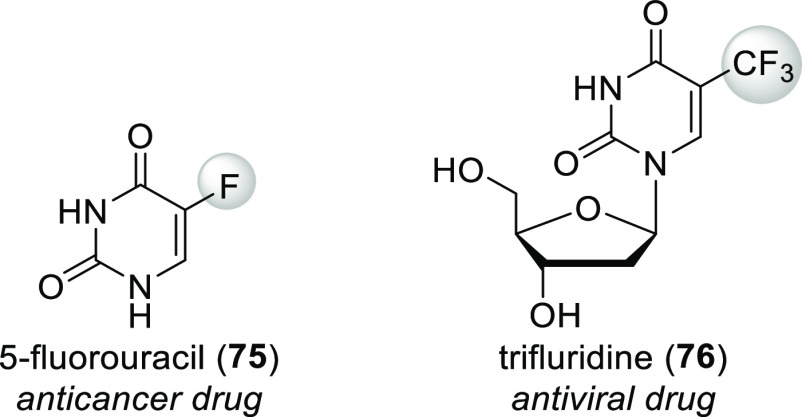
Structure of 5-Fluorouracil (**75**) and Trifluridine (**76**), Mechanism-Based Inhibitors of Thymidylate Synthase

**Scheme 26:**
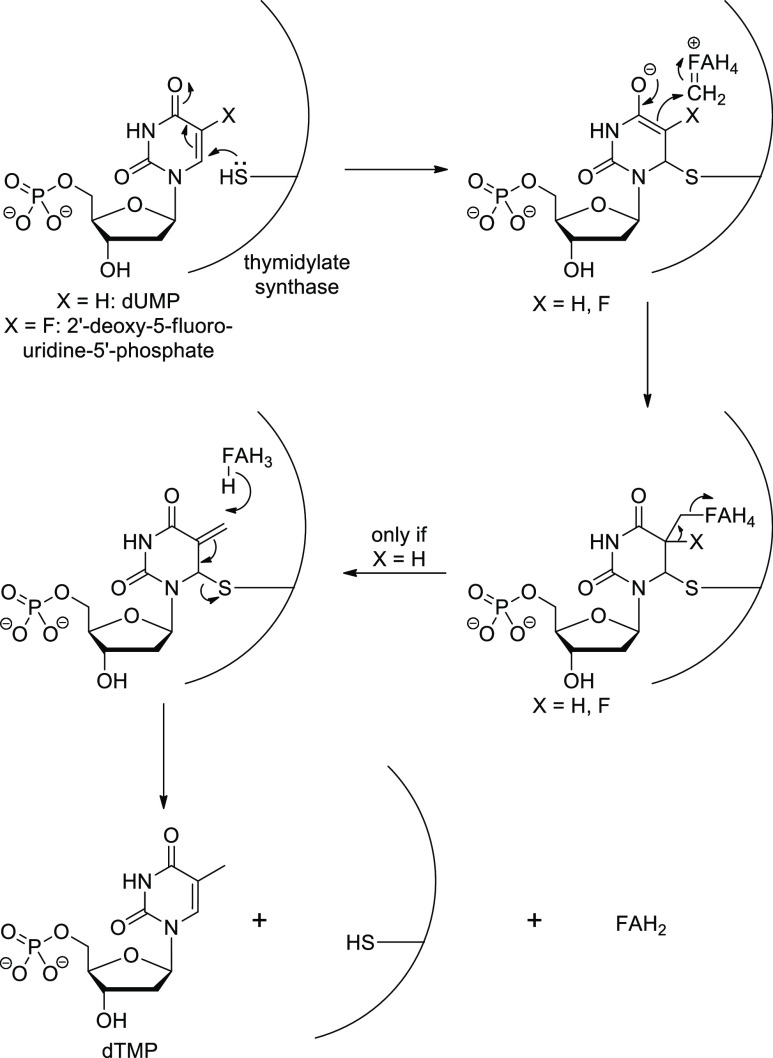
Mechanism of Thymidylate Synthase and Its Inhibition by 2′-Deoxy-5-fluorouridine-5′-phosphate The active metabolite of fluorouracil **75**. CH_2_=FAH_4_^+^ = methylenetetrahydrofolate, FAH_4_ = tetrahydrofolate, FAH_2_ = dihydrofolate, dUMP = deoxyuridine monophosphate, dTMP = deoxythymidine monophosphate.

Taking the above facts into account, one way of using fluorination to create a mechanism-based inhibitor is to find a hydrogen which has to be removed as a proton during the enzyme mechanism and replace it with fluorine. As a result, the enzymatic transformation will be stopped midway. A good example of this strategy is 5-fluorouracil (**75**, Scheme 25), one of the most ancient fluorinated drug molecules (it was discovered and developed in the 1950s as an anticancer drug). This compound inhibits thymidylate synthase, which is necessary for DNA biosynthesis, causing apoptosis of rapidly dividing cells (e.g., cancer cells). Normally, thymidylate synthase forms a ternary complex with 2′-deoxyuridine-5′-phosphate and methylenetetrahydrofolate (CH_2_=FAH_4_^+^). After Michael addition of the active site cysteine thiolate to the uracil ring, the resulting enolate alkylates methylenetetrahydrofolate. This is followed by 5-deprotonation of the uracil ring, elimination of tetrahydrofolate, hydride ion transfer from tetrahydrofolate to the exomethylene group of the intermediate, and release of 2′-deoxythymidine-5′-phosphate (Scheme 26). During analogous transformation of 2′-deoxy-5-fluorouridine-5′-phosphate (formed in vivo from 5-fluorouracil), 5-deprotonation of the uracil ring is impossible (there are not any hydrogens at that position), and the reaction cannot proceed further (Scheme 26).^12,78^

**Scheme 27:**
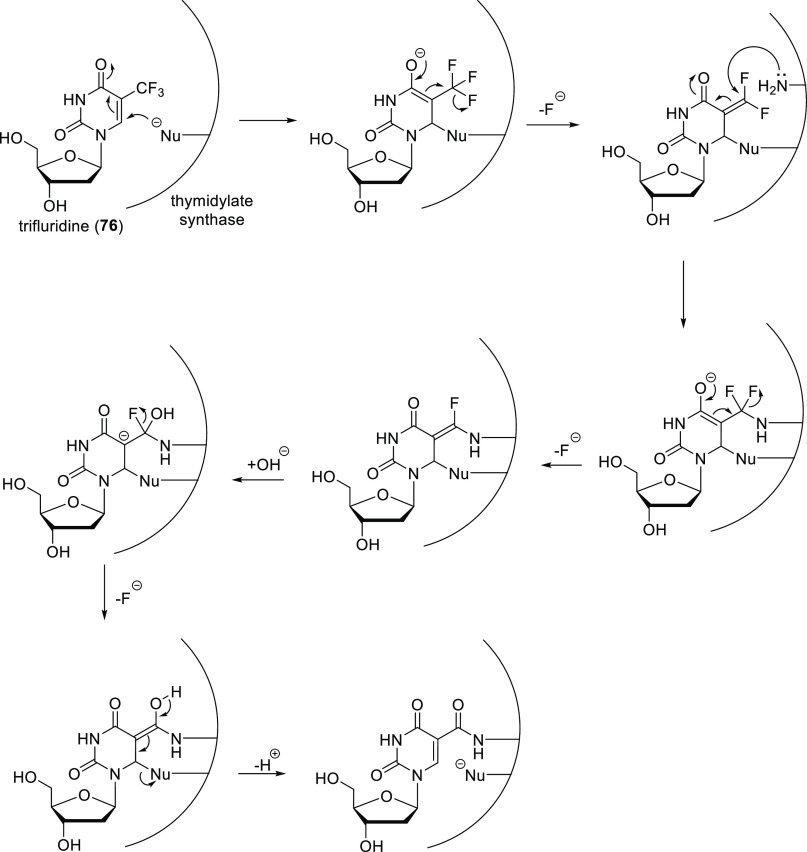
Inhibition of Thymidylate Synthase by Trifluridine

The other way of using fluorination to create a mechanism-based inhibitor is incorporation of fluorine in such a way that during enzymatic transformation of the fluorinated molecule a reactive intermediate is formed via E1cb elimination of fluoride. Then, this reactive intermediate undergoes conjugate addition with nearby nucleophilic groups of the enzyme, binding to it covalently (irreversibly). A good example of this strategy is trifluridine (**76**, Scheme 25), another thymidylate synthase inhibitor which is used to treat viral infections of the eye. During its transformation by thymidylate synthase, the first formed enolate loses a fluoride ion instead of alkylating methylenetetrahydrofolate. The resulting β,β-difluorinated α,β-unsaturated amide is a good Michael acceptor and reacts with a nearby nucleophilic amino side chain of the enzyme. Loss of the second fluoride ion and subsequent hydrolysis result in irreversible binding to thymidylate synthase via a peptide bond (Scheme 27).^12^

### 3.2 Agrochemicals

Within new active ingredients which were provisionally approved by ISO between 1998 and 2008 and used as agrochemicals, 78.5% contained halogens.^19^ Furthermore, in the time frame 1940–2003, ≈28% of all commercially available halogenated products contained fluorine.^18^ This conclusion is supported by the findings of more recent surveys.^20,79^

The main effects of fluorination on agrochemicals and drugs should be the same. However, since agrochemicals are applied externally, chemical degradation should also be taken into account in addition to biological degradation. It is also worth noting that the literature about the development and structure–activity relationships of fluorine-containing agrochemicals^18−20,80,81^ is scarce compared to the analogous literature of fluorinated drugs. Even in the case of well-defined structure–activity relationships, the molecular background behind the beneficial effect of fluorine is often missing.^18−20,80,81^

#### 3.2.1 Fluorination and Degradation

**Scheme 28:**
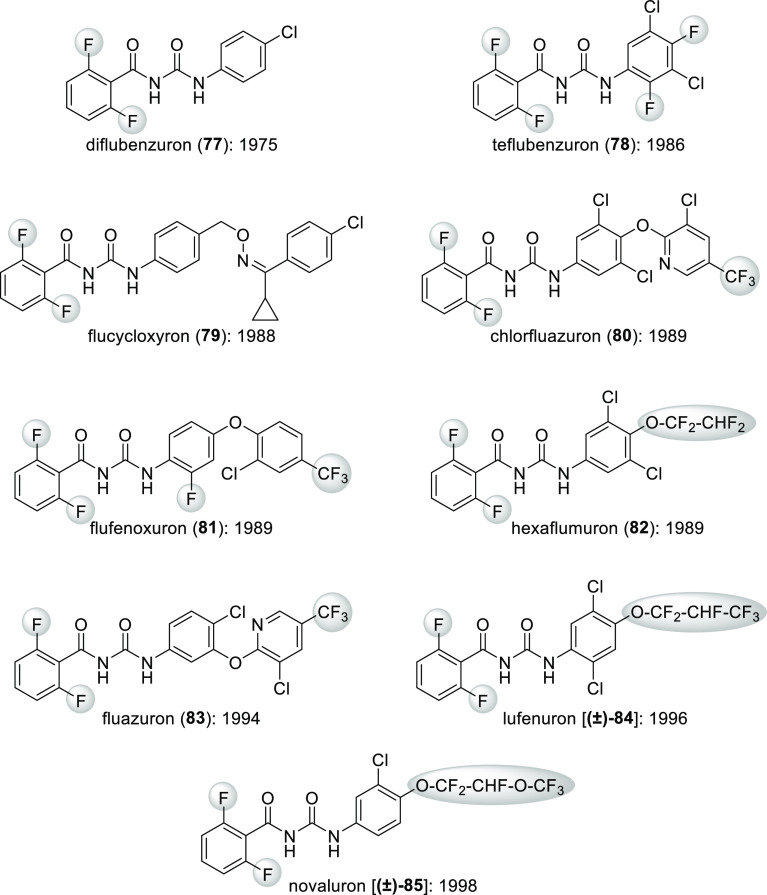
Commercial Insect Growth Regulators Belonging to the *N*-2,6-Difluorobenzoyl-*N*′-phenyl Urea Compound Family The year of their commercial introduction is also given.

**Scheme 29:**
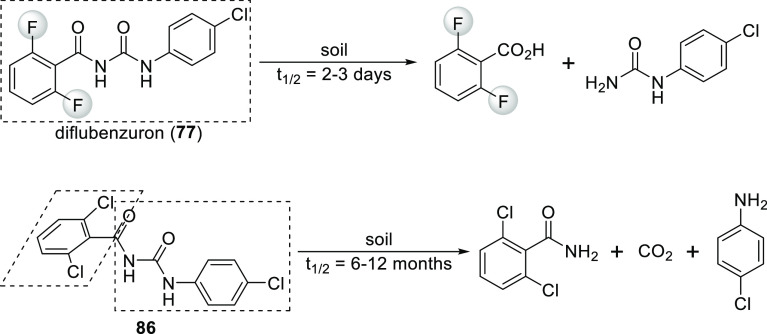
Comparison of Diflubenzuron (**77**) and Its Chlorine-Containing Analogue **86**: Decomposition in Soil and Conformations Dashed rectangles highlight coplanar moieties.

Within commercial insect growth regulators, *N*-benzoyl-*N*′-phenyl ureas are an important compound family. These compounds are acting as chitin formation inhibitors. Notably, many of them contain a 2,6-difluorobenzoyl moiety (Scheme 28). Originally, the reason behind fluorine introduction was reduction of environmental stability to avoid persistence: the half-life of chlorinated **86** in soil is 6–12 months, while the half-life of its fluorinated analogue, diflubenzuron (**77**), is only 2–3 days under the same conditions (Scheme 29). It was showed that as a result of the size difference of F and Cl (F is isosteric with H, while Cl is quite big) the 2,6-difluorobenzoyl moiety is in plane with the rest of the urea structure, while the 2,6-dichlorobenzoyl moiety is perpendicular to it (Scheme 29). This causes the difference between the environmental stability and degradation pathway of **86** and diflubenzuron (**77**).^18^ Later studies also found that the 2- and 6-substituents of the *N*-benzoyl moiety in these ureas should be small, hydrophobic, and electron-withdrawing groups for optimal bioactivity. So fluorination was beneficial for activity too.^81^

#### 3.2.2 Fluorination and Activity

Since studying structure–activity relationships is an easy and effective way to determine the effect of fluorination on activity, many examples are known where fluorination improved bioactivity. However, the molecular background of this effect is often missing.^18−20,80,81^

**Scheme 30:**
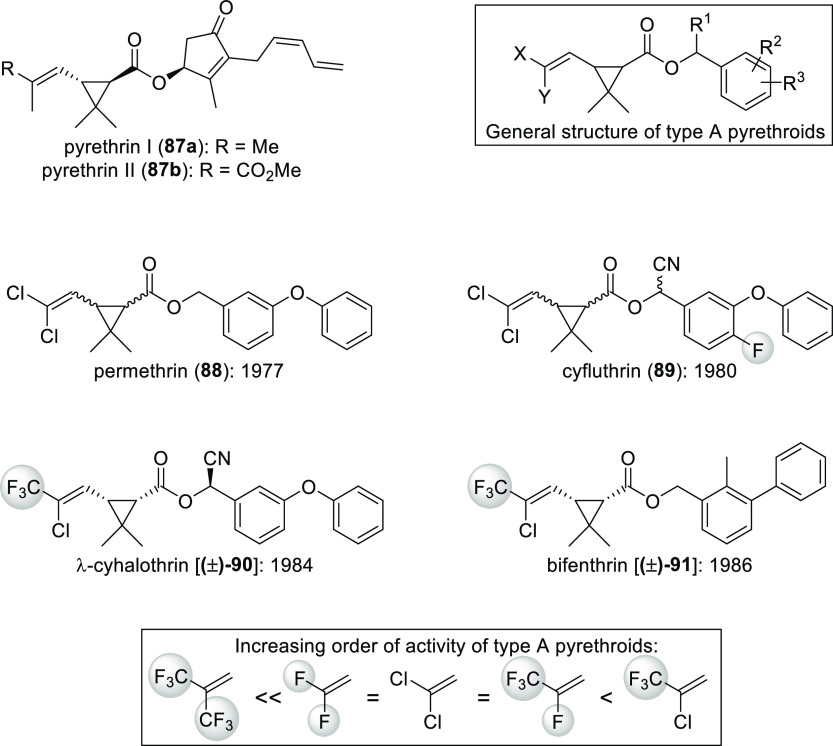
Structure–Activity Relationship of Type A Pyrethroids and Some Examples Year of commercial introductions are also given.

Type A pyrethroids, which are analogues of the natural compounds pyrethrin I (**87a**) and pyrethrin II (**87b**), are good examples.^19^ Both natural and synthetic pyrethroids are potent insecticides. It was found that placement of a 2-chloro-3,3,3-trifluoroprop-1-en-1-yl group on the cyclopropyl ring gave the highest activity. Incorporation of 2,3,3,3-tetrafluoroprop-1-en-1-yl, 2,2-dichlorovinyl, and 2,2-difluorovinyl gave comparable results, while the 3,3,3-trifluoro-2-(trifluoromethyl)prop-1-en-1-yl was the least effective within the groups above. These data together with the structure of some commercially available pyrethroids as an illustration are summarized in Scheme 30.^18,19^

**Scheme 31:**
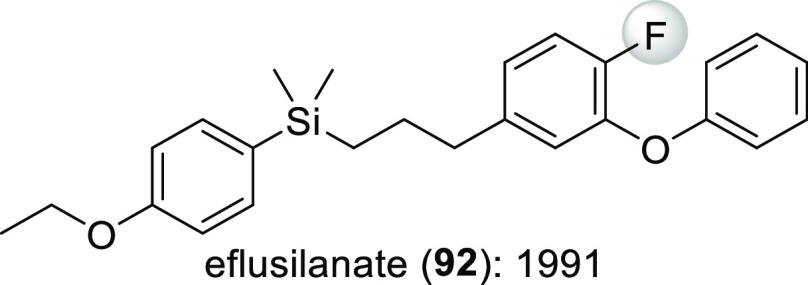
Type C Pyrethroid Eflusilanate: Structure and Year of Commercial Introduction

Eflusilanate (**92**, Scheme 31) is a type C pyrethroid introduced to the market in 1991.^19^ Removal of its fluorine atom results in 10-fold loss of insecticide activity.^80^

**Scheme 32:**
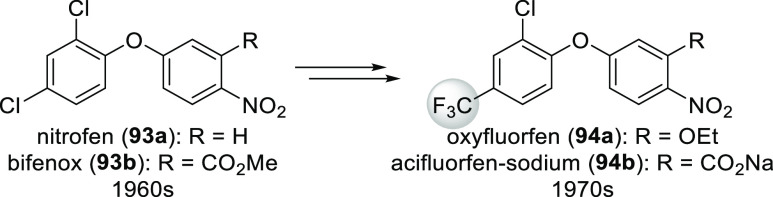
Evolution of Diphenyl Ether Herbicides

Diphenyl ether herbicides (protoporphyrinogen-IX-oxidase (PPO) inhibitors) also illustrate well the impact of fluorination.^20,80^ The first members of this compound family, nitrofen (**93a**) and bifenox (**93b**), were introduced in the 1960s, but they did not have a significant role in the control of weeds in commercial crops. The most important step toward changing this situation was replacing one of the chlorines with a trifluoromethyl group. After further minor optimation, oxyfluorfen (**94a**) and acifluorfen sodium (**94b**) were introduced in the 1970s (Scheme 32). Compounds **94a**,**b** were much more potent than their predecessors, and they were able to control a wider spectrum of weeds.^80^

**Scheme 33:**
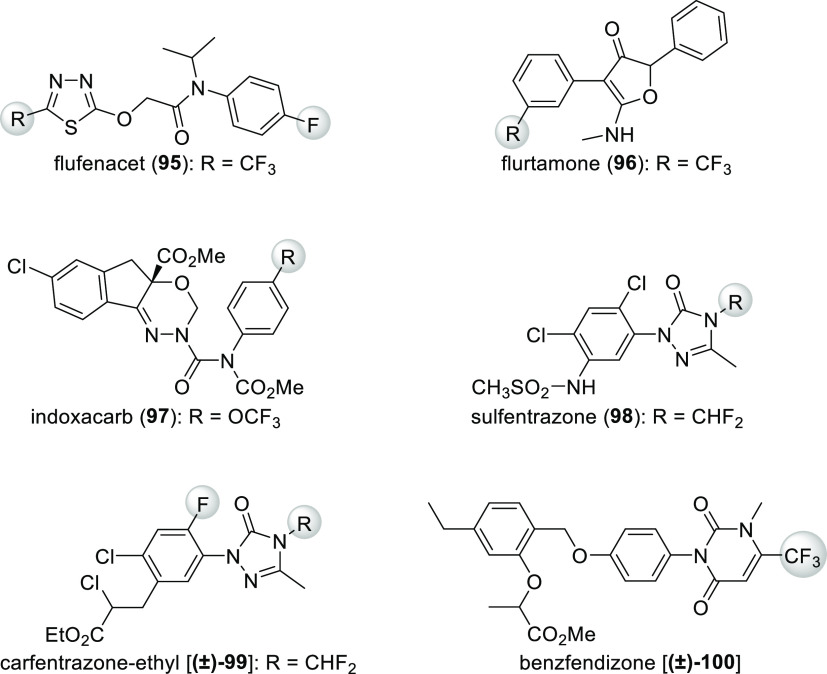
Examples of Agrochemicals Where Fluorination Enhanced Potency

Scheme 33 shows some other examples where fluorine incorporation improved activity. In the case of herbicide flufenacet (**95**, R = CF_3_), a cell growth, and division inhibitor, the relationship between the R group and the activity was Cl < CHF_2_ < CF_3_.^18^ For flurtamone (**96**, R = CF_3_), a herbicide which works by inhibiting phytoene desaturase (disrupting the synthesis of carotinoids),^19^ the same relationship was H ≪ OCH_3_< Cl < CF_3_.^80^ The same *meta*-(trifluoromethyl)phenyl group can be found in many other herbicides with the same mechanism of action.^18^ In the case of indoxacarb (**97**, R = OCF_3_), an insecticide which acts by blocking the sodium channel, the relationship between the R group and the activity was F < Cl < OCHF_2_ ≈ Br < CF_3_ < OCF_3_.^80^ Among analogues of the protoporphyrinogen-IX-oxidase (PPO) inhibitor herbicides sulfentrazone (**98**, R = CHF_2_) and carfentrazone-ethyl [**(±)-99**, R = CHF_2_], activity as a function of the R group was Me < CH_2_F < Et ≈ *i*Pr ≪ CHF_2_.^80^ Finally, replacement of the CF_3_ group of the protoporphyrinogen-IX-oxidase (PPO) inhibitor herbicide benzfendizone [**(±)-100**] with a methyl group completely eliminates bioactivity.^80^

#### 3.2.3 Fluorination and Lipophilicity

**Scheme 34:**
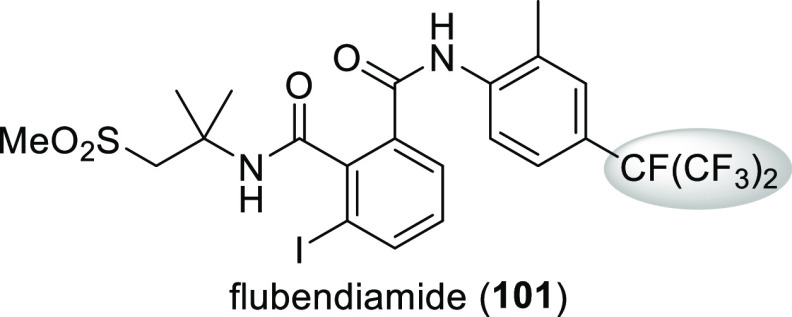
Structure of Insecticide Flubendiamide

Flubendiamide (**101**, Scheme 34), an insecticide with a novel mechanism of action (it activates ryanodine-sensitive intracellular Ca^2+^ release channels in insects but not in mammals), contains a unique heptafluoroisopropyl group.^80^ The main reason for its introduction was its lipophilicity.^81^

### 3.3 Observable Trends Related to Fluorinated Bioactive Compounds

#### 3.3.1 Pharmaceuticals

**Scheme 35:**
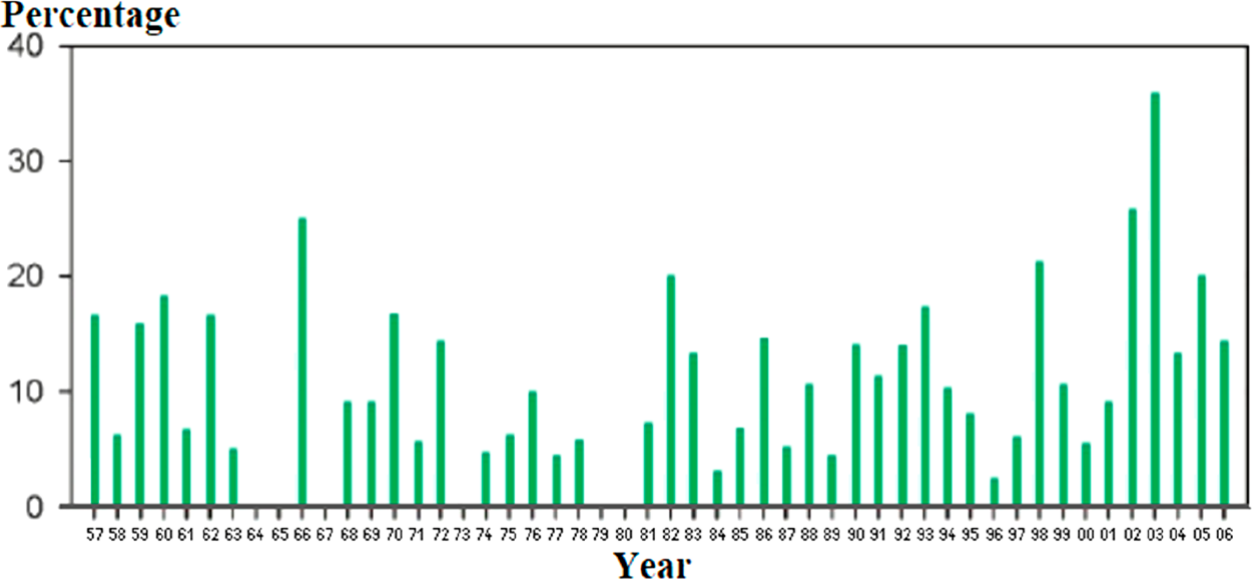
Yearly Percentages of Fluorine-Containing Drug Molecules within Drugs Launched Worldwide in that Year^,^ Excluding biologics, inorganics, reformulations, and agricultural agents. Adapted from ref (58) with some modifications. Copyright 2008. American Chemical Society.

To investigate the advance of fluorination in the pharmaceutical industry, the method described below was followed. For every year, the ratio of fluorine-containing drug molecules within drugs launched in that year was calculated. Then, these ratios were displayed as a function of time. For the time frame 1957–2006, William K. Hagmann already performed these steps with the help of an MDL Drug Data Report on drugs launched worldwide (biologics, inorganics, reformulations, and agricultural agents were omitted).^58^ His data are shown in Scheme 35. Although there is a considerable and seemingly random oscillation in these percentages, there is still evidence to the increasing prevalence of fluorinated drugs. Since 1981, fluorine-containing drugs were launched in every investigated year (previously, this was not the case). Also, from the six cases when the yearly percentages of fluorine-containing drugs reached 20% or higher, four happened in the last 10 years of the 49 year long time period. One of these cases, the year 2003, has the highest yearly percentage between 1957 and 2006 (well above 35%).^58^

**Scheme 36:**
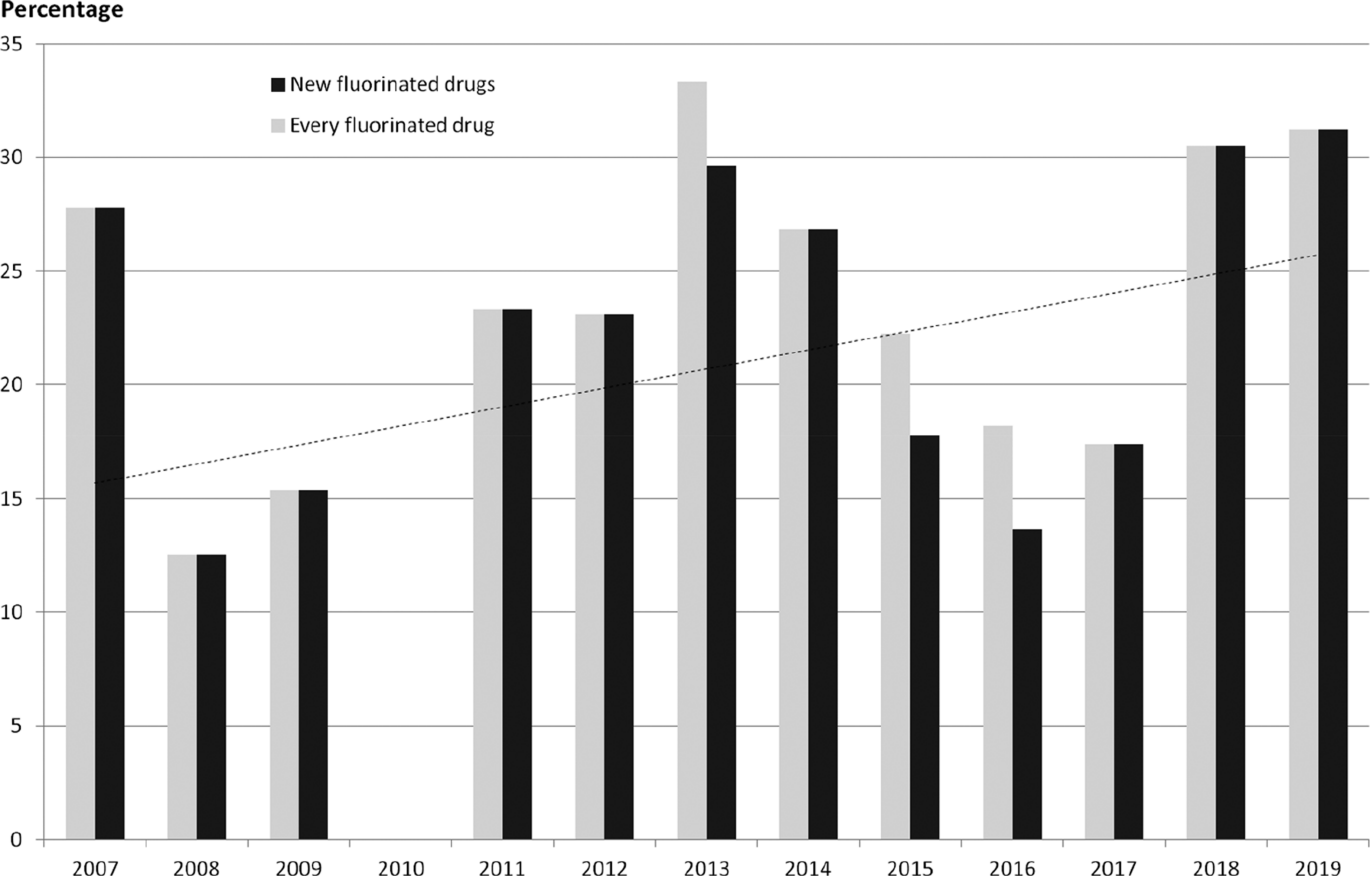
Yearly Percentages of Fluorine-Containing Drug Molecules within Drugs Approved by the FDA in That Year^,^ Biologics and inorganics were included. The dashed line is the trend line of new fluorinated drugs.

To uncover more recent developments, drugs approved by the FDA in 2007–2019 were investigated. In this case, biologics and inorganics were not excluded, and after performing the calculations as above, the percentages shown on Scheme 36 were obtained. Drugs with at least one fluorine-containing active pharmaceutical ingredient (API) were categorized as “Every fluorinated drug”. Within these, “New fluorinated drugs” contained at least one, previously not approved fluorinated API. Most fluorine-containing drugs fell into this category. The exceptions were Breo Ellipta (2013, fluorinated API: fluticasone furoate), Genvoya (2015, fluorinated APIs: elvitegravir and emtricitabine), Lonsurf (2015, fluorinated API: trifluridine), and Epclusa (2016, fluorinated API: sofosbuvir). Similarly to the time period 2002–2006 (Scheme 35), most percentages are in the 15–30% region. The trend line indicates that the prevalence of fluorine-containing drugs is still increasing. Taking into account the recent advance of biologics within approved drugs, this is remarkable.^87^ Interestingly, 2010 was the first year since 1980 when no fluorinated drugs were approved.

**Scheme 37:**
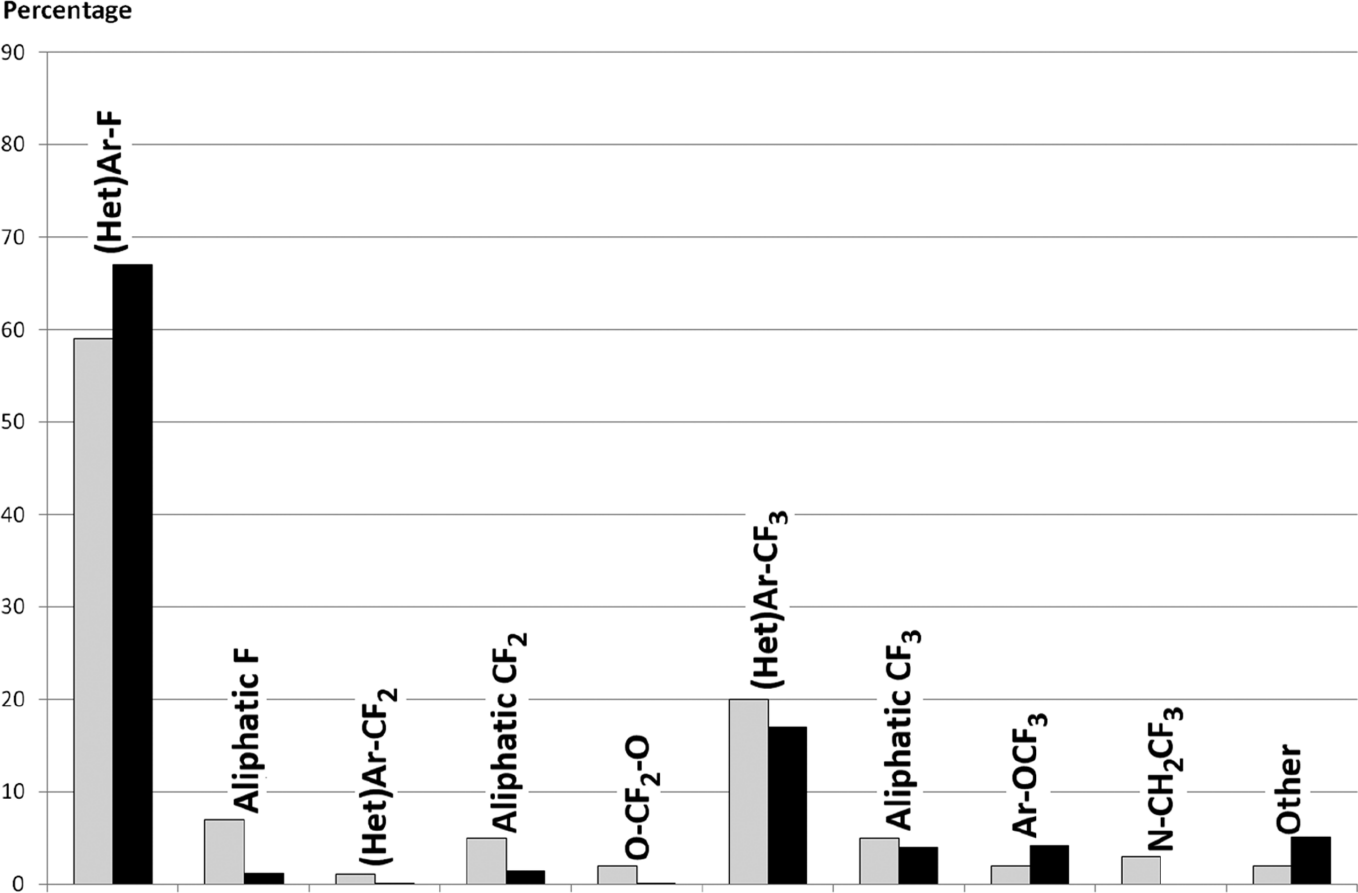
Prevalence of Different Fluorine-Containing Moieties within New Fluorinated APIs of Drugs Approved by the FDA between 2007 and 2019 (Grey Columns) and within Commercially Available Fluorinated Building Blocks of TCI (Black Columns)

For new fluorinated APIs within drugs approved by the FDA in 2007–2019, the prevalence of different fluorine-containing moieties is shown in Scheme 37. (Note: since there were exactly 100 new fluorinated APIs, this percentage is equal to the number of APIs containing the group in question.) The most common motifs are (hetero)aryl fluoride (59%) and the trifluoromethyl group bound to the (hetero)aryl group (20%). Aliphatic moieties like fluorides (7%), CF_2_ groups (5%), and CF_3_ groups (5%) are much less common. 2,2,2-Trifluoroethylamino motifs (3%), aryl trifluoromethyl ethers (2%), difluoromethylene ethers (2%), and difluoromethylated arenes (1%) are rare. The aryl difluoromethyl ether moiety of roflumilast and the SF_6_ component in the ultrasound-enhancing agent Lumason were classified as “Other” (2%). Note that the sum of the above percentages is higher than 100% since molecules can contain more than one kind of fluorinated group.

Because fluorination reactions are still challenging,^82,83^ most fluorine-containing drugs are synthesized using commercially available fluorinated building blocks.^17,84−87^ As a result, the occurrence of different fluorine-containing moieties is connected with both the pharmaceutical usefulness of different fluorinated groups and the availability of appropriate fluorinated compounds (which depends on the limits and possibilities of current fluorine incorporation methods). To gain some insight into the latter, the TCI brochure “Fluorination Reagents, Fluorinated Building Blocks” was analyzed.^88^ Under “Fluorinated Building Blocks”, this brochure listed 1815 compounds. After exclusion of highly fluorinated ethers like isoflurane (catalogue code: C2485) which have low synthetic usefulness, the remaining 1803 molecules were grouped according to their fluorine-containing moieties. As shown in Scheme 26, the two most common motifs are the same as in the APIs, and even their frequencies are roughly the same [(Het)Ar–F: 67% of building blocks and 59% of APIs, (Het)Ar–CF_3_: 17% of building blocks and 20% of APIs]. This clearly demonstrates that the most common fluorinated motifs in APIs are the most accessible ones. However, less frequent fluorinated moieties show that other factors are present too. Although the occurrence of aliphatic CF_3_ moieties is similar within the two compound groups (4% of building blocks and 5% of APIs), alkyl fluoride and aliphatic CF_2_ moieties are significantly more represented in APIs than in the TCI brochure (aliphatic F: 1% of building blocks and 7% of APIs, aliphatic CF_2_: 1% in building blocks and 5% in APIs). On the other hand, aryl trifluoromethyl ethers are more represented among building blocks (4%) than in APIs (2%). (Het)Ar–CF_2_ moieties are quite rare (only 1 API and 2 building blocks contained it), so the difference between their frequencies (0% of building blocks and 1% of APIs) is not meaningful. The case of difluoromethylene ethers is special: this moiety is present only in two drugs, lumacaftor and tezacaftor, which have high structural similarity, have the same indication, and were developed by the same company, and their approvals were only 3 years apart. So, the higher prevalence of the O–CF_2_–O moiety among APIs (2 compounds, 2%) compared to building blocks (2 compounds, 0%) is not relevant. 2,2,2-Trifluoroethylamino motifs are another special case: since this group is a unique, very weakly basic amine (see Section 3.1.3) and an amide isostere (see Section 3.1.4), we can explain its higher occurrence within APIs (3 compounds, 3%) compared to building blocks (0 compounds, 0%). Finally, the higher percentage of other motifs in building blocks (5%) compared to APIs (2%) is mostly caused by perfluorinated building blocks (perfluorinated groups with at least two carbons are completely absent in the investigated APIs).

There is more evidence that incorporation of (Het)Ar–F moieties is relatively easy: on average, there are 1.49 (Het)Ar–F moieties within APIs containing them. To be more precise, within the 59 (Het)Ar–F-containing APIs, 64.4% contain only one such moiety; 25.4% contain two; and 10.2% contain three or more (five is the record in pibrentasvir).

**Scheme 38:**
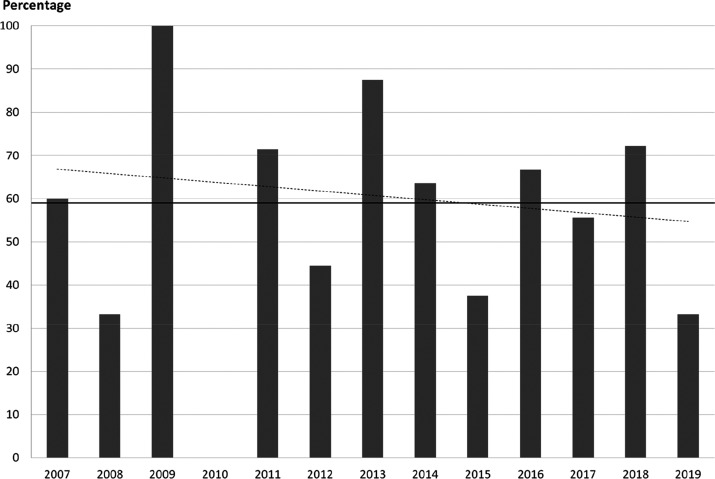
Ratio of (Het)Ar–F Containing APIs as a Function of Time^,^ The thick black line shows the average value for the 2007–2019 period. The dashed line is the trend line.

To see trends in the usage of different fluorine-containing moieties within new fluorinated APIs, it is worth taking a look at the yearly percentages of these motifs. For (hetero)aryl fluorides, ratios are shown in Scheme 38. Although this structural unit is still the most common, it seems that its prevalence is slowly decreasing.

**Scheme 39:**
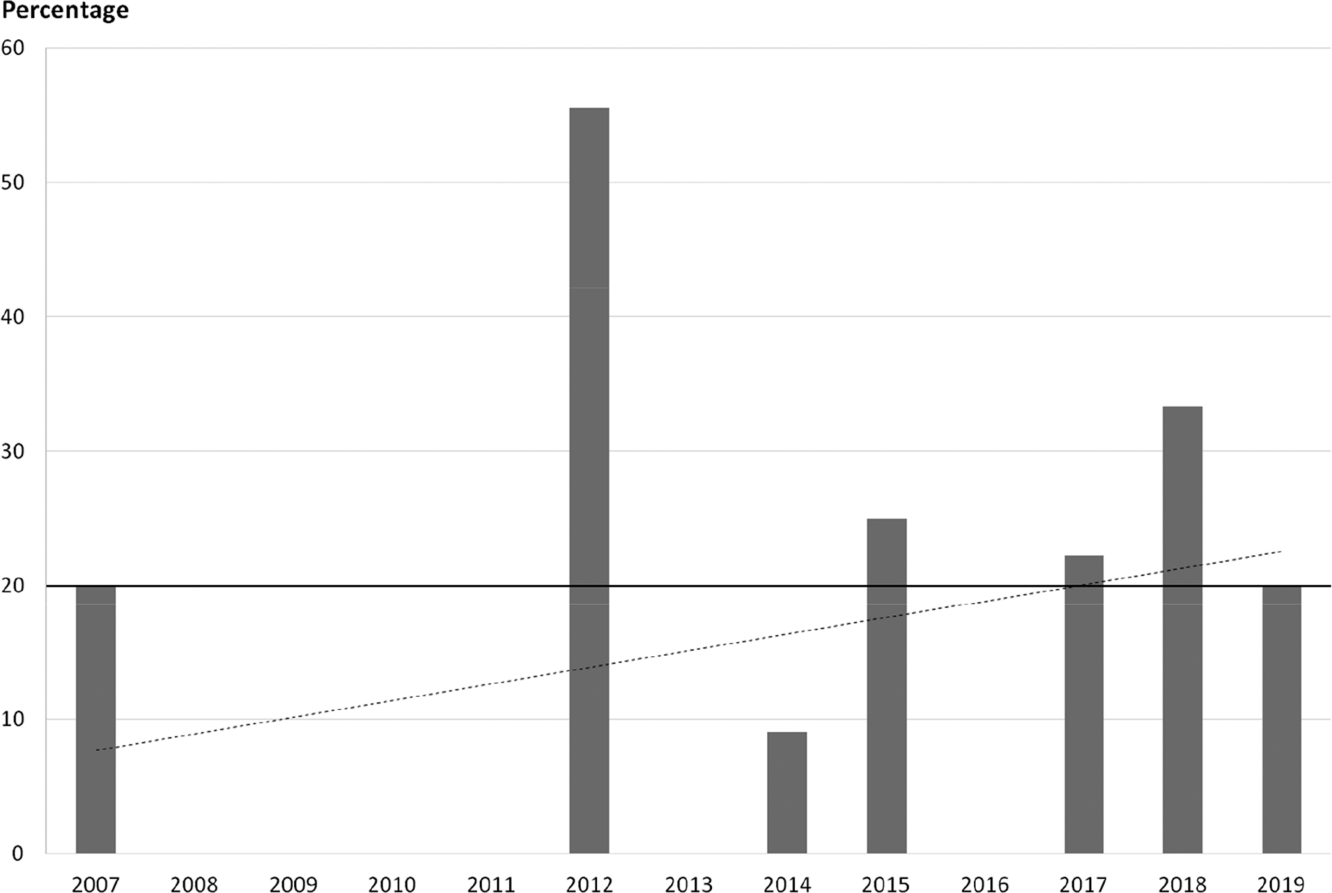
Ratio of (Het)Ar–CF_3_ Containing APIs as a Function of Time^,^ The thick black line shows the average value for the 2007–2019 period. The dashed line is the trend line.

Yearly percentages of (Het)Ar–CF_3_ motifs can be seen in Scheme 39. The trend line indicates the increasing popularity of this structural element. This is corroborated by the fact that from the 20 APIs containing this moiety 19 were approved after 2011.

Only five fluorinated APIs were approved by the FDA between 2007 and 2019 which contained aliphatic CF_3_ moieties, making statistical analysis difficult. However, 4 out of the 5 APIs in question were approved in 2015 or more recently, suggesting increased usage of this structural element. Similarly, there were only 2 APIs with the Ar-OCF_3_ moiety: sonidegib (approved in 2015) and pretomanid (approved in 2019). It is possible that these are the first signs of the emergence of trifluoromethoxylated drugs, but there are not enough members in this compound family to say this for sure.

**Scheme 40:**
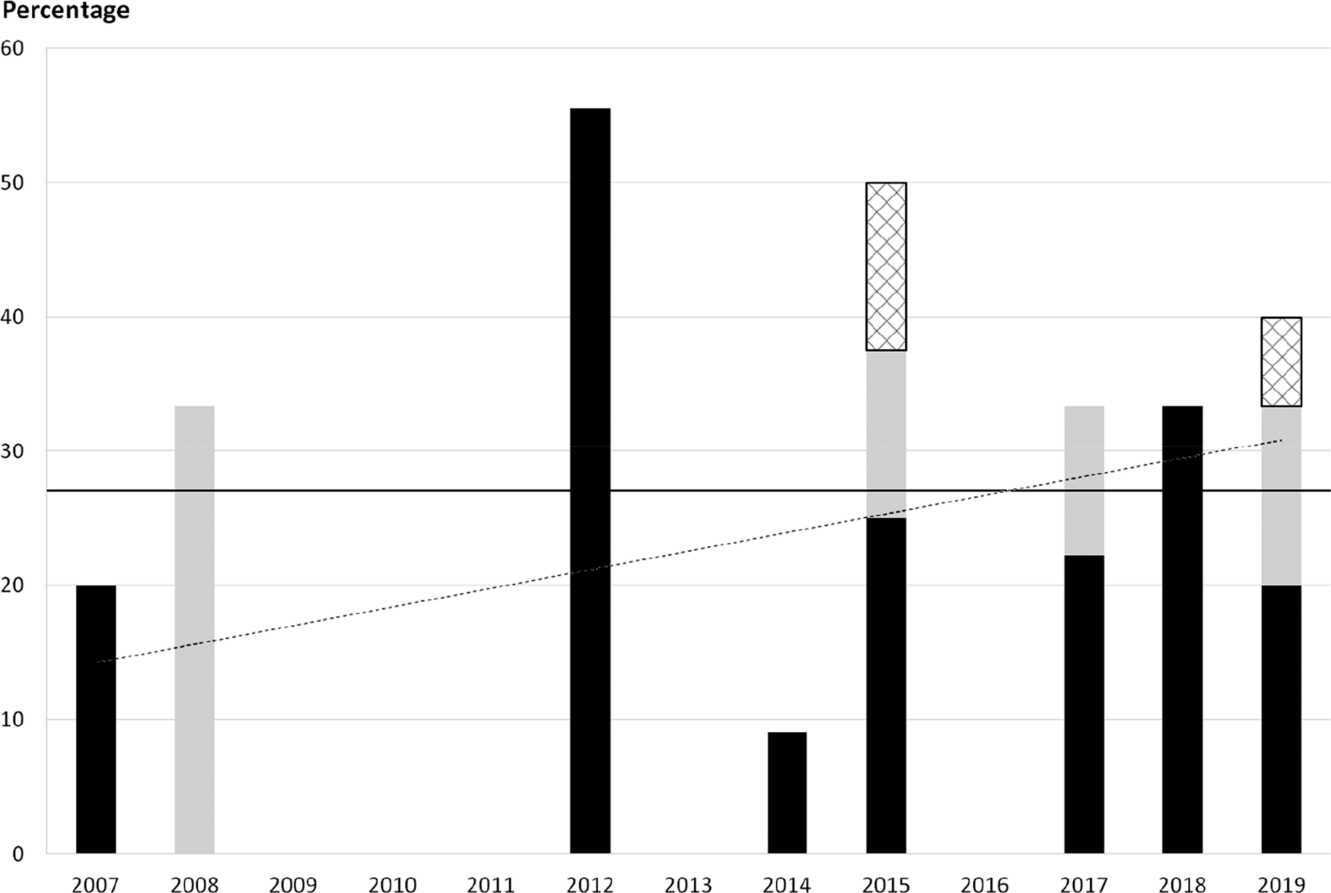
Ratios of CF_3_-Containing APIs as a Function of Time^,^^,^ Black columns: (Het)Ar–CF_3_, gray columns: aliphatic CF_3_, squared columns: Ar-OCF_3_. The thick black line shows the average value for the 2007–2019 period. The dashed line is the trend line.

In 2014, Zhu et al. predicted that the revolution in the area of trifluoromethylation, which happened in the 2000s, would increase the number of drugs with (Het)Ar–CF_3_, aliphatic CF_3_, or Ar-OCF_3_ moieties.^89^ (Drug development is a slow process, and a considerable amount of time is required for any synthetic advances to manifest in approved drugs.) To check if this prediction was correct, ratios of the 3 groups were displayed together as a function of time (Scheme 40). The trend line clearly indicates that these CF_3_-containing drugs are more and more common. The yearly numbers of these APIs (1–1 such compound was approved in 2007 and 2008 and 25 more since 2012) also support that Zhu et al. were right.

**Scheme 41:**
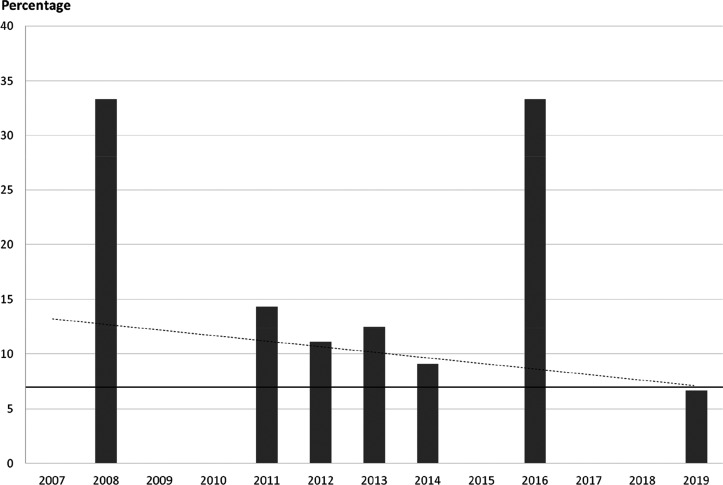
Ratio of APIs Containing Alkyl Fluoride Motifs as a Function of Time^,^ The thick black line shows the average value for the 2007–2019 period. The dashed line is the trend line.

Yearly percentages of alkyl fluoride motifs can be seen in Scheme 41. Seven APIs contained this structural element, and up to one was approved in every year. The trend line indicates slowly decreasing popularity, but the low number of drugs in this compound family decreases its reliability. Notably, however, this is the most common fluorinated motif in ^18^F-containing radiopharmaceuticals (PET tracers used in diagnostics): from the 5 such compounds approved between 2007 and 2019, 3 contain an alkyl-[^18^F] moiety, while the remaining 2 contain Ar-[^18^F] moieties. This most likely originates in the unique synthetic challenges of this compound family. First, since the half-life of ^18^F is only 110 min, it has to be introduced at a late stage of the synthesis. This necessitates the use of a quick, effective, and functional group tolerant fluorination reaction. Second, the most practical and most widely available source of this isotope is ^18^F^–^ (produced by cyclotrons as an aqueous solution, water can be removed by azeotropic evaporation), so nucleophilic fluorination methods are highly preferred. S_N_2 reactions of alkyl sulfonates with ^18^F^–^ to produce alkyl-[^18^F] moieties fulfill the above criteria effectively.^21,22,82^ For more comprehensive reviews of ^18^F-containing radiopharmaceuticals, see refs (21 and 22).

Aliphatic CF_2_ moieties are even less prevalent than alkyl fluoride motifs. Between 2007 and 2019, only 5 of the investigated APIs contained them. One such drug was approved by the FDA in 2007, 2012, and 2018 and two in 2017. The low number of data points decreases the reliability of any statistical analyses. For similar reasons, analysis of (Het)Ar–CF_2_-containing APIs (1 compound, ledipasvir, approved by FDA in 2014) and the 2 fluorinated compounds classified as “Other” (roflumilast, approved in 2011, with the Ar-OCHF_2_ moiety and the SF_6_ component in ultrasound-enhancing agent Lumason, approved in 2014) was omitted.

As mentioned previously during the discussion about Scheme 26, the case of difluoromethylene ethers is special. The two drugs where such a moiety is present are closely related (same developer company, same indication, and high structural similarity), and their approvals were only 3 years apart (lumacaftor: 2015, tezacaftor: 2018). Therefore, it is unlikely that this moiety will become significantly more frequent in the future.

**Scheme 42:**
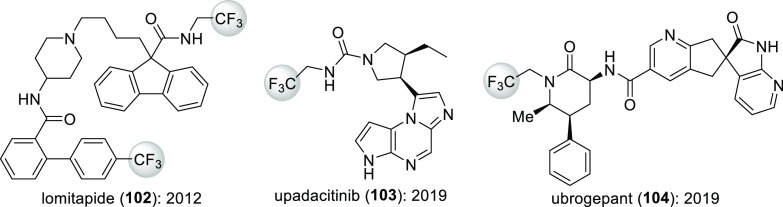
Drugs with *N*-(2,2,2-Trifluoroethyl) Groups, Approved by the FDA in the 2007–2019 Period

The situation seems to be different for APIs with 2,2,2-trifluoroethylamino motifs. As mentioned previously during the discussion about Scheme 26, this structural unit can serve as a unique weakly basic amino group or as an amide isostere. Although there were only 3 fluorinated APIs approved by the FDA in the 2007–2019 period with this moiety, these are quite different (Scheme 42). Lomitapide (**102**, Aegerion Pharmaceuticals, approved by the FDA in 2012) inhibits the microsomal triglyceride transfer protein which plays a key role in the early stages of very low-density lipoprotein (VLDL) assembly and is used to treat adult patients with homozygous familial hypercholesterolemia.^90^ Upadacitinib (**103**, AbbVie, approved by the FDA in 2019) is a selective JAK1 inhibitor which is used for the treatment of adults with rheumatoid arthritis.^17^ Finally, ubrogepant (**104**, Allergan, approved by the FDA in 2019), a CGRP receptor antagonist, is used for the acute treatment of migraines.^17^ Odanacatib (**74**, Scheme 23), a cathepsin K inhibitor which was promising for the treatment of osteoporosis but whose development was discontinued because of safety reasons, also contained a 2,2,2-trifluoroethylamino motif.^66^ Taking these into account, the fact that every drug belonging to this compound family (molecules **102**–**104**) was approved since 2012 (in fact, two-thirds of them were approved last year) strongly suggests that we are witnessing the first steps in the emergence of drugs with 2,2,2-trifluoroethylamino motifs. The current low number of such APIs causes some uncertainty, but the upcoming years will definitely show whether the above prediction was correct or not.

#### 3.3.2 Agrochemicals

Fluorination is more and more widespread among agrochemicals. Herbicides demonstrate this very well. Up to 2010, almost 25% of licensed herbicides were fluorine-containing.^79^ However, among herbicides commercialized between 2010 and 2016, the same ratio increased drastically to 75% (see details below).^20^

Together with herbicides, other classes of pesticides commercialized between 2010 and 2016 were also investigated. Within all pesticides (8 herbicides, 8 fungicides, 4 insecticides/acaricides, and 4 nematicides; 24 compounds in total), the ratio of fluorine-containing ones was 75% (6 herbicides, 7 fungicides, 3 insecticides/acaricides, and 2 nematicides). From the above data (ratio of fluorinated agrochemicals was almost 88% among fungicides, 75% among insecticides/acaricides, and 50% among nematicides), it is clear that fluorination became quite popular in every class of pesticides.^20^

## 4 Fluorine in the Environment

Fluorine is the most reactive chemical element, and since recently it was believed to not occur in nature. Minerals antozonite^39^ and villiaumite^91^ were reported as the only two exceptions in which occlusions of fluorine as gas were reported. In combination, fluorine comprises 0.059% of the earth’s crust, being the 13th element in abundance and the most abundant halogen.^23^ Fluorine plays an important role in geochemical and biogeochemical systems despite its relatively low overall abundance on the Earth and in the Cosmos.^92^ There is some confusion on the use of the terms fluorine and fluoride in the literature. In this text, the term fluorine (F) will be used to denote the element or the element in any of its forms, and the term fluoride may be taken either for a compound/material or the fluoride anion (F^–^).

Inorganic fluorides and organofluorine compounds are present in the environment due to natural and anthropogenic activities. The natural sources include the weathering and dissolution of minerals, emissions from volcanoes, forest fires, and marine aerosols.^93,94^ The anthropogenic sources include different industrial processes, like coal-fired power generation, brick making and ceramic manufacture, and aluminum production and phosphate fertilizer production, and agricultural practices, including use of phosphate fertilizer and sewage sludge application and use of fluorine-containing herbicides and pesticides, etc.^95^ Controlled fluoridation of drinking water supplies also contributes to the fluoride dispersion. All these processes result in accumulation of fluorine-containing compounds in the soil, air, and water and through the food chains also in humans.

### 4.1 Fluorine in the Lithosphere, Air, and Water

#### 4.1.1 Lithosphere

Fluorine in the lithosphere is distributed in various minerals, like fluorspar (CaF_2_), cryolite (Na_3_AlF_6_), and apatite (Ca_5_(PO_4_)_3_(OH,F,Cl)), and in groups of minerals, such as mica, hornblende, and pegmatites such as topaz and tourmaline. Fluoroapatite is considered to be the most common fluoride mineral found in soil. Other fluorides (e.g., CaF_2_, AlF_3_) and aluminosilicates [e.g., Al_2_(SiF_6_)_3_] are also reported to occur in soils. The fluorine content in soil ranges from under 100 to several mg kg^–1^ dry weight (DW).^96^ In many regions, the average F content is between 100 and 600 mg kg^–1^ DW from which between 0.05 and 0.5% is available in the form of the F^–^ ion.^97,98^

#### 4.1.2 Air

Airborne fluorine exists in gaseous and particulate forms. Its distribution and deposition are dependent upon emission strength, meteorological conditions, topography, particle size, and chemical reactivity,^99,100^ The gaseous forms include hydrogen fluoride (HF), tetrafluoromethane (CF_4_), hexafluoroethane (C_2_F_6_), and silicon tetrafluoride (SiF_4_), while particulate forms include cryolite (Na_3_AlF_6_), chiolite (Na_5_Al_3_F_14_), calcium fluoride (CaF_2_), aluminum fluoride (AlF_3_), and sodium fluoride (NaF).^101^ Gaseous HF and SiF_4_ are between 1 and 3 orders of magnitude more toxic than other common pollutants (O_3_, SO_2_, peroxyacyl nitrates, and Cl_2_).^102^ Modern fluoride-emitting industries have generally little or no environmental impact; however, periods of higher than normal emissions can still occur due to routine maintenance or failure of scrubbing equipment.^98,103,104^ The maximum observed concentrations of fluoride in ambient air were 0.16 from nonurban and 1.89 μg m^–3^ from urban locations.^105^

#### 4.1.3 Water

The concentration of fluoride in natural waters depends mainly on factors such as geology, chemistry, physical characteristic, and climate, while the pH and complexing ions, e.g., aluminum, calcium, and magnesium, may affect the speciation.^103,106,107^

Seawater dominates the global hydrological cycle, and its F^–^ concentration is typically between 1.2 and 1.5 mg L^–1^.^94,108^ Surface water in areas with low natural presence of fluoride usually contains 0.01–0.3 mg L^–1^ of F^–^.^101^ Levels above 1 mg L^–1^ of F^–^ are often observed in areas with high naturally occurring fluoride reaching up to 50 mg L^–1^ in hot springs and geysers.^101^ The highest fluoride levels ever have been recorded in the Kenyan lakes Elementaita (1640 mg L^–1^) and Nakuru (2800 mg L^–1^).^109^

Well water (groundwater) fluoride levels vary mainly in dependence on the residence time and on the fluoride content of the minerals in the rock and ores that the water passes through. The potential fluoride-rich environments with fluoride content in groundwater above 1.5 mg L^–1^ are mainly linked with the Precambrian basement areas and areas affected by recent volcanism.^110^

### 4.2 Fluorine in Drinking Water, Food, and Beverages and Dietary Fluoride Supplements and Dental Products

In humans, the predominant route of absorption of fluoride is via the gastrointestinal tract through systemic (drinking water, food and beverages, and dietary fluoride supplements) and topical sources (dental products) of fluoride delivery,^111,112^ thus knowing fluorine content (see section 4.3) of these sources is of crucial importance to avoid potential problems associated with too high intakes.

#### 4.2.1 Drinking Water

The concentration of fluoride in natural waters ranges from trace to toxic concentrations. The World Health Organization (WHO) guideline value for F^–^ concentration in drinking water was set at 1.5 mg L^–1^ in 2010, based on the guideline value set in 1984 and reaffirmed in 1993.^113^ It is interesting to note that in 1994 the WHO Expert Committee on Oral Health Status and Fluoride Use suggested that 1.0 mg L^–1^ of F^–^ should be seen as an upper limit for fluoride in drinking water even in cold climates.^114^

In some countries, fluoride is deliberately added into water supplies.^115^ The WHO-recommended value for artificially added fluoride is usually between 0.5 and 1.0 mg L^–1^.^113^ Recently, in the United States (US), the earlier recommended optimal fluoride concentration in water was reduced from 0.7–1.2 mg L^–1^ to 0.7 mg L^–1^.^116^ It has to be pointed out that only a few subjects in medicine have proved more controversial than public water fluoridation. While the US Centers for Disease Control and Prevention (CDC) claimed that water fluoridation is one of the ten greatest public health achievements in the US during the 20th century^115^—69% of the population receives fluoridated drinking water^115^—water fluoridation was rejected, stopped, or banned in many developed European^117^ and Asian countries.^118^

#### 4.2.2 Food and Beverages

The US Department of Agriculture (USDA) National Fluoride Database (compiled between 1977 and 2003) and the United Kingdom (UK) Fluoride Database (compiled between 2003 and 2015) report fluoride content in different ready-to-eat food items prepared using water with an average F^–^ concentration of 0.71 mg L^–1^ in the US and three different F^–^ concentrations in the UK Database (for simplicity, only results obtained using water with 0.05–0.13 mg L^–1^ of F^–^ are presented).^119,120^ Classification of foods into food groups in these databases is different. Thus, all analyzed items were categorized into 14 food groups. Average content and range of fluoride in these food groups are listed in Table 1.^121^

**Table 1:** Average Contents and Ranges of Fluoride in Food Groups

	USDA Database			UK Database		
	*w*_F_^**–**^ (mg kg^–1^)			*w*_F_^**–**^ (mg kg^–1^)		
	*n*	average (SD)	range	*n*	average (SD)	range
tea (*Camellia sinensis* L)	23	2.83 (1.18)	0.72–5.84	2	0.38 (0.32)	0.16–0.61
finfish and shellfish products	7	1.11 (0.87)	0.18–2.10	13	1.49 (2.98)	0.08–10.54
beverages and water	134	0.47 (0.34)	0.02–2.04	23	0.13 (0.11)	0.00–0.45
breakfast cereals	12	0.44 (0.19)	0.17–0.72	12	0.26 (0.30)	0.04–0.75
soups, sauces, and gravies	22	0.39 (0.34)	0.01–1.32	11	0.15 (0.21)	0.01–0.49
dishes	28	0.37 (0.20)	0.05–0.84	30	0.15 (0.12)	0.01–0.51
sweets & snacks	46	0.33 (0.26)	0.01–1.06	81	0.16 (0.19)	0.01–0.90
cereal products	9	0.27 (0.18)	0.06–0.51	16	0.31 (0.21)	0.04–0.57
meat and meat products	17	0.24 (0.14)	0.04–0.48	25	0.07 (0.07)	0.02–0.24
vegetables, spices, herbs	37^a^	0.17 (0.15)	0.01–0.49	23	0.08 (0.06)	0.01–0.19
milk and egg products	13^b^	0.12 (0.13)	0.01–0.35	28	0.07 (0.13)	0.01–0.59
infant food	50	0.12 (0.14)	0.00–0.67	251	0.15 (0.16)	0.00–1.20
fats and oils	6	0.09 (0.10)	0.01–0.25	8	0.04 (0.08)	0.00–0.17
fruits, nuts	19^c^	0.05 (0.03)	0.01–0.12	19	0.04 (0.04)	0.01–0.19
**all food groups**	**14**	**0.50 (1.62)**	**0.00–5.84**	**14**	**0.25 (3.04)**	**0.00–10.54**

a The highest content was not considered (1.15 mg kg^–1^ of F in commercial french fries).

b The highest content was not considered (1.12 mg kg^–1^ of F in cream substitute, powdered).

c The highest content was not considered (2.34 mg kg^–1^ of F in raisins).

The fluoride contents between and within food groups are highly variable (Table 1). Factors that can influence the level of fluoride in food include the locality in which the food is grown, the amount of fertilizer and pesticides applied, and the content of fluoride in water used for the production, processing, and preparation. Accordingly, the average content of fluoride of all food groups is about 2-fold higher in the USDA Database than in the UK Database. The food groups with the highest fluoride content are the tea group (*Camellia sinensis* L) and finfish and shellfish products. The former group might contain even much higher fluoride contents due to the uptake of fluoride by the teaplant from the soil,^122−126^ while high fluoride contents of the latter might be ascribed to the possible remains of the skeleton due to mechanical deboning. The average content of fluoride in other food groups ranges from trace amounts to about 0.5 mg kg^–1^ with relatively high maximum content in the beverage group. Milk and egg products, food and drinks for infants, fats and oils, and fruits and nuts are groups with the lowest fluoride content. The reported fluorine contents (Table 1) should be probably regarded as informative values only. While the concentration of fluoride in liquids was determined directly with a fluoride ion selective electrode (F-ISE), the other (solid) foods were prepared for the analysis by different methods, which may not ensure release of the entire fluorine.^127^ Additionally, results are not reported according to the “Guide to the Expression of Uncertainty in Measurement” (GUM). Issues related to (1) determination of fluorine in solids and expression of results of measurements according to GUM^128^ and (2) lack of fluorine-containing certified reference materials (CRMs) suitable for the analysis of food and environmental samples^129^ were recently raised.

#### 4.2.3 Salt, Milk, and Dietary Supplements

Systemic methods to deliver fluoride other than water, beverages, or food can be regarded as a choice for the consumer and include salt and milk fluoridation and fluoride-containing supplements (Table 2).

**Table 2:** Usual Content of Fluoride in Fluoride-Containing Salt, Milk, and Dietary Supplements

vehicle	fluoride compound	*w*_F_
salt	potassium fluoride, sodium fluoride	250–350 mg kg^–1^^130^
milk	sodium fluoride, disodium monofluorophosphate	2.5–5 mg L^–1^^131^
dietary supplements (tablets, drops, lozenges, or chewing gums)	sodium fluoride, acidulated phosphate fluoride, potassium fluoride, calcium fluoride	0.25–1.0 mg unit^–1^^132^

Salt fluoridation is sometimes suggested for communities with low F^–^ natural water concentration or communities having no possibility of implementing community water fluoridation.^133^ About 40–280 million people worldwide use fluoridated salt mainly in European, South American, and Central American countries.^130,134^ Milk was suggested as a relatively cost-effective method and effective vehicle for fluoride delivery in the prevention of dental caries.^131^ The balance between the caries’ preventive benefits and the risk of dental fluorosis has to be evaluated for the appropriate implementation of dietary fluoride supplements.^130^

#### 4.2.4 Dental Products

Systemic methods of fluoride delivery for the prevention of dental caries are questioned, and the use of dental products aimed for topical applications is recommended.^135,136^ Oral hygiene products aimed for topical applications and their fluoride content are listed in Table 3.

**Table 3:** Fluoride Content in Products Aimed for Topical Applications

source	*w*_F_ (mg kg^–1^)	comments
toothpaste	250–2800^137^	•typical strength of family toothpaste between 1000 and 1500 μg kg^–1^ of F^–^^138^
		•lower F^–^ content for the use in children has not been shown to be as effective in preventing caries as the 1000 μg g^–1^ formulation^137^
		•preventive effects in children observed at 1000 μg g^–1^ of F^–^ and higher—the decision on F^–^ levels for the use in children < 6 years should be balanced with the risk of fluorosis^138^
mouth rinses	230–900^139^	•not recommended for children < 6 years because of poor control of swallowing reflex
		•lower concentration for daily and higher for weekly use
gel	1000–12300^140^	•for professional use
varnish	7000–22600^141^	•for professional use

In addition to these products, bioactive ceramics and glasses containing fluoride ions releasing the active ingredients for long periods of time are used in conservative dentistry.^142^

### 4.3 Adequate Intake of Fluoride/Fluorine

Next to the European Food Safety Authority (EFSA)^143^ and Institute of Medicine (IOM),^144^ there are many health authorities worldwide that have considered the beneficial effects of fluoride on the prevention of dental caries as an appropriate indicator to set the adequate intake (AI) of fluoride from all sources (including nondietary sources) to between 0.05 and 0.07 mg day^–1^ kg^–1^ of body weight (BW) for children and adults.

The AI is based on empirical observation. Based on extensive research, Dean (1942) concluded that water fluoride concentration close to 1.0 mg L^–1^ was associated with a high degree of protection against caries and a low prevalence of the milder forms of enamel fluorosis.^145^ The first conversion from the exposure to fluoride in water to fluorine from intake from water and food was made in 1943 by McClure who estimated that **total fluorine intake** (and not **“only” fluoride intake**) in children at the age between 1 and 12 years ranges between 0.02 and 0.10 mg kg^–1^ of BW (average 0.05 mg kg^–1^ of BW).^146^ The genesis on how this intake became interpreted as a recommendation can be regarded as dubious.^147^ Note that the F^–^ ion has beneficial effects on the protection against dental caries and possible adverse effects on developing teeth and many other organs and tissues (see section 5). However, the total F intake is considered in the definition of AI. In accord with the genesis of AI, the term **fluoride** should be replaced by **fluorine**. Interchangeable use of these two terms is confusing and aggravates assessment of risks associated with fluoride/fluorine intake.

### 4.4 Daily Intake of Fluorine

Water, tea, beverages, fluoride supplements, and dental products are regarded as the main contributors to the oral intake of fluorine in humans.^121^ The contribution of inhaled airborne fluoride is, except for occupational exposure or exposure to fluoride by coal or fuel burning, negligible.^148,149^ Dermal absorption is insignificant except in cases of hydrofluoric acid burns.^150^

#### 4.4.1 Fluorine Intake in Children

Fluoride intakes in breastfed children are usually low even at high intakes of fluoride by mothers.^151^ Daily intakes of fluoride from drinks (water + beverages), foods, and toothpaste for 2- to 12-year-olds children residing in areas with the low, optimal, and high concentrations of F^–^ in drinking water were estimated based on research reported over the past decade (Table 4).^152−161^

**Table 4:** Daily Intake of Fluoride for 2- to 12-Year-Old Children Residing in Areas with Low, Optimal, and High Concentrations of F^–^ in Drinking Water

intake (mg day^–1^ kg^–1^ BW)				
	drinks	food	toothpaste	total
low fluoride water, *C*_F_^–^ < 0.15 mg L^–1^				
average	0.007	0.022	0.025	0.054
SD	0.004	0.008	0.016	0.019
min	0.003	0.009	0.012	0.023
max	0.013	0.028	0.055	0.062
rel. cont. %	14	40	46	100
optimal fluoride water, *C*_F_^**–**^ = 0.47–1.2 mg L^–1^				
average	0.020	0.014	0.025	0.059
SD	0.005	0.008	0.012	0.015
min	0.015	0.005	0.010	0.015
max	0.030	0.025	0.046	0.064
rel. cont. %	34	24	42	100
high fluoride water, *C*_F_^**–**^ > 2.0 mg L^–1^				
average	0.122	0.166	0.017	0.305
SD	0.086	0.095	0.006	0.128
min	0.021	0.030	0.010	0.061
max	0.274	0.268	0.022	0.385
rel. cont. %	40	54	6	100

In general, the daily intake of fluorine increases with increasing concentration of fluoride in drinking water (Table 4). Wide variations in the intakes in areas with comparable concentration of fluoride in drinking water are mainly due to different methodological approaches to collecting the data. The average intake of fluoride in children residing in areas with low and optimal concentrations of F^–^ in water exceeds the AI for 8% and 18%, respectively. In these areas, the intake of F^–^ with toothpaste accounts for almost half of the total daily intake. In high fluoride areas, drinks and food represent the major sources of fluoride intake. The intake might be in average up to 6-fold higher than the AI. The estimated intakes in children are high enough to pose a risk for development of dental fluorosis and other adverse effects caused by excessive fluoride intake.

#### 4.4.2 Fluorine Intake in Adults

There is a critical lack of recent studies on the intake of fluorine with diet in adults. The presented estimates are based on the average total intakes of fluorine with total diet (food, water, and beverages) reported before 2007.

The average daily fluorine intake in nonfluorinated areas ranges between 0.86 and 1.5 mg (average of 0.95 mg) (equivalent to 0.012–0.021 (average 0.014) mg kg^–1^ of BW for a 70 kg man).^162−166^ The average daily intake of fluorine in fluoridated areas is almost 2-fold higher, being 0.99–2.8 (average 1.8) mg, equivalent to 0.014–0.040 (average 0.026) mg kg^–1^ of BW for a 70 kg man.^162,164,167,168^

The daily intakes of fluoride can also be significantly higher. Daily consumption of 1 L of tea can contribute between 0.3 and 8.8 mg of F^–^ (equivalent to 0.004–0.126 mg kg^–1^ of BW for a 70 kg man).^121^ Some exotic leafy superfoods were suggested to contribute up to 1.25 mg of F^–^ (equivalent to 0.018 mg kg^–1^ of BW for a 70 kg man) to the total daily intake.^129^ Based on the estimate for the consumption of salt in EU, the intake of fluoride from salt can range between 2–4.2 mg day^–1^ (equivalent to 0.029–0.060 mg kg^–1^ of BW for a 70 kg man).^169^ The intake can be further increased by consumption of fluoride-containing supplements other than fluoridated salt and the use of dental products containing fluoride.

## 5 Metabolism of Fluorine-Containing Drugs

The incorporation of fluorine into biologically active organic molecules is often stated to increase their metabolic stability,^12,57^ usually based on the premise that the C–F bond is much more resistant than the C–H bond to oxidation by cytochrome P450 enzymes, which generally carry out the first metabolic transformations. To date, this has been exploited in the development of many pharmaceuticals such as ezetimibe,^170^ an oral cholesterol absorption inhibitor, and celecoxib, a COX-2 inhibitor,^171^ both of which are covered in section 3.1.1.

### 5.1 Metabolic Differences between Fluorinated and Nonfluorinated Compounds

Despite these examples, the generalized statement that the introduction of fluorine in a biologically active organic molecule improves the metabolic stability can be somewhat misleading. In fact, there are many reported examples in which the introduction of fluorine in the molecule does *not* improve its metabolic stability. On the contrary, there are many reports in which the presence of fluorine increases the susceptibility of a molecule toward metabolism in the body. For example, there is evidence that a fluorine substituent *ortho* to a phenol group increases its reactivity toward methylation and glucuronidation reactions in vivo.^172−174^ Clearly, as demonstrated in the examples of ezetimibe and celocoxib, the introduction of fluorine into strategic positions of an organic molecule can favorably modify the metabolic profile, but the simple fact of incorporating a fluorine atom does not necessarily mean that the pharmacological and pharmacokinetic characteristics of the molecule will be improved.

**Scheme 43:**
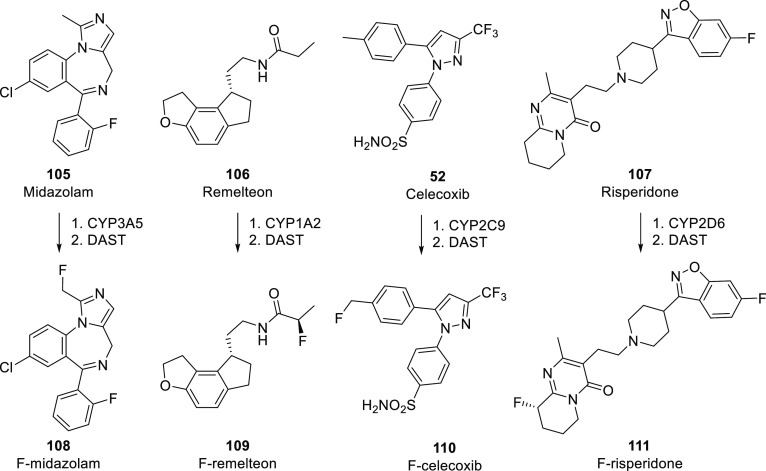
Synthesis of Fluorinated Analogues of Drugs by a Sequential Process of CYP450-Mediated Oxidation and DAST-Mediated Fluorinatioon

In 2016, Obach et al. reported interesting findings exploring the effects of replacing a metabolically labile alkyl C–H bond with a C–F bond in several commercial pharmaceuticals.^175^ First, the authors incubated midazolam, ramelteon, celecoxib, and risperidone with P450 enzymes in order to obtain the hydroxyl metabolite, and these were then subjected to deoxyfluorination reactions with DAST which produced the corresponding fluorinated derivatives (Scheme 43). In this way, the authors successfully introduced a fluorine atom at the position most susceptible to metabolism by P450 enzymes, thereby hoping to block oxidation in that position and produce a fluorinated version of the drug with a longer biological half-life in vivo. When the fluorinated analogues were reincubated with the same P450 enzymes used to introduce the hydroxyl group during the original preparation, only F-celecoxib **110** and F-risperidone **111** were found to be more stable compared to their nonfluorinated counterparts. The introduction of fluorine had no effect on the half-lives of F-midazolam **108** and F-remelteon **109** in this assay. On the other hand, when incubated with human liver microsomes, the results were rather different: F-midazolam was slightly more resistant to metabolism than imidazole; however, the higher stability exhibited by F-risperidone toward CYP2D6 was considerably reduced, and both F-celecoxib and F-ramelteon were metabolized *faster* than their nonfluorinated analogues by human liver microsomes.

From this study, the authors deduced that this strategy works best on substrates that are only metabolized by P450 enzymes in one position rather than those that are hydroxylated at several sites. Furthermore, the impact of fluorine substitution at P450 hydroxylation sites on metabolic stability is somewhat unpredictable and could also depend on other factors, such as lipophilicity. Incidentally, the fact that “fluorine increases the lipophilicity of organic compounds” is another generalized yet misleading statement. In fact, the mono- or trifluorination of saturated alkyl chains often results in a *less* lipophilic molecule due to the strong electron-withdrawing capability of fluorine.^12,176^ This is also a function of fluorination pattern—vicinal difluorides on an alkyl chain are more polar than geminal difluorides due to the gauche effect favoring a conformation in which the C–F dipoles are aligned.^177,178^

**Scheme 44:**
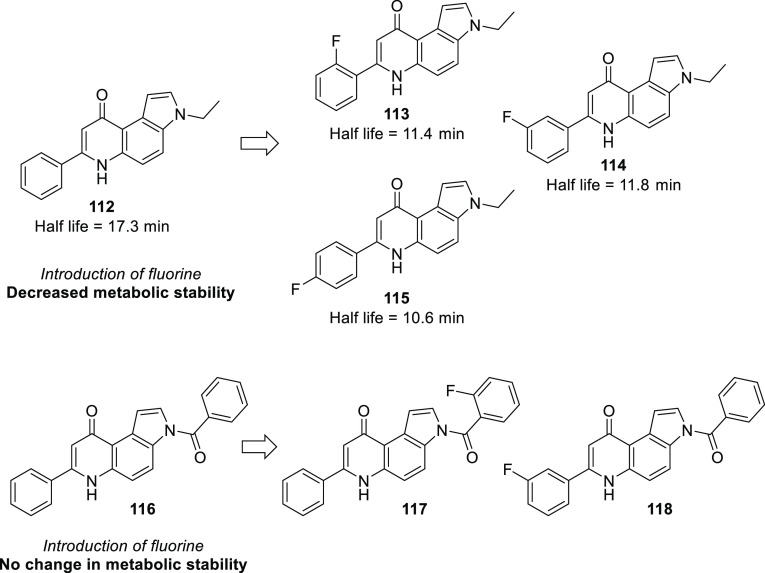
Fluorinated Derivatives of Phenylpyrroloquinolinones Shown to Have Antitubulin Activity and the Effect of the Fluorine Substituent on Metabolic Stability Against HLM

In a later study by Ferlin et al. dealing with the synthesis and biological evaluation of phenylpyrroloquinolinones, a class of compounds shown to have potent antiproliferative activity, four fluorinated derivatives were synthesized and compared to the parent nonfluorinated compounds (Scheme 44).^179^ The authors found that, although active, the fluorinated derivatives were in fact not more metabolically stable than the parent molecules. On the contrary, compounds **113**–**115** were found to have shorter half-lives than **112** when incubated with human liver microsomes and NADPH. Therefore, the authors suggested that the phenyl ring in the 7-position of **117** was not a metabolic hotspot for oxidation by CYP and that the introduction of fluorine on this ring instead caused “metabolic switching”, making the resulting compound more metabolically labile. Similarly, fluorinating the benzoyl group in **116**, which is fairly stable against NADPH-dependent oxidative metabolism but is susceptible to hydrolysis by human liver microsomes, had no effect on the stability of the compound against hydrolysis of the amide group.

### 5.2 Loss of Fluoride and the Formation of Toxic Metabolites

#### 5.2.1 Via Conjugation and Enzymatic Transformations

**Scheme 45:**
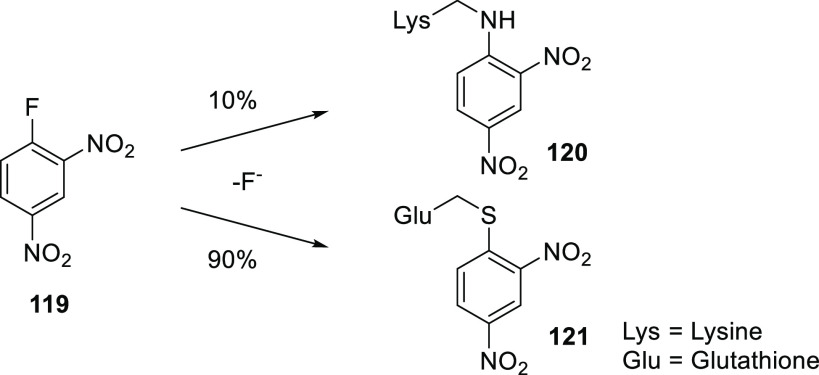
Metabolism of Dinitrofluorobenzene: Its Conjugation with Glutathione and Concomitant Loss of Fluoride

As well as studying the beneficial effects brought about by the introduction of fluorine, one should also consider the potential adverse effects that could arise, an aspect that is often overlooked. In many cases, the metabolism of fluorine-containing drugs, so-called *organic fluorine*, results in the loss of fluoride or hydrogen fluoride, or *inorganic fluorine*, from the molecule. Dinitrofluorobenzene **119** readily reacts with lysine residues in proteins and enzymes to form the dinitrophenyl hapten, producing an immune response.^180^ This accounts for around 10% of the dose in vivo, and the remaining material is conjugated to glutathione and excreted without complications (Scheme 45).^181^

Not only does this give rise to potential toxicity from the free fluoride anion, as discussed in section 4 of this review, but also other metabolites formed as a direct result of fluoride’s potential as a leaving group can also produce secondary effects. This has been exploited effectively in the development of certain *suicide inhibitors* such as eflornithine (trade name Ornidyl), which is used to treat African trypanosomiasis (sleeping sickness).^182^

**Scheme 46:**
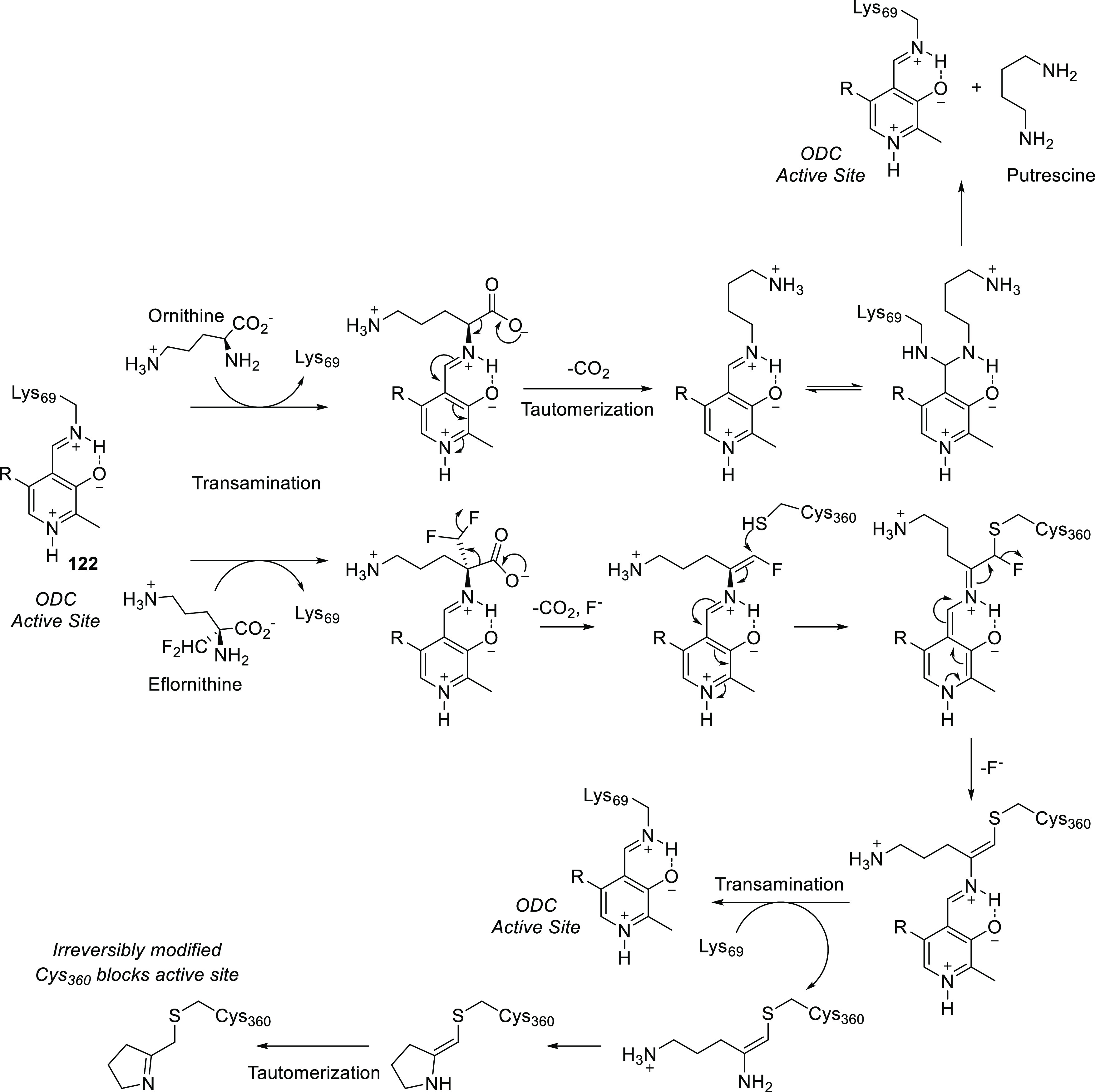
Mechanism of Action of Eflornithine

Eflornithine, a difluoromethylated analogue of the natural substrate ornithine, is a potent inhibitor of ornithine decarboxylase (ODC), the enzyme responsible for the synthesis of putrescine from ornithine and representing the first step in the synthesis of polyamines. In this way, eflornithine is accepted as a substrate into the active site of the enzyme but behaves quite differently from the natural substrate (Scheme 46).^183^ While ornithine loses a molecule of carbon dioxide to reform the starting imine, which is able to undergo a second transamination event with the nearby lysine residue to release the final product putrescine, eflornithine instead loses a fluoride ion. The resulting vinyl fluoride is then subject to nucleophilic attack by a cysteine residue located near the active site, prompting the loss of a second fluoride ion and covalently and irreversibly binding eflornithine to the enzyme active site. From there, transamination can take place to reform the starting imine to be ready for the next reaction; however, the active site remains blocked by the final 5-membered ring that remains bound to the cysteine residue within the active site (Scheme 46).^184^

**Scheme 47:**
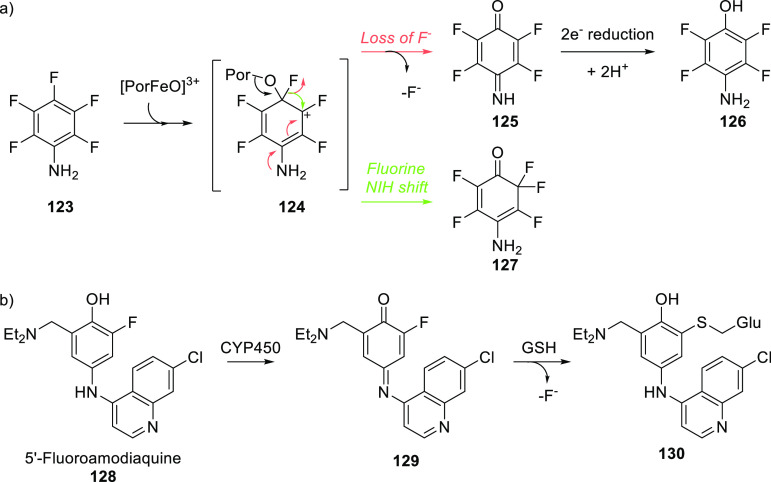
Loss of Fluoride through the Action of CYP450 Enzymes

Fluoride can also be lost as the result of enzymatic transformations, the most typical being through the action of cytochrome P450 enzymes. Although fluorinated benzene rings are often more resistant to CYP450 oxidation, hence why fluorinated phenyl rings are a common feature of pharmaceuticals and agrochemicals, the defluorination of fluorobenzene derivatives by CYP450 enzymes does still occur and is well documented.^172,185,186^ In compounds in which the fluorine substituent lies in the *para* position relative to a heteroatom such as oxygen or nitrogen, the process generally occurs via a quinone-like intermediate following oxygenation and elimination of the geminal fluoride (Scheme 47 a, red). The antimalarial drug 5-fluoro-amodiaquine, for example, is oxidized to the quinoneimine intermediate and subsequently attacked by glutathione, resulting in the 5-glutathionyl adduct and the release of free fluoride (Scheme 47b).^172,187^ Furthermore, there is also a well-established phenomenon known as the *fluorine NIH shift* by which the fluorine atom migrates to balance the positive charge, resulting in the corresponding α-fluorocarbonyl compound **127** rather than defluorinated derivative **125** (Scheme 47 a, green).^188−190^

**Scheme 48:**
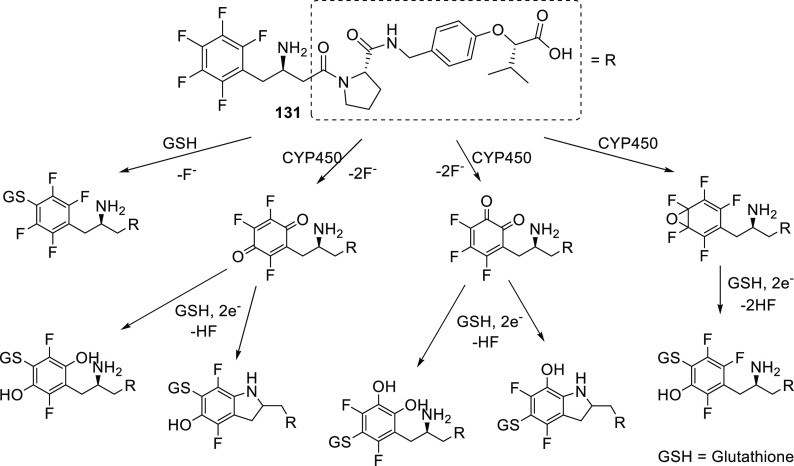
Metabolism of **131** by CYP450 to Produce Defluorinated Glutathione Conjugates

In 2005, Shang et al. demonstrated how compound **131**, originally developed as a dipeptidyl peptidase IV inhibitor, undergoes several transformations involving the terminal pentafluorophenyl ring.^191^ As discussed earlier, the introduction of fluorine substituents on a phenyl ring is a common strategy to improve metabolic stability against CYP450 oxidation. However, in this case, with five fluorine substituents withdrawing electron density, the phenyl ring is rendered sufficiently electrophilic enough to undergo direct attack by glutathione with loss of a fluoride ion (Scheme 48). In addition, as seen in the previous example, oxidation of fluorinated phenyl rings by CYP450 can still occur and is well documented, often taking place via the formation of reactive intermediates such as quinones, quinoneimines, and/or arene oxides.^192^ The authors also detected several species resulting from the conjugation of glutathione with these oxygenated intermediates, once again losing one or more fluoride ions in the process (Scheme 48).

**Scheme 49:**
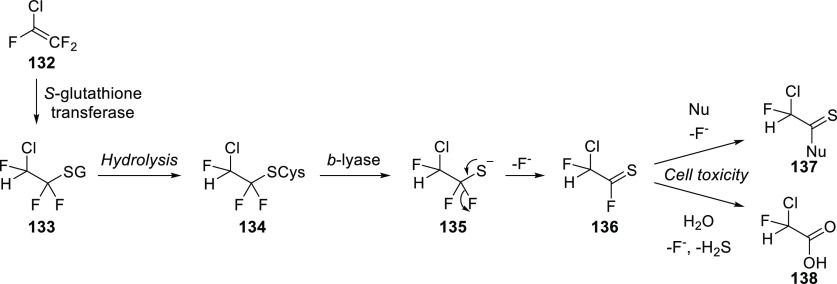
Bioactivation of Chlorotrifluoroethylene Metabolites Leading to Cell Toxicity

Chlorotrifluoroethylene (**132**), a commonly used refrigerant and monomer used to produce polychlorotrifluoroethylene (PCTFE), has been shown to undergo similar bioactivation.^193^ First, the chlorofluorocarbon is conjugated with glutathione through the action of hepatic cytosolic and microsomal glutathione *S*-transferases to form the nephrotoxic glutathione conjugate. From there, the glutathione can be hydrolyzed to give the corresponding, and also nephrotoxic, cysteine derivative **134** (Scheme 49).^194^ The authors suggest the toxicity could be due to the next steps in the metabolic fate of the cysteine conjugate, which give rise to several reactive intermediates. In addition, the authors mention that the nephrotoxicity of the released inorganic fluoride ion may play a role. The highly reactive thioacyl fluoride derivative **136**, and to a lesser extent chlorofluoroacetic acid **138**, may also give rise to cytotoxicity through the indiscriminate acylation of cellular nucleophiles.

**Scheme 50:**
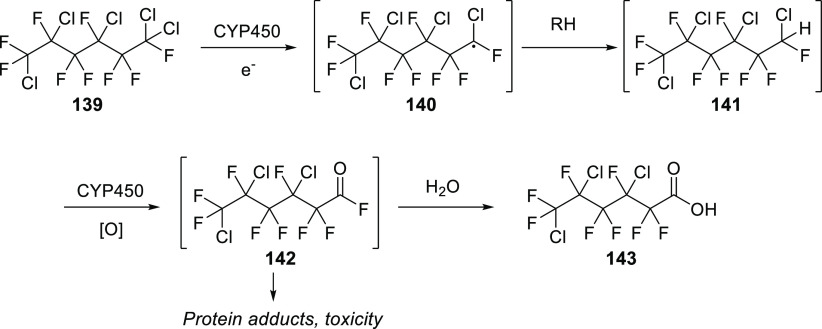
Metabolism of Aliphatic Polychlorotrifluoroethylene

The metabolism of trimers and tetramers of polychlorotrifluoroethylene has also been studied by Brashear et al. with similar conclusions.^195^ In aliphatic chains, the loss of fluorine as hydrogen fluoride generally takes place after hydroxylation of the center adjacent to the carbon–fluoride bond.^172^ In this case, the authors suggest that only those oligomers with two chlorine atoms at the terminal position could be metabolized by CYP450 enzymes, starting with a reductive dehalogenation event cleaving a C–Cl bond, consistent with the relative C–F and C–Cl bond strengths (Scheme 50). Following this, the free radical abstracts a hydrogen atom, and the resulting compound **141** can then be oxidized by CYP450 enzymes to the corresponding unstable halohydrin. From there, hydrogen chloride is lost, leaving the reactive acyl fluoride derivative **142**, another potentially harmful acylating agent, which is then hydrolyzed to the final carboxylic acid.

**Scheme 51:**
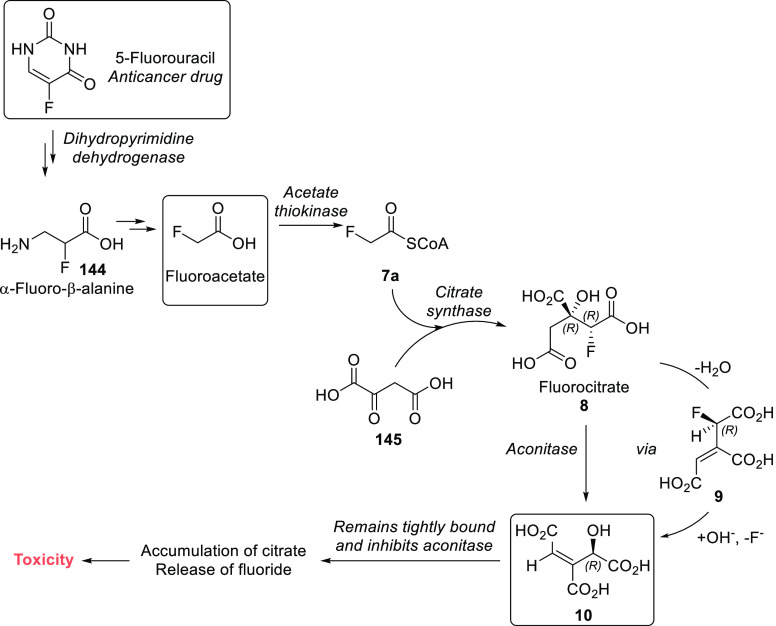
Bioactivation and Consequent Inhibition of the Citric Acid Cycle by Anticancer Drug 5-Fluorouracil

The widely used anticancer drug 5-fluorouracil has also been shown to give rise to toxic metabolites and can cause, in certain cases, serious side effects such as diarrhea, nausea, or even death in severe cases.^30,196^ This is believed to be due to its conversion into α-fluoro-β-alanine and, subsequently, into fluoroacetate and fluorocitrate which have all been show to severely limit energy production as well as exert neurotoxic effects (Scheme 51).^197−200^ The second of these is the final catabolite of fluorouracil and can enter the citric acid cycle in the same way as the native substrate acetate. In this way, fluoroacetate is transformed into fluoroacetyl CoA by the action of acetate thiokinase in the presence of ATP and Mg^2+^, which then reacts with oxaloacetate to form fluorocitrate (Scheme 51).^201^ Fluorocitrate then disrupts the citric acid cycle and causes toxicity through an accumulation of citric acid in tissues (the mechanism of this is discussed more extensively in section 2.2).

**Scheme 52:**
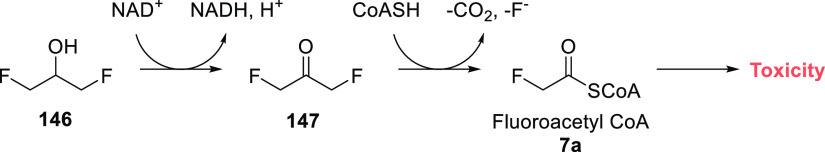
Bioactivation and Toxicity of 1,3-Difluoropropan-2-ol, the Major Ingredient in Gliftor

Fluoroacetate was used in the USA and Australia as a pesticide but was later withdrawn given the low specificity; fluoroacetate is toxic to the vast majority of mammalian species.^202^ Similarly, 1,3-difluoropropan-2-ol (**146**) was used extensively in Russia and China and was the major ingredient in the rodenticide Gliftor, which is now mostly withdrawn from use. This had a bioactivation lag of around 2 h due to the necessity for the prior formation of fluoroacetate (Scheme 52).^203^ First, 1,3-difluoropropan-2-ol undergoes NAD^+^-dependent oxidation to difluoroacetone, which is then converted to fluoroacetyl CoA **7a** and enters the citric acid cycle as seen in the previous example.

#### 5.2.2 Organophosphorus Compounds

**Figure 1:**
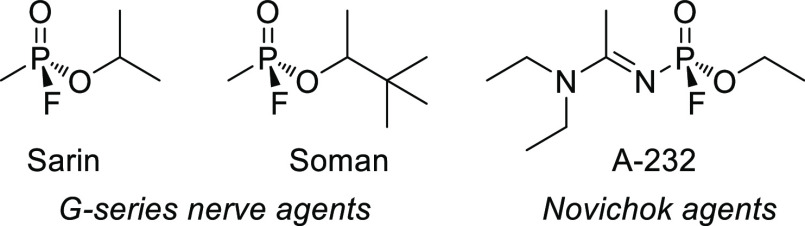
Fluorine-containing nerve agents discussed in this review.

Although the ability of fluorine to act as a leaving group has been exploited in pharmaceuticals that are beneficial for human health, it has also been applied to the development of other substances that have quite the opposite effect: nerve agents. The first nerve agent, tabun, was discovered in 1936 when the German chemist Gerhard Schrader was carrying out his research into the development of new organophosphate insecticides. Although tabun did not contain fluorine, the next compound in the series, sarin, did indeed contain a fluoride leaving group and is arguably one of the most deadly nerve agents that has been used to date (Figure 1).^204^

**Scheme 53:**
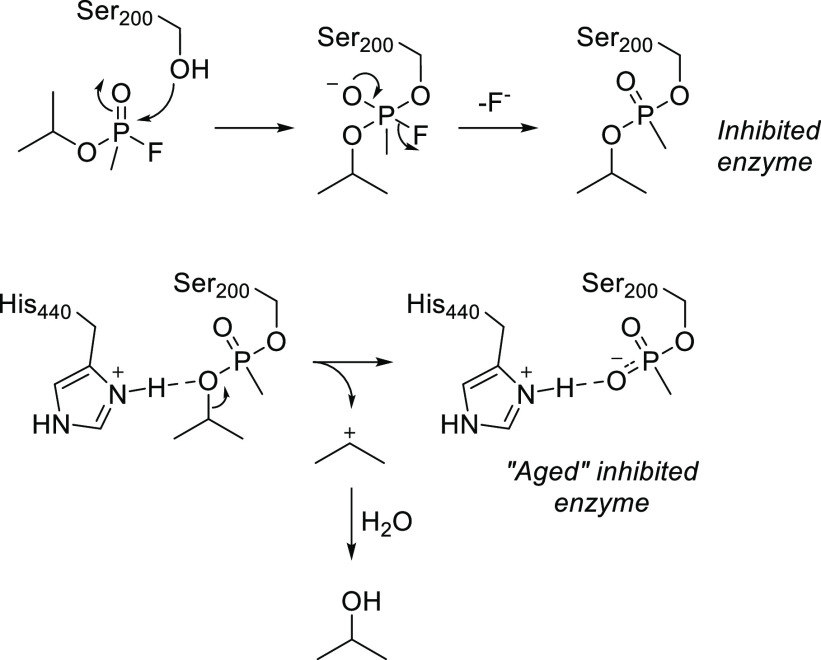
Mechanism of Action of Phosphorus-Based Nerve Agents

These agents work by irreversibly inhibiting acetylcholinesterase, thereby causing a buildup of acetylcholine and generally causing death by asphyxia due to loss of control of the muscles involved in breathing. The mechanism of action is simple and effective; the Ser-200 residue on acetylcholinesterase attacks the phosphorus center of the nerve agent and expels the fluoride leaving group. The resulting phosphonate is stable and paralyzes all enzyme activity (Scheme 53).^205^

It is worth noting that these molecules are chiral since the phosphorus is bound to four different groups, and the *S*_P_ enantiomers are generally more active than their *R*_P_ counterparts.^206,207^ However, given its comparative ease of preparation and similar effectiveness in chemical warfare, the racemate is generally used. Racemic sarin is prepared via a simple mixture of relatively nontoxic starting materials: methylphosphonyl difluoride, isopropyl alcohol, and isopropylamine.^208^

Although these compounds were developed over 50 years ago, sarin especially has seen use in modern times due to its effectiveness in war. Nerve agents were not used in World War II, but sarin is thought to have been used in the Iran–Iraq conflict in the 1980s.^209^ In fact, the largest attack using nerve agents was in this period, following an Iraqi attack on the Kurdish civilian population of Halabja, killing around 5000 people.^185^

A Japanese terrorist group known as Aum Shinrikyo used sarin in two well-documented attacks in the 1990s. One night of June 1994, they released 12 liters of sarin from the back of a truck, by means of a heater and a fan, into Matsumoto city, killing 7 and injuring approximately 500 more. Some of the victims still showed symptoms a year after the incident.^210^ A year later, the same group carried out a second attack on the Tokyo subway system, releasing sarin into trains on three separate lines.^211^ This attack was far less successful than it could have been due to the inefficient dispersion method; the sarin was contained as a liquid in plastic bags, which were pierced as the terrorists fled the scene.^212^ Even so, the attack produced 13 fatalities and affected over 5000 more, including over 50 people with severe injuries (some of whom died thereafter) and around 1000 with temporary vision problems.

Sarin has also been used in the Syria conflict throughout the 2010s in some of the most deadly attacks since the aforementioned attacks in the 1980s Iraq–Iran conflict. In August 2013, sarin was dispersed in the eastern outskirts of Damascus, causing around 1400 civilian deaths and thousands more nonfatal casualties.^213,214^ Later, in 2017, sarin was once again used in the Syrian town of Khan Shaykhun, killing over 80 people and injuring hundreds more.^215^ This attack caused U.S. President Donald Trump to retaliate and implement a strike against the base from which the attacks were believed to have been launched.

More recently, a former Russian spy, Sergei Skripal, and his daughter Yulia were found gravely ill on a park bench in Salisbury, U.K. in early 2018. It was later found that they had been poisoned with A-232, a so-called *Novichok agent* (Figure 1).^216^ The pair remained in intensive care for around a month before they were discharged.

**Figure 2:**
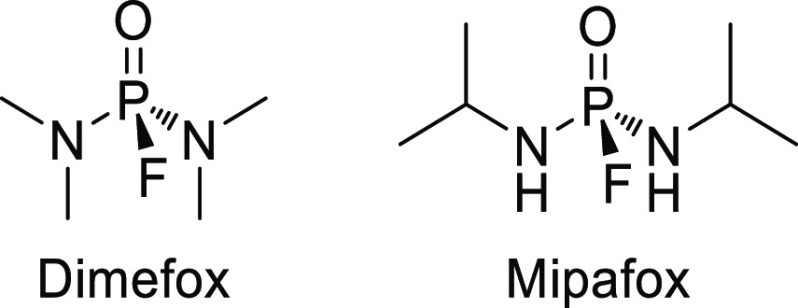
Structures of withdrawn organophosphate insecticides.

Nevertheless, one must not forget why compounds with such activity were developed in the first place, for use as insecticides. In fact, the same Gerard Schrader responsible for the discovery of tabun and sarin also developed dimefox, an effective insecticide that is now prohibited in the vast majority of developed countries including the E.U. and the U.S. (Figure 2). Another organophosphorus insecticide bearing the same fluoride leaving group, the structurally similar mipafox, was also withdrawn from use following toxicity concerns (Figure 2).^217^ However, organophosphorus acetylcholinesterase inhibitors as a compound class are still used to this day as insecticides despite their relatively high toxicity in mammals—they are thought to cause up to 200 000 accidental deaths each year,^218^ and curiously, they are a relatively common suicide method in Turkey.^219,220^

#### 5.2.3 Fluorinated Anesthetics

**Scheme 54:**
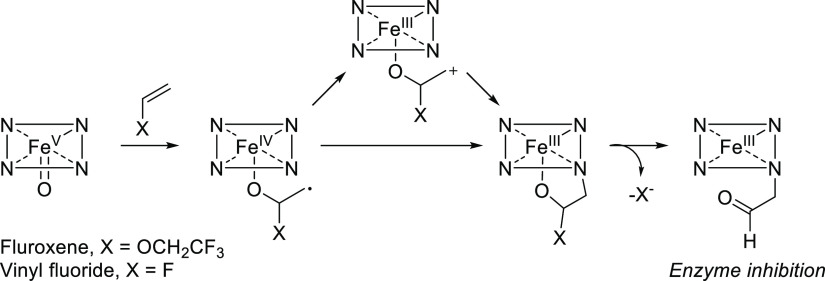
CYP450 Inhibition by Fluoroxene, An Early Anaesthetic, and Vinyl Fluoride

Since the 1950s, fluorinated compounds have been extensively used as anesthetics, starting with fluoroxene which replaced diethyl ether due to a kinetic enhancement.^221^ However, fluoroxene was quickly put into misuse since it was flammable, toxic, and pungent and had several unwanted postoperative side effects on patients. In fact, it has been shown that fluoroxene, as well as vinyl fluoride, inhibit the action of CYP450 enzymes through a suicide-based mechanism (Scheme 54).^222^ The authors provided evidence of a free-radical-based mechanism, by which the oxidation takes place on the double bond at the same carbon as the substitution (F in the case of vinyl fluoride and OCH_2_CF_3_ in the case of fluoroxene), leaving a free radical at the terminal position. This then alkylates one of the nitrogen atoms of the porphyrin ring, and the leaving group is subsequently expelled, leaving the porphyrin ring alkylated and the CYP450 inhibited.

**Scheme 55:**
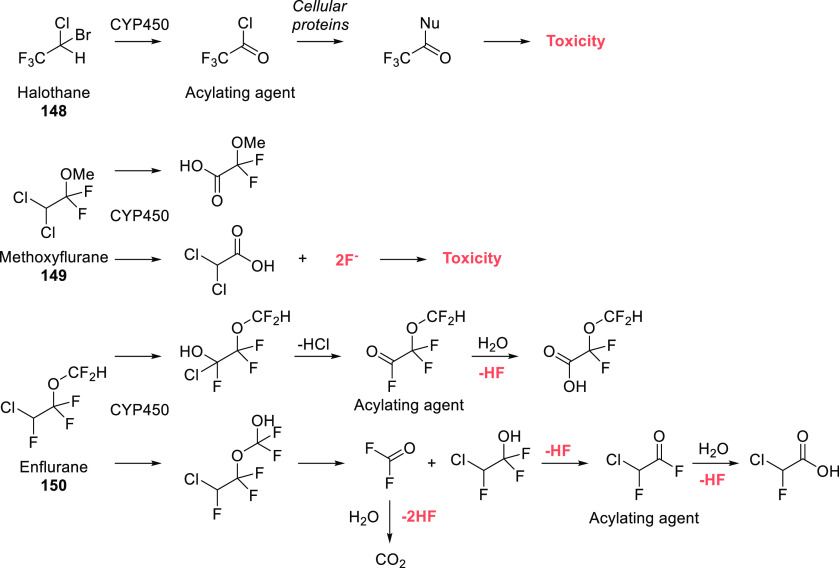
Formation of Toxic Metabolites from Fluorinated Anaesthetics

Halothane quickly took over from fluoroxene after it was found to be highly potent as an inhaled anesthetic and has relatively low toxicity compared to fluoroxene. However, it was later shown to cause to a certain degree of nephrotoxicity, due to hepatic CYP450 oxidation giving rise to the reactive trifluoroacetyl chloride which can acylate cellular proteins, as seen previously (Scheme 55).^223^ Another anesthetic, methoxyflurane, was also shown to cause a certain degree of nephrotoxicity, albeit through a different mechanism. In this case, CYP450 has been shown to metabolize methoxyflurane into two different products, neither of which are toxic individually. However, when dichloroacetic acid is formed, two molecules of fluoride are also released, and the mixture of these two compounds together is indeed nephrotoxic (Scheme 55).^224,225^ Due to this, the use of methoxyflurane was discontinued in the USA and Canada in 1999, although other places such as New Zealand, Australia, the UK, and Europe continue to use methoxyflurane in specific situations.^220^

Enflurane replaced these anesthetics in part since it seemed to be safer, although further studies showed that it produced nephrotoxicity similar to methoxyflurane (Scheme 55).^226^ This is also thought to be due to the release of fluoride ions; this is similar in other modern fluorinated anesthetics. However, the modern anesthetics are much more resistant to metabolism and as such are generally deemed much safer.^220^

### 5.3 Environmental Factors and Bioaccumulation of Fluorine-Containing Compounds

Fluorinated compounds have also caused a certain degree of environmental damage over the years since upon discovery of new compounds with positive characteristics for a certain application there has been a general hastiness to put them to use. Any toxicity studies in terms of humans and the environment have come much later once the damage had already been done. A very typical example of this is DDT, which was found to be an excellent insecticide and was extensively put to use between the 1950s and 1980s. It was later banned once its toxic effects were discovered and is now largely blamed for the decline of several species of marine life and birds of prey. Although DDT does not contain any fluorine atoms, it does contain chlorine atoms and shares similar physical chemical properties with many fluorinated compounds: highly lipophilic and a very long half-life in the environment.

In fact, many studies on the presence and geographical distribution of pharmaceuticals and agrochemicals have been published previously. Of the 61 most frequently encountered pharmaceuticals in the environment, 14% contain fluorine, and more specifically 8% correspond to fluorinated antibiotics.^34^ Perhaps unsurprisingly, the drug encountered at the highest concentrations globally is ciprofloxacin, a fluoroquinolone that is one of the most widely used antibiotics worldwide. The figures can be worrying since many of these compounds are found in water systems and in organisms at biologically relevant concentrations; in fact, many studies exist reporting how such concentrations affect various organisms.^227^ Schoenfuss et al. found that the predator avoidance behavior of larval fathead minnows is hampered upon treatment with environmental concentrations of antidepressants such as fluoxetine.^228^ The same drug also resulted in reduced growth rate for tadpoles, resulting in smaller frogs at a disadvantage for predator evasion, seeking new territories, and mating successfully.^229^ Recently, Yokoyoma described how pollutant levels of the insecticide diflubenzuron are enough to affect the embryonic development of the caddisfly, causing thorax and leg abnormalities and reducing their survival rates.^36^ These few examples are just an indicator of the effects humans are having on the environment in terms of pollutants from the pharmaceutical and agrochemical industries, but many more studies have been published with similarly concerning results.^34^

#### 5.3.1 Chlorofluorocarbons (CFCs) and Hydrofluorocarbons (HFCs)

**Scheme 56:**
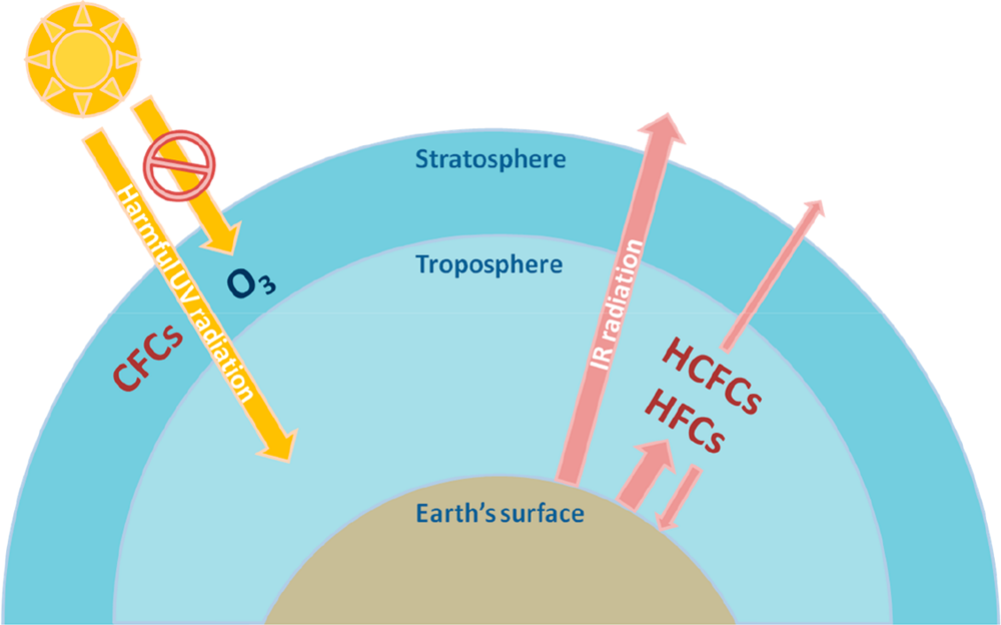
Effects of CFCs and Derivatives in the Atmosphere CFCs destroy the ozone and allow harmful UV radiation through to the Earth’s surface. HCFCs and HFCs do not reach the stratosphere and therefore do not destroy the ozone layer but absorb and re-emit IR radiation from the Earth’s surface, producing a greenhouse effect.

A well-known example of the potential problems of fluorinated compounds in the environment is the use of chlorofluorocarbons (CFCs). These were first developed in the 1930s and widely used in many applications including propellants, foam-blowing agents, refrigerants, and solvents due to their ideal properties (low toxicity, high stability, low flammability, etc.).^230^ Unfortunately, the fact that their use would lead to the destruction of the ozone layer was not predicted until the year 1974, and from then on it quickly became apparent that this was becoming a reality.^231^ However, the ozone-depleting properties of these compounds is not strictly due to their fluorine content. The actual mechanism by which CFCs destroy ozone is through chain reactions involving chlorine that can be released from the starting compounds through photolysis of the C–Cl bond. In fact, CFCs have been largely replaced with hydrochlorofluorocarbons (HCFCs) and hydrofluorocarbons (HFCs) which do not have such a disastrous effect on the ozone layer; given their shorter half-life, they mainly remain within the lower atmosphere and generally do not reach the stratosphere (Scheme 56). Incidentally, CFCs have also been found in groundwater samples, even in 2019, and have been shown to degrade to the toxic compound difluoroacetic acid.^232^

Nevertheless, the newer HFCs are also beginning to be more restricted in their use in recent years. Although they do not contribute as much to the depletion of the ozone layer, they are in fact greenhouse gases and contribute significantly to global warming through the absorption of IR radiation.^233^ Due to this, an amendment to the Montreal Protocol was passed in 2016 to begin phasing out the use of HFCs in 85% of countries by 2024.

Volatile anesthetics can be similar in structure to CFCs and therefore possess similar properties with regard to their atmospheric presence; they also contribute to the greenhouse effect and have long atmospheric lifetimes (desflurane remains in the atmosphere for approximately 14 years after being released).^234^ Significant amounts are generated as waste and released into the atmosphere from hospitals. Therefore, there have been many efforts in recent years to develop ways to capture the waste gases and recycle them due to the low metabolic rates inside the body; only <0.1% of desflurane and 3–5% of sevoflurane are metabolized, and the vast majority passes through the patient unaffected. In early studies, soda lime was explored for this purpose,^235^ but more recently zeolites, activated carbon, molecular sieves, and silica gel have all been tested for this purpose with varying levels of success.^236^

#### 5.3.2 Perfluoroalkyl Substances (PFASs)

Fluoropolymer-based materials have become some of the most important out there due to their outstanding properties that are perfect for a myriad of high-tech applications; they have valuable implications in everything from energy-related materials such as fuel-cell membranes and electrolytes in lithium-ion batteries to protective coatings and fire retardants in the aeronautical industry.^4,237^ Some of their most important properties include their thermal stability, chemical inertness, and excellent weatherability and durability. However, it is precisely these properties that have caused, and continue to cause, environmental damage with regard to certain classes of these compounds.

**Figure 3:**
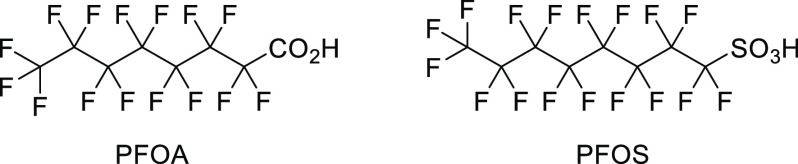
Perfluorooctanoic acid (PFOA) and perfluorooctanesulfonic acid (PFOS), the two most prevalent perfluoroalkyl substances (PFASs).

Perfluoroalkyl acids (PFAAs) are defined as aliphatic acids in which all of the hydrogen atoms attached to the carbon backbone have been replaced with fluorine, except those present in the terminal acid functional groups, and first came into use in the 1940s when their unique physicochemical properties were discovered (Figure 3).^238^ They are both lipo- and hydrophobic and have found applications as surfactants, in fire-fighting foams, and as additives in the manufacture of many materials, as well as being used in the production of PTFE, polyvinylidene fluoride, and fluoroelastomers. The most popular were the C-8 derivatives, perfluorooctanoic acid (PFOA) and perfluorooctanesulfonic acid (PFOS), although these were phased out in 2001 and replaced with other shorter-chain derivatives since they were thought to be less noxious for environmental and human health. However, the C-8 derivatives were so widely used and so environmentally stable that they remain even to this day. They are, some scientists claim, “the most persistent chemicals we are facing today”.^33^

After half a century of production and commercialization of over 3000 poly- and perfluoroalkyl substances, certain compounds now appear on the Stockholm Convention list as Persistent Organic Pollutants; due to their high chemical and thermal stability, they are deemed *practically nonbiodegradable*.^33,239^ The aqueous phase is considered a major sink for these compounds, and as such they have been detected in many water samples—including groundwater, surface water, ocean samples, and even drinking water—all across the globe, even in remote locations such as the Arctic or Antarctic Oceans.^240,241^ Despite current measures to restrict the production and use of several longer-chain PFASs, there is little to no research data available for similar compounds, such as those with a shorter perfluoroalkyl chain or other derivatives.^242^ For example, Cousins et al. point out that in the period between 2002 and November 2016 an average of just 16 peer-reviewed articles were published containing studies on each phosphorus and ether containing PFASs.^241^ Having said this, it is important to note that it is thought that branched isomers and those with perfluoroalkyl chains shorter than seven carbons are generally eliminated faster from organisms and as such are not considered to be especially bioaccumulative.^243^

Another variable is the type of predator–prey interaction being considered. For example, PFASs are thought to bioaccumulate in mammalian and aquatic predators that prey on fish more than in their avian counterparts. This could be explained through the geographical locations of these interactions; mammalian and aquatic predators tend to live in the same area and always hunt in the same area, whereas avian predators tend to venture further afield.^242^ For example, significant concentrations of PFASs have been found in polar bears,^244^ as well as in different trophic levels of fish.^245^

Clearly, we also belong in the food chain and as such are also susceptible to such bioaccumulation. Human blood samples from around the globe have also been tested for this class of organic pollutant with similar results. Furthermore, based on blood collected from retired fluorochemical workers, Olsen et al. estimated their half-lives to be relatively long: 3.5 and 4.8 years for PFOA and PFOS, respectively.^246^ Due to these long half-lives, accumulation in tissues is possible over time, a worrying prospect given the potential health concerns associated with these compounds.

Over the past decade, following the first concerns for environmental health and bioaccumulation, there have been many studies on the toxicity of PFASs. The striking lack of knowledge we had about the toxicity of these compounds can be highlighted simply by the fact that, in 2018, the European Food Safety Authority revised its decades-old safety limits of exposure to the most common C8 compounds: from 1050 nanograms per kilogram of body weight per week down to 13 ng kg^–1^ in the case of PFOS and from an astounding 10 500 ng kg^–1^ down to just 6 ng kg^–1^ per week for PFOA. Nonetheless, recent studies suggest that PFASs (more specifically the traditional PFOA and PFAS) could exert negative effects on mammary development,^247^ the immune system,^246^ thyroid,^248,249^ and liver function,^250^ as well as cause neurological problems.^251^ Studies involving the toxic effects of PFOS have been summarized in a recent review, and it seems that the main mechanism by which this compound causes biological damage is through interference with fat metabolism and oxidative stress.^252^ It has even been suggested that high levels of PFOA can induce a cohort of cancers, although the results are rarely decisive.^253−256^

Thankfully, recent data suggest that the levels of PFAS found in adult Americans’ blood have been slowly decreasing over time, likely due to the ever-increasing control imposed on the production and use of this class of polyfluorinated compounds.^257^ Moreover, shorter-chain PFASs that are emerging as viable alternatives to the older C8 derivatives have been shown to be significantly less toxic, such as perfluorohexanoic acid which has only been demonstrated to exert mild and/or reversible effects on the kidneys.^258,259^ With new measures in place and more knowledge about the dangers and problems associated with these pollutants, in the future we may be able to avoid more unnecessary damage to wildlife, ourselves, and the environment.

## 6 Fluorine-Containing Pharmaceuticals Withdrawn from the Market

Although the drug development process is long and cost-intensive, problems with efficacy, manufacturing, regulation, and severe toxicity-related side effects can result in the voluntary withdrawal of a drug from the market or a prohibition by drug regulatory agencies. Many of these adverse effects are related to efficacy and safety, two decisive factors for viability of a chemical entity.^260^

Nowadays, a large number of therapeutic agents contain strategically placed fluorine atoms, the benefits of which have been widely reported.^84−87^ In this sense and very recently, Shibata and co-workers analyzed and reported the latest contributions of organofluorine compounds to the fields of agrochemicals^86^ and pharmaceuticals^87^ and categorized them into several groups based on the chemotype of their fluoro-functional substituents. In these reviews, the authors have shown the rapid progress of synthetic organofluorine chemistry over the last two decades.

However, the involvement of fluorine in the adverse effects of certain drugs, often caused by defluorination, remains much more scarcely studied.^172^

In this regard, one of the main gaps in our knowledge for future drug development is how fluorine substitution affects the metabolism and the mechanisms leading to toxicity.^261^ Some examples of fluorine-containing pharmaceuticals withdrawn from the market are summarized here, classified according to the fluorine group present in aromatic and aliphatic systems.

### 6.1 Aromatic Substitution

#### 6.1.1 Aromatic Fluoro-Substituted Compounds

##### 6.1.1.1 Fluoroquinolone Derivatives

The quinolone family of drugs is one of the most important in medicinal chemistry. Used in both human and veterinary medicine, the quinolone nucleus is the backbone of a large group of broad-spectrum antibiotics, as well as certain derivatives proving active in chemotherapy in recent years.^262^ Quinolone derivatives are characterized by their easy synthesis through various methods, affording numerous and interesting chemical structures.^263,264^ Their structure–activity relationship has been studied extensively, and the structural requirements of the active pharmacophore in the quinolone nucleus are fairly well understood.^265,266^ In this sense, the presence of a fluorine atom in the C-6 position of the bicyclic scaffold has given rise to a very important subclass of quinolones, the fluoroquinolones, which present a wide spectrum of antibacterial activity.^267^

**Scheme 57:**
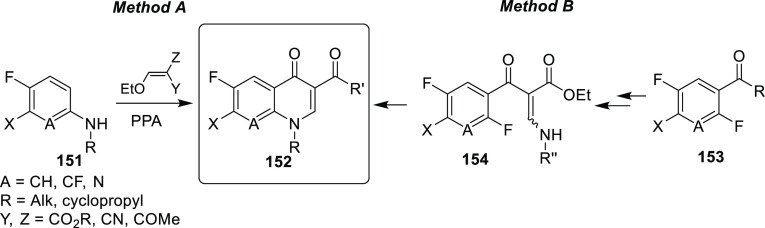
General Approaches for the Synthesis of Fluoroquinolones

For the synthesis of these fluoroquinolone derivatives, two approaches are generally used. The first of these uses fluorinated anilines, or 2-aminopyridines, **151** as the starting material (Scheme 57, *Method A*). From there, a simple condensation reaction and treatment with polyphosphoric acid (PPA) lead to fluoroquinolone derivative **152**. On the other hand, the use of fluorine-containing benzoyl derivatives **153**, followed by cyclization of intermediates **154**, comprises another method for the synthesis of fluoroquinolone derivatives (Scheme 57, *Method B*).^268^

It has been widely reported that the presence of a fluorine atom in the C-6 position enhances antibacterial activity, potentially because of improved cell penetration of the fluorinated derivatives due to their increased lipophilicity.^188^ This substitution has therefore allowed the development of new inhibitors of DNA gyrase, an essential enzyme which controls bacterial DNA replication.

Regarding the fluorine substitution, although the benefits of fluorinated drugs have been widely reported and despite the great number of them currently in use,^84,85^ the possible dangers of these drugs have not been sufficiently studied. This is possibly due to the scarce information in the literature about the involvement of the fluorine atom in biodegradation processes. Very few studies about the biotransformation of model organofluorine compounds discerning the mechanisms of their biochemical degradation or showing that the position of the fluorine substitution in the molecule has an important role in the C–F bond cleavage have been reported,^269,270^ which is often necessary for the biodegradation of fluorinated drugs.^271^

In some cases, defluorination can take place spontaneously or during drug biotransformation, depending on the electrophilicity of the molecule toward direct reaction with nucleophilic groups present in amino acids or proteins. Despite the strength of the C–F bond, defluorination can occur during drug metabolism, favoring the formation of smaller fluorinated metabolites and/or the stable fluoride ion, which is a good leaving group.^172,196^ This toxic ion and other metabolites, such as fluoroacetate, could act as enzymatic poisons, inhibiting enzyme activities and interrupting metabolic processes.

**Figure 4:**
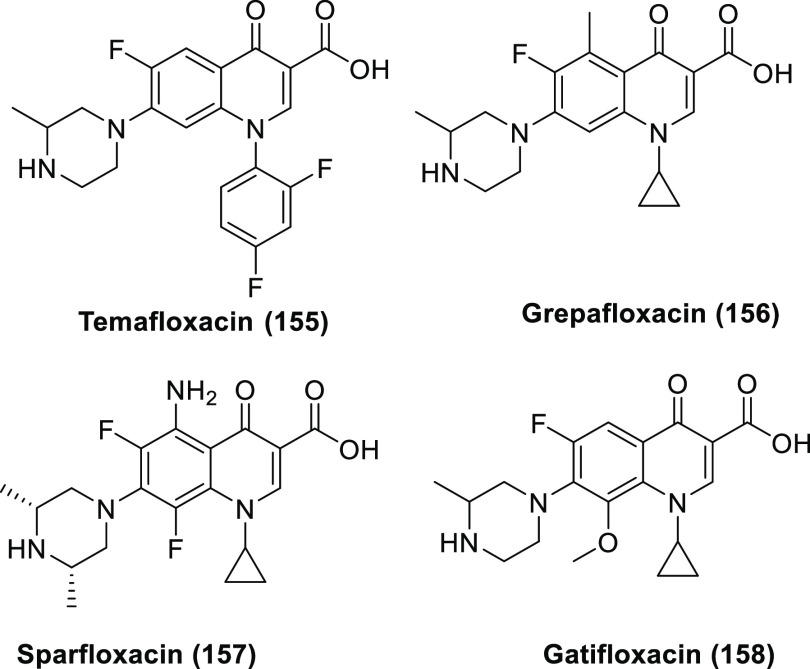
Fluoroquinolone derivatives withdrawn from the market.

The accumulation of these toxic substances obtained by the biotransformation of certain fluorinated drugs could be the cause of the adverse effects observed after their use in treating patients. For instance, adverse effects on the gastrointestinal tract and central nervous system, as well as hepatotoxicity, neurotoxicity, and phototoxicity, have all been observed in patients treated with certain fluoroquinolone derivatives,^272^ leading to their withdrawal from the market. Some such cases of fluoroquinolone derivatives are reviewed here (Figure 4).

##### 6.1.1.1.1 Temafloxacin

Temafloxacin **155** (Figure 4), a trifluorinated quinolone marketed by Abbott Laboratories as *Omniflox*, belongs to the third generation of fluoroquinolones. Temafloxacin was approved by the FDA in January of 1992 as an antibiotic agent to treat respiratory tract, urinary, genital, and skin infections related with Gram-negative pathogens, by interference with the activity of the enzymes DNA gyrase and topoisomerase IV, which are needed for the transcription and replication of bacterial DNA. However, due to serious adverse reactions reported including anaphylaxis, hemolytic anemia, renal failure, hypoglycemia, and hemolysis, *Omniflox* was withdrawn a few months later.^273^

##### 6.1.1.1.2 Grepafloxacin

Grepafloxacin **156** (Figure 4) is also a third-generation fluoroquinolone antibiotic indicated for a variety of infections including bronchitis, pneumonia, and sexually transmitted bacterial infections, marketed by Glaxo Wellcome in 1997 under the trade name *Raxar* or *Vaxar*.^274^ After severe cardiovascular events were reported,^275^ such as QT lengthening, Glaxo Wellcome announced its withdrawal from the worldwide market in 1999.

##### 6.1.1.1.3 Sparfloxacin

Sparfloxacin **157** (Figure 4) is another third-generation aminodifluoroquinolone derivative and is a synthetic broad-spectrum antimicrobial agent indicated for the treatment of various types of bacterial infections including salmonella and staphylococcus infection.^276^ Sparfloxacin (AT-4140) was patented in 1985 and approved for medical use in 1993 under the trade name *Zagam*.

Its structure presents a second fluorine atom in the C-8 position, which has been associated with phototoxicity, one of the main unwanted side effects of this drug. This group is highly photosensitive and can be eliminated under UVR exposure, losing its antibacterial activity.^277^ Other adverse reactions associated with sparfloxacin are prolongation of the QTc interval in patients with an underlying cardiac condition, insomnia, and other sleep disorders,^276^ leading to its withdrawal from the market.

##### 6.1.1.1.4 Gatifloxacin

Approved for sale and commercialized with the brand name *Tequin* by Bristol-Myers Squibb in 1999, gatifloxacin **158** (Figure 4) is a member of the fourth and latest generation of the fluoroquinolone family of antibiotics. Gatifloxacin was shown to be highly active against both penicillin-susceptible and penicillin-resistant strains of *S. pneumoniae*, the main pathogen behind the bacterial pneumonia infection.^278^ This 8-methylfluoroquinolone derivative has been used to treat lung, sinus, skin, and urinary-tract infections but has also been shown to produce dangerous changes in blood-sugar levels. Although the complete mechanism leading to hypoglycemia is not yet fully understood, experiments have shown that it can stimulate insulin release and block the ATP-sensitive potassium channels of pancreatic cells, which can both lead to hypoglycemia.^279,280^ Due to these severe side effects, Bristol-Myers Squibb announced in 2006 that gatifloxacin was to be withdrawn from the market.

##### 6.1.1.1.5 Trovafloxacin/Alatrofloxacin

**Scheme 58:**
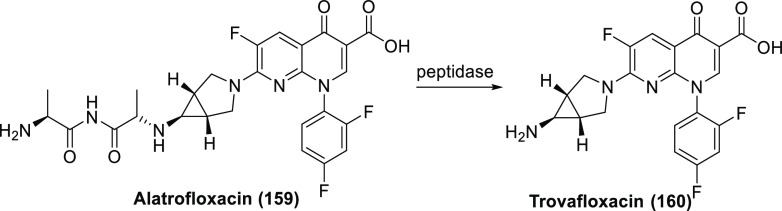
Trovafloxacin and Its Prodrug Alotrofloxacin

Trovafloxacin **160** (CP-99,219), also a fourth-generation fluoroquinolone antibiotic, is a trifluorinated derivative with a single fluorine substitution in the C-6 position of the naphthyridine ring, as well as a cyclopropyl-fused pyrrolidine at the C-7 position, which enhances its activity against Gram-positive and Gram-negative organisms (Scheme 58).^281^ While trovafloxacin is administered orally, alatrovafloxacin **159** (CP-116,517) (Scheme 58), a highly hydrolyzable prodrug of the active form **160** containing an l-alanyl-l-alanyl moiety, is used intravenously due to its better solubility.^282^

Trovafloxacin and alatrofloxacin were approved for sale in 1998 and sold as *Trovan* and *Trovan IV*, respectively, by Pfizer, for the treatment of pulmonary, surgical, intraabdominal, gynecologic, pelvic, skin, and important urinary tract infections. Although the active form, trovafloxacin, was first associated only with moderate gastrointestinal effects, headaches, and very slight phototoxicity, several cases of liver toxicity were later reported in patients treated with this drug, to the point that some even required liver transplants.^283^ Extended treatments with trovafloxacin (2 weeks) substantially increased the risk of liver toxicity, but short treatments did not necessarily avoid liver failure. In view of these data, trovafloxacin was withdrawn from the market just three years later in 2001.

##### 6.1.1.1.6 Flosequinan

**Scheme 59:**
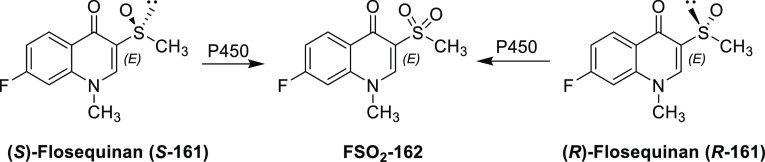
Proposed Terminal Metabolite of Flosequinan

Flosequinan is a quinolone derivative used as a potent direct-acting vasodilator, developed for the treatment of chronic heart failure (Scheme 59). Flosequinan acts on both peripheral arterial and venous vessels, improving the atrioventricular condition in patients with atrial fibrillation, although the mechanism implied is not fully understood.^284^ The metabolic pathways proposed by Kashiyama and co-workers consist of the oxidation of the sulfinyl moiety in both flosequinan stereoisomers (Scheme 59, (*S*)-**161** and (*R*)-**161**), catalyzed by the cytochrome enzyme P450, providing the flosequinan sulfone form FSO_2_**162** as the terminal metabolite.^285^

The drug was researched and developed by Boots Co. Plc. and was launched onto the market in 1992 under the name *Manoplax* in 50 and 100 mg doses. However, since no reduction in mortality was observed in heart failure patients treated with the drug, Boots decided to explore the product’s safety and efficacy on survival and mortality in congestive heart-failure patients. This study, denominated PROFILE (PROspective randomized Flosequinan Longevity Evaluation), indicated that flosequinan has adverse effects and actually showed a significant increase in mortality in patients treated with the 100 mg dose. This suggested that no benefits were being provided with the treatment, and the drug was withdrawn in 1994 by the company.

##### 6.1.1.2 Cerivastatin

**Figure 5:**
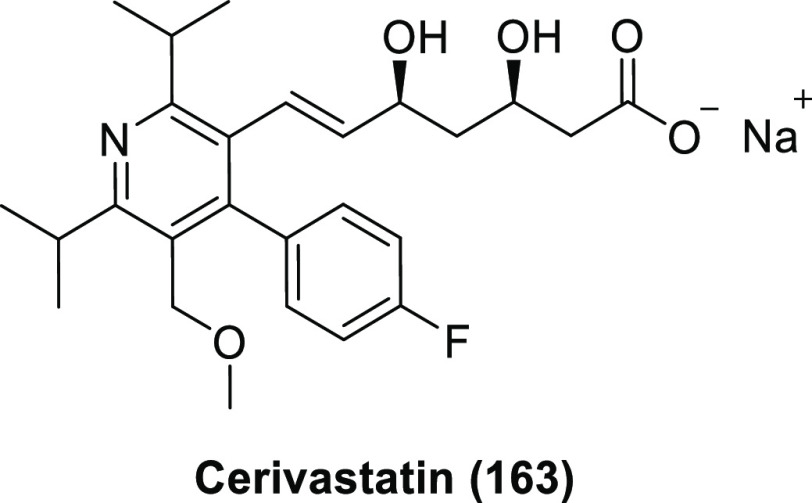
Cerivastatin structure.

Cerivastatin **163** is a synthetic and enantiomerically pure pyridine derivative used as a sodium salt (Figure 5). It is a lipid-lowering drug and is a more potent inhibitor of HMG-CoA reductase than other statin derivatives on the market, thereby blocking the synthesis of mevalonate, which is a key step in cholesterol synthesis. It was marketed by Bayer in 1997 under the brand name *Baycol* or *Lipobay* and has been used to lower cholesterol levels at microgram doses and to prevent cardiovascular diseases.^286^

Postmarketing reports about the potential breakdown of muscle tissue, and as a consequence its possible relationship with severe rhabdomyolysis cases, set off alarms about the continuity of cerivastatin in the market.

Rhabdomyolysis, a condition in which damaged skeletal muscle breaks down rapidly causing weakness, fever, dark urine, and nausea, has been associated with statins in general; however, reports of this serious syndrome were higher for cerivastatin, especially when it was administered in high doses or in combination with fibrates, another class of substances for the treatment of blood lipid disorders.^287^ Due to the risk of these serious side effects, in August 2001 Bayer decided on the voluntary withdrawal of this drug.^288^

##### 6.1.1.3 Lumiracoxib

**Scheme 60:**
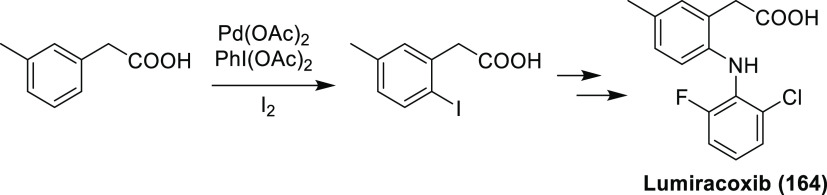
Synthesis of Lumiracoxib

Lumiracoxib **164** (Scheme 60) belongs to the class of nonsteroidal anti-inflammatory drugs (NSAIDs) and has excellent cyclooxygenase 2 (COX-2) selectivity.^289^ It was patented in 1997, approved for medical use in 2003, and was marketed by Novartis under the trade name *Prexige* for the treatment of inflammatory effects of osteoarthritis and rheumatoid arthritis.^290^

As an alternative for the original synthesis, in 2010 Yu and co-workers developed an efficient approach toward this kind of drug through a highly versatile Pd-catalyzed *ortho*-C–H iodination reaction of the phenylacetic acid group present in its structure (Scheme 60).^291^

**Scheme 61:**
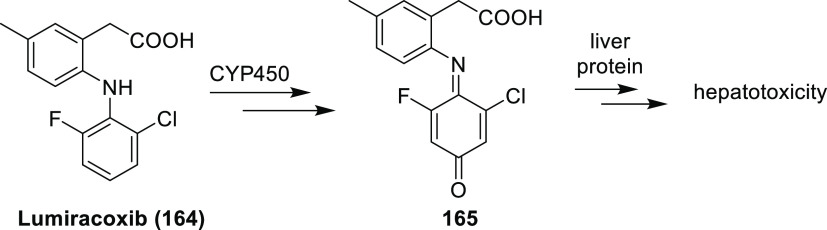
Toxic Metabolite Formation from Lumiracoxib

Despite its strong anti-inflammatory activity, lumiracoxib was withdrawn from the market due to its hepatotoxicity.^292^ Its potential for causing liver failure even led patients to require liver transplants. In this sense, subsequent studies have shown that *p*-benzoquinone imine **165** is formed in vivo after electrophilic hydroxylation by cytochrome P450 enzymes (Scheme 61). This reactive intermediate is an unselective electrophile and is then conjugated with liver proteins with concomitant fluoride ion elimination, leading to the formation of protein adducts and ultimately liver necrosis.^293^

##### 6.1.1.4 Cisapride

**Scheme 62:**
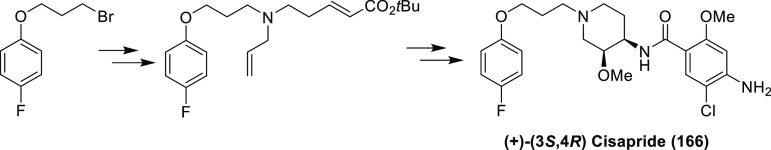
Asymmetric Synthesis of (+)-(3*S*,4*R*)-Cisapride

Discovered by Janssen Pharmaceutica in 1980, cisapride **166** (Scheme 62) acts as an agonist for 5-HT_4_ receptors and as an antagonist for 5-HT_3_ and 5-HT_2_ receptors (5-hydroxytryptamine or serotonin receptors). Cisapride was marketed under the trade name *Prepulsid* by Janssen-Ortho and *Propulsid* (United States) as a motility stimulant indicated for the treatment of hypomotility disorders of the upper gastrointestinal tract.^294^

**Scheme 63:**
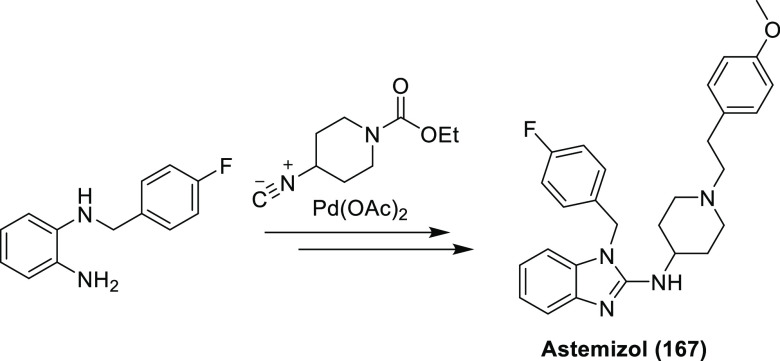
Synthesis of Astemizole

Although the commercial preparation of this drug is the racemic mixture of two enantiomers [(±)-cisapride], it is well-known that the (+)-enantiomer is more active.^295^ For this reason, despite all reported methods for its total synthesis,^296,297^ the search for an asymmetric synthesis has been an interesting objective. In this sense, Davies and co-workers developed an asymmetric synthesis of (+)-(3*S*,4*R*)-cisapride using commercially available starting materials (Scheme 63).^298^

A few years after its launch to the market, many cases of severe arrhythmia were detected, and most studies indicated that cisapride may increase the incidence of long QT syndrome, leading to serious cardiac side effects. Cisapride was therefore withdrawn in 2000 from the market worldwide.^294^

##### 6.1.1.5 Astemizole

Astemizole **167** (Scheme 63) is a second-generation histamine H1 receptor blocker and was discovered by Janssen Pharmaceutica in 1977. It was marketed in the UK in 1983 and approved for use in the USA in 1988 under the brand name *Hismanal*. As a long-acting and nonsedating antihistamine, astemizole was used for symptoms associated with seasonal allergic rhinitis and chronic idiopathic urticaria.

Ruijter and co-workers studied a novel aerobic oxidative coupling of diamines as bisnucleophiles and isocyanides using palladium catalysis to access to a wide range of guanidine-containing heterocycles.^299^ Aiming to improve on the previously reported multistage synthesis of astemizol,^300^ the authors developed a new synthetic route using this novel palladium-catalyzed oxidation reaction (Scheme 63).

However, Johnson & Johnson voluntarily withdrew astemizole from the global market in 1999 due to several cases of *torsades de pointes* (ventricular arrhythmia) which arose in patients treated with this drug. This side effect is related with the blocking of cardiac potassium channels, resulting in a prolongation of the QT interval.^301^

**Scheme 64:**
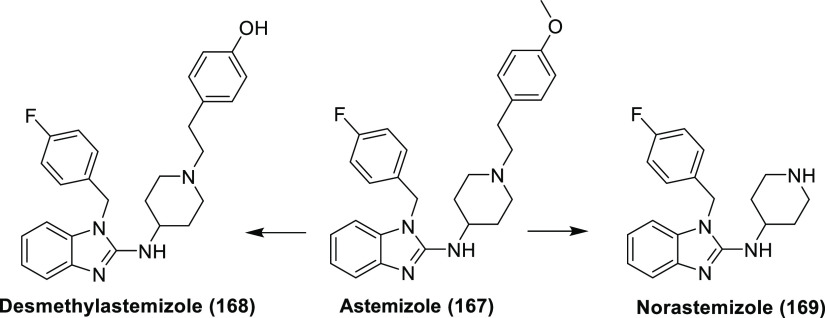
Astemizole and Its Metabolites

The blocking of potassium channels could be due to the accumulation of the drug given its long duration of action as well as its active metabolite desmethylastemizole **168** (Scheme 64) which has a half-life of about 12 days. Both astemizole and desmethylastemizole could block the cardiac potassium channels, causing impaired repolarization of the heart and, therefore, ventricular arrhythmias. On the other hand, norastemizole **169** (Scheme 64), another active metabolite of astemizole, has a shorter half-life and does not seem to be involved in this blockade.

##### 6.1.1.6 Flunitrazepam

**Scheme 65:**
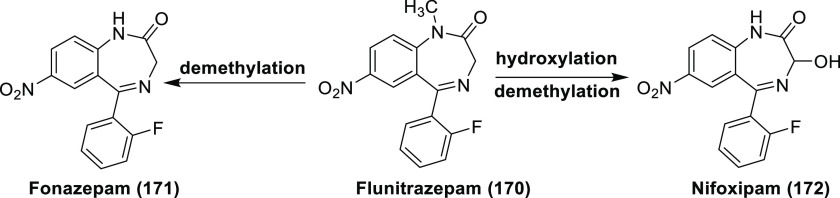
Flunitrazepam and Its *in Vivo* Metabolites

Flunitrazepam **170** (Scheme 65) is a benzodiazepine central nervous depressant, a class of drugs used as sedative–hypnotics compounds to treat anxiety, insomnia, and sleep disorders. It has a nitro group and a fluorine atom in the molecule, both of which increase the hypnotic effects of benzodiazepines. The sedative, antianxiety, and muscle-relaxing properties of flunitrazepam are similar to other benzodiazepine derivatives, but its sedative and sleep-inducing properties are more pronounced and longer lasting.

Flunitrazepam was discovered and patented by the Swiss pharmaceutical company Hoffman-La Roche in 1962. It was marketed in 1974 with the brand name *Rohypnol* for the treatment of severe insomnia and was used as preanesthetic drug.^302^ Its metabolism involves hydroxylation and demethylation processes, leading to the formation of two active metabolites, fonazepam **171** (desmethylflunitrazepam) (Scheme 65) and nifoxipam **172** (3-hydroxy-desmethylflunitrazepam) (Scheme 65).^303^

The most frequently reported side effects associated with this drug include physical and psychological dependence, reduced sleep quality resulting in somnolence, respiratory depression, and loss of motor control or decreased reaction time, all adverse effects related with benzodiazepine derivatives in general. Cases of intoxication with flunitrazepam were not initially reported, possibly due to its difficult detection in serum. Severe cases of toxicity began to appear in combination with alcohol abuse, leading to flunitrazepam’s classification as a dangerous drug. Because of its potent sedative hypnotic activity, this drug was also used in rape cases. Consequently, the drug was withdrawn from the market.^304^

##### 6.1.1.7 Mibefradil

**Scheme 66:**
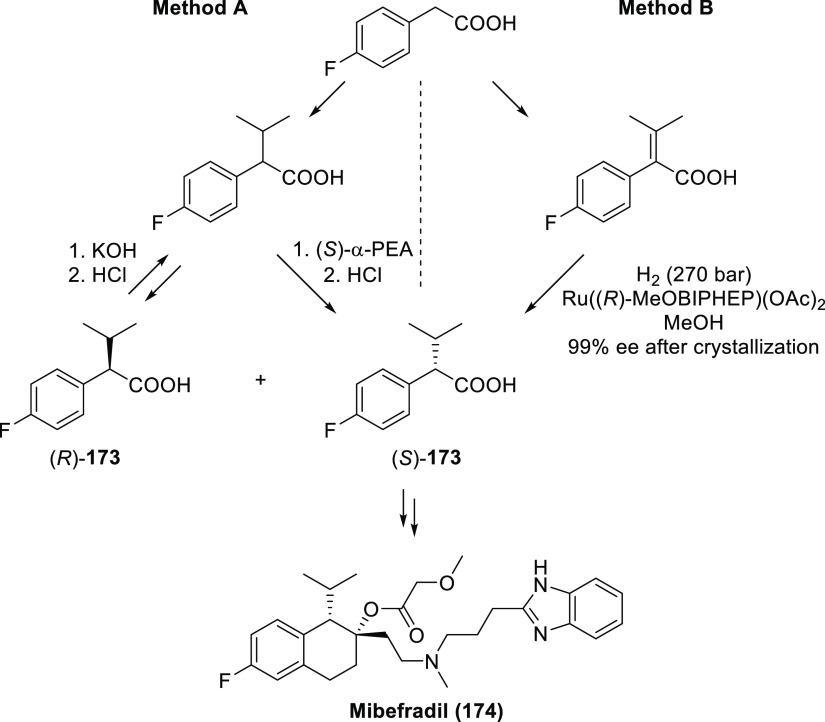
Synthesis of Mibefradil

Mibefradil **174** (Ro 40-5967) (Scheme 66) was marketed under the trade name *Posicor* and is a tetralol derivative marketed by Hoffmann-La Roche in 1997 used for the treatment of hypertension and chronic angina pectoris. This drug is a calcium channel blocker, relaxing blood vessels and increasing the supply of blood and oxygen to the heart. Mibefradil blocks both L-type and T-type calcium channels but has a higher affinity for the latter.^305^

Its benzimidazoyl-substituted tetraline structure contains two chiral centers and was used as a single enantiomer. Two possible synthetic routes were reported by Schmid and co-workers in 1997: the first employing a base-catalyzed resolution of (*R*)-**173** into the desired (*S*)-**173** enantiomer (Scheme 66, Method A) and the second through asymmetric hydrogenation (Scheme 66, Method B). The latter resulted in higher total yields.^306^

Mibefradil was withdrawn from the market by Roche in 1998 due to continued reports of sometimes deadly adverse effects from multiple interactions with other drugs, such as common antibiotics, antihistamines, and anticancer drugs. Mibefradil inhibits CYP 3A4 and CYP 2D6, liver enzymes involved in normal drug metabolism. Due to this, the metabolism of other drugs was blocked, resulting in the plasma concentrations of coadministered drugs increasing to dangerous levels.^307^ However, mibefradil has been investigated for its anticancer potential, minimizing unwanted drug–drug interactions by short-term dose exposure. This research could show the importance of T-type calcium channel blockers in cancer treatment.^308,309^

##### 6.1.1.8 Sertindole

**Scheme 67:**
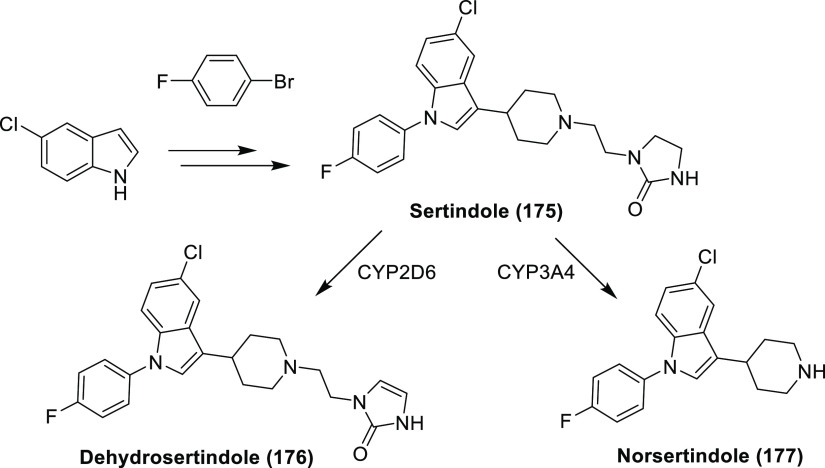
Synthesis and Metabolites of Sertindole

The atypical second-generation antipsychotic agent sertindole **175** (Scheme 67) was discovered and patented by the Danish pharmaceutical company H. Lundbeck A/S in collaboration with Abbot Laboratories for the treatment of schizophrenia. This drug has an affinity for dopamine D2 and serotonin 5-HT2A and 5-HT2C receptors, lending itself to the treatment of anxiety, hypertension, and cognitive disorders. In contrast to other antipsychotics, this drug is not associated with sedative effects.^310^

The synthesis of this phenylindole derivative has been recently reported by Kumar and co-workers with the aim of identifying the impurities formed during its preparation.^311^ This synthesis implies the previous copper-catalyzed N-arylation of 5-chloroindole with 4-fluorobromobenzene (Scheme 67).

Sertindole is metabolized by CYP2D6 to dehydrosertindole **176** and by CYP3A into norsertindole **177**, *both with* insignificant pharmacological activity.

This drug was authorized and introduced into the market in 1996 but was voluntarily withdrawn in 1998 after numerous cardiac adverse effects were reported.^312^ Various studies revealed that increased drug concentration could lead to increased risk of QTc prolongation.^313,314^ However, after a reevaluation of its risks and benefits, sertindole was reintroduced into the European market in 2005, and the drug is currently available in several countries.^315^

#### 6.1.2 Aromatic Trifluoromethyl-Substituted Compounds

Although the trifluoromethyl group (CF_3_) is a component of several commonly used drugs, studies into the metabolism of trifluoromethyl-substituted aromatic compounds are far less common. The trifluoromethyl group, like fluorine itself, is a strong electron-withdrawing group in aromatic systems, in contrast to a methyl group. Although the CF_3_ group itself is not known to suffer metabolic degradation, metabolic attack can occur at *ortho* and *para* positions in aromatic substituted systems by cytochrome P450 enzymes.^172^

In order to better define the safety and efficacy of drugs, it is important to understand how metabolites act in the body since they can present very different activity from that of the parent compound.

A collection of aromatic trifluoromethyl-substituted compounds that have been withdrawn from the market due to the formation of metabolic species with undesirable side effects are reviewed in this section.

##### 6.1.2.1 Fenfluramine/Fen-phen/Dexfenfluramine

**Figure 6:**
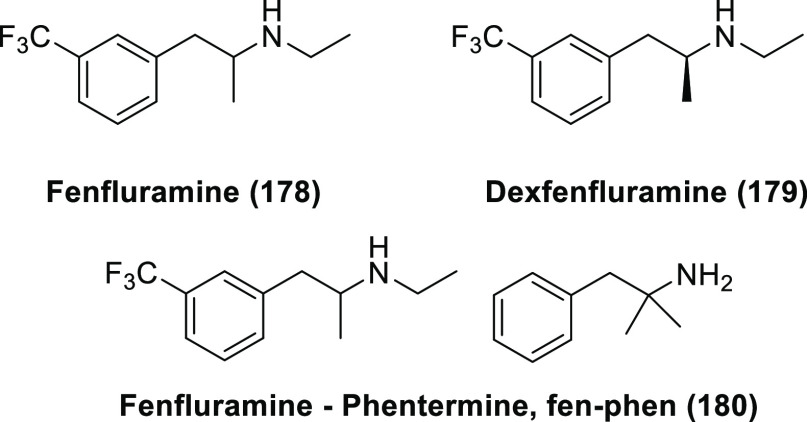
Fenfluramine and derivatives.

Racemic fenfluramine **178** (trade name *Pondimin*) (Figure 6) was approved for sale in 1973 and acts as an appetite suppressant for the short-term treatment of obesity marketed by American Home Products. The eutomer dexfenfluramine **179** (*Redux*) (Figure 6) was later developed as a single enantiomer drug by Wyeth–Ayerst Laboratories and approved for sale in 1996.

Fenfluramine was available alone and in combination with phentermine (*Ionamin*), another appetite suppressant that has been on the market since 1959. The combination was called fen-phen (**180**, Figure 6) and had a better business outcome. Although the activity of both drugs is similar, these compounds were combined for the equilibrium between their adverse effects, drowsiness and sedation in the case of fenfluramine and irritability or insomnia in the case of phertermine.^316^

**Scheme 68:**
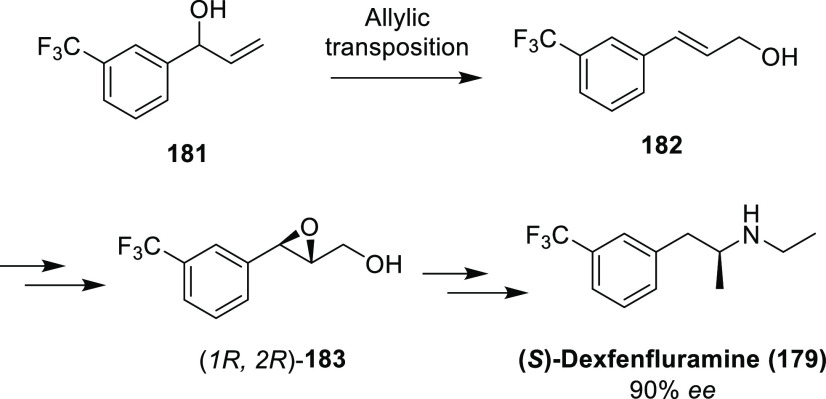
Synthesis of (*S*)-Dexfenfluramine

Duhamel and co-workers developed a synthetic route toward the active enantiomer (*S*)-dexfenfluramine with good enantiomeric excess, using the Sharpless method for asymmetric epoxidation of primary allylic alcohol **182** (Scheme 68).^317^

**Scheme 69:**
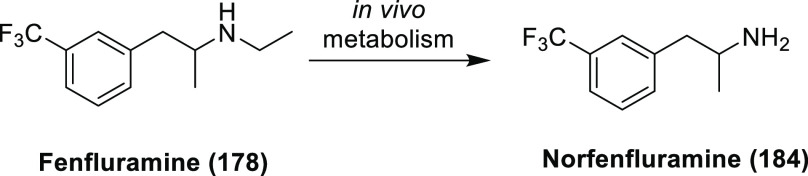
Fenfluramine Metabolite

Fenfluramine acts as a serotonin-releasing agent and selectively inhibits its reuptake. An increase in serotonin levels results in a feeling of fullness and, consequently, loss of appetite. However, the use of these appetite suppressants was associated with cases of primary pulmonary arterial hypertension (PAH) and changes at the heart valves.^318^ Through receptorome screening, Roth and Setola showed the role of the metabolite formation in the undesirable side effects observed of these drugs.^316^ The activation of mitogenic 5-HT_2B_ receptors by norfenfluramine **184** (Scheme 69), a fenfluramine metabolite, revealed the possible molecular mechanism responsible for the adverse cardiopulmonary effects of fenfluramine.

Due to the high incidence of cardiac valve problems reported after fen-phen combination treatment, as well as after fenfluramine or dexfenfluramine alone, these drugs were removed from the market in 1997.

##### 6.1.2.2 Benfluorex

**Scheme 70:**
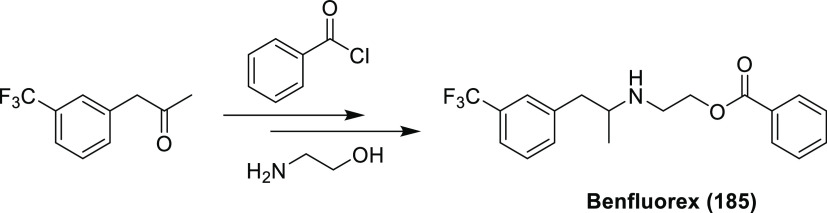
Synthesis of Benfluorex^320^

Benfluorex **185** (Scheme 70), a structural analogue of fenfluramine, is an anorectic and hypolipidemic agent marketed by the French pharmaceutical company Servier Laboratories in 1976 under the trade name *Mediator*.^319^

The beneficial effects of benfluorex in the treatment of obesity disorders were masked by the adverse effects observed. Like with fenfluramine, an increased risk of valvular heart disease (VHD) and pulmonary artery hypertension was the reason behind the benfluorex withdrawal in 2009. It was assumed that benfluorex induced VHD through formation of norfenfluramine, a common metabolite with fenfluramine with high affinity to 5-HT_2B_ receptors.^321^

##### 6.1.2.3 Tolrestat

**Scheme 71:**
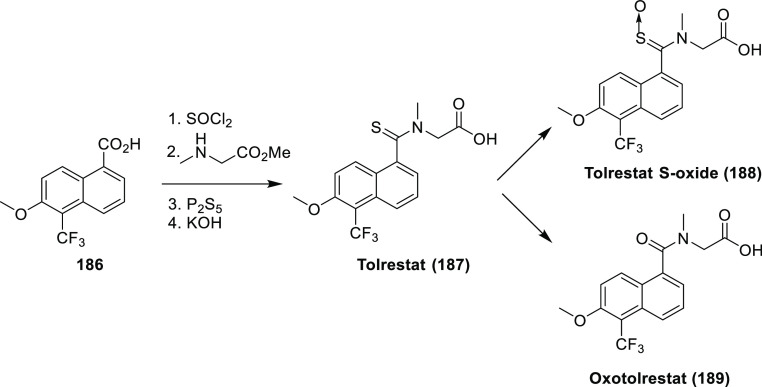
Synthesis and Metabolism of Tolrestat

The aldose reductase inhibitor tolrestat **187** (Scheme 71) was developed for the control of certain diabetic complications, including diabetic neuropathy, retinopathy, and nephropathy.^322^ As a group of metabolic disorders related with lack of insulin, diabetes can lead to chronic hyperglycemia. Several studies have linked this glucose-metabolism disorder with the enzyme aldose reductase, which is involved in the reduction of a variety of aldehydes as well as plays an important role in inflammatory disorders. In this sense, the development of effective aldose reductase inhibitors represents one of the main goals to combat this disease.^323^

Tolrestat was approved for sale in several countries under the trade name *Alredase*. Its naphthalene nucleus and thioamide moiety are the key for inhibition of aldose reductase.^324^ This structure was synthesized using carboxylic acid intermediate **186** by treatment of its acid chloride and methyl sarcosinate (Scheme 71).^325^

To identify the possible metabolites of tolresat, several studies were carried out in various species, including humans. In one report, metabolites **188**, corresponding to thioamide oxidation, and **189**, corresponding to O-demethylation, were both identified in rat urine and bile.^326^

In 1996, the manufacturer Wyeth withdrew tolrestat from the market worldwide due to reported cases of hepatic necrosis associated with its use.^327^

### 6.2 Aliphatic Substitution

In contrast to the previously discussed aromatic compounds, which are generally more resistant to metabolism, the release of fluoride from aliphatic compounds is readily achieved by hydroxylation in the α position relative to the carbon–fluorine bond and subsequent elimination of hydrofluoric acid, leading to the formation of a ketone or an acyl halide.^172^

#### 6.2.1 Odanacatib

**Scheme 72:**
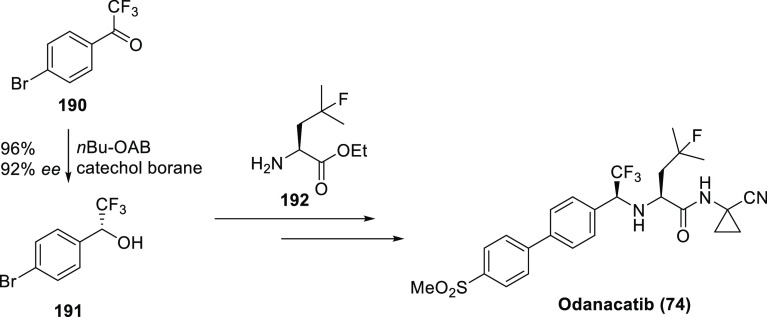
Synthesis of Odanacatib

Odanacatib **74** (MK-0822) (Scheme 72) is a selective inhibitor of Cathepsin K activity, developed by Merck for the treatment of osteoporosis and bone metastasis.^328^ Osteoporosis is characterized by abundant bone loss, causing skeletal fragility, along with an increased risk of fracture. Osteoclasts, a type of bone cell that breaks down bone tissue, are directly involved in the disturbance of bone remodeling when bone resorption exceeds bone formation. These cells express various enzymes for the degradation of the bone matrix, including Cathepsin K, a lysosomal cysteine protease with collagenase properties. Odanacatib was shown to be a potent inhibitor of this enzyme, reducing bone resorption and preserving bone formation.^329^

O’Shea and co-workers reported a practical and enantioselective synthesis of odanacatib on a multikilogram scale in 2009.^330^ The nucleophilic displacement of the appropriately activated chiral α-trifluoromethylbenzyl alcohol **191**, which had been previously obtained via oxazaborolidine-catalyzed enantioselective reduction (OAB) of ketone **190**, with α-amino ester **192**, provided odanacatib **74**. This chromatography-free synthesis was developed in six steps with a 61% global yield (Scheme 72).

Although the long-term clinical utility of this drug to reduce the risk of osteoporotic fractures was fully determined in Phase III clinical trials, in 2016 the company decided to discontinue its development after reports of major cardiovascular events and a higher risk of strokes.

#### 6.2.2 Begacestat

**Scheme 73:**
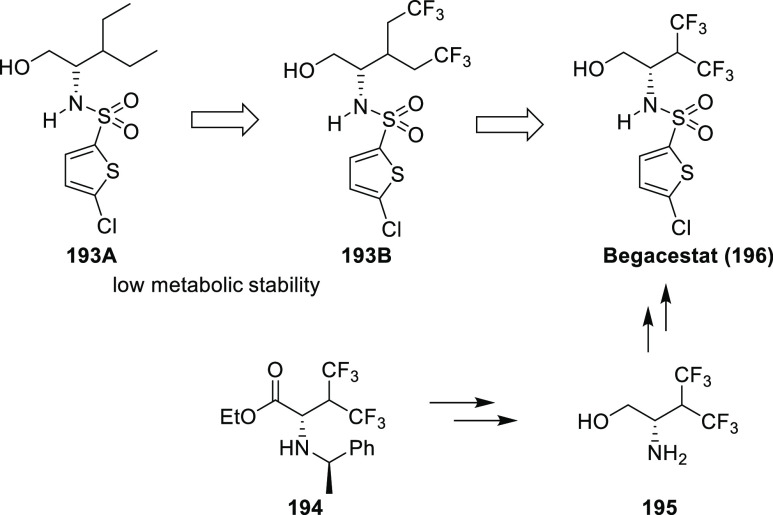
γ-Secretase Inhibitors and Synthesis of Begacestat

Begacestat **196** (Scheme 73), developed by Wyeth (now Pfizer) with code name GSI-953, is a selective thiophene sulfonamide derivative used as a γ-secretase inhibitor (GSI) in the treatment of Alzheimer’s disease.

Alzheimer’s disease is a progressive neurodegenerative disorder characterized by amyloid β-peptide (Aβ) accumulation, which is produced by the transmembrane protease γ-secretase.^331−333^ Thus, the inhibition of γ-secretase activity is the principal function of GSI-953. Begacestat was passed to phase I clinical trials in 2007; however, it was discontinued in 2010 for unknown reasons.

In 2008, Mayer and co-workers reported the synthesis of begacestat as a fluorinated derivative of previous γ-secretase inhibitors developed by the same authors (Scheme 73, **193A** and **193B**). After several analyses of efficacy and metabolic stability, the authors found that the introduction of trifluoromethyl groups avoided metabolism at certain oxidation sites in the side chain, granting better metabolic stability. For its preparation, they started from the chiral α-amino ester **194**. Reduction and treatment with H_2_ using a palladium catalyst provided the amino alcohol **195**, a key intermediate in the synthesis of begacestat **196** (Scheme 73).^334^

## 7 Fluoride Toxicity

### 7.1 Digestion and Absorption, Distribution, and Elimination of Fluoride

**Figure 7:**
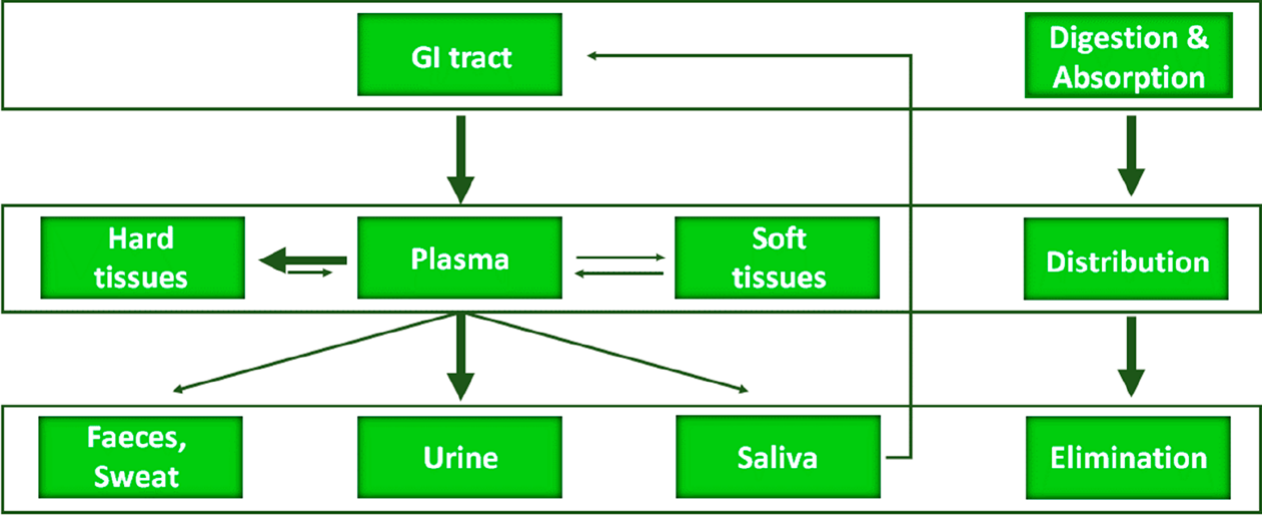
General features of fluoride digestion and absorption, distribution, and elimination.

The general features of fluoride digestion and absorption, distribution, and elimination are schematically presented in Figure 7.

#### 7.1.1 Fluoride Absorption

Under normal conditions most of the fluorine enters the body by ingestion. In stomach lumen, fluoride formed during digestion is largely converted to weak acid hydrogen fluoride (HF) with a p*K*_a_ of 3.19.^92^ The higher acidity of the stomach speeds up the process of absorption by passive diffusion.^335^ The coefficient of permeability of lipid bilayer membranes to HF is 1 million times higher than that of F^–^.^336^ About 20–25% of fluoride is absorbed from the stomach in a pH-dependent process, and about 75–80% of the remaining fluoride is absorbed from the small intestine in a pH-independent process.^337−339^ Ingestion of well-soluble compounds like NaF results in faster absorption, whereas less soluble fluoride compounds like CaF_2_ slow absorption.^340^

The bioavailability of fluoride from different foods in adults varies between 2 and 79% in dependence on the factors such as amount of ingested food, the presence and solubility of minerals in ingested food, emptying the stomach, the presence of bile salts, and concentrations of pepsin.^121^

#### 7.1.2 Fluoride in Plasma

The half time for fluoride absorption is approximately 30 min, and peak plasma concentration usually occurs within 30–60 min.^341−346^ Baseline plasma fluoride levels are generally reached within 3–11 h after ingestion, depending on the amount ingested.^347^ Fluoride in plasma is available as ionic F^–^ and nonionic (organic or bound fluoride).^348^ Ionic F^–^ is significant in dentistry, medicine, and public health.^349^ Plasma fluoride concentration is not homeostatically regulated, which means it increases or decreases depending on the amount of fluoride ingestion, deposition or removal from the soft and hard tissues, and excretion.^350^ The fluoride balance can be negative if chronic intake is reduced sufficiently to allow plasma concentration to fall, which promotes demobilization of F^–^ from calcified tissues.^144^

#### 7.1.3 Distribution and Elimination

After the absorption, fluoride is rapidly distributed in plasma and then to all tissues and organs. About 50% of the daily intake of fluoride is, within 24 h, deposited mainly in calcified tissues—such as bones and teeth, as well as calcium-containing glands such as the pineal gland. These tissues contain approximately 99% of the body’s fluoride, and the remainder is distributed between the blood and soft tissues, where rapidly a steady-state distribution between extracellular and intracellular fluids is established.^350,351^ The remaining 50% is predominantly excreted via the kidneys and is influenced by a number of factors, including glomerular filtration rate, urinary flow, and urinary pH.^347,351^ This 50:50 distribution is strongly shifted to greater retention in the very early and probably toward greater excretion in the later years of life.^352−354^ Young children can retain up to 80% of fluoride due to increased uptake by the developing skeleton and teeth.^355,356^ To a lesser extent, fluoride is also excreted in the faeces, sweat, and saliva.^357^

### 7.2 Mechanisms of Fluoride Cytotoxicity

The effects of fluoride on cellular metabolism and physiology vary according to the cell type, concentration, and duration of exposure.^358^ The main toxic effects of fluoride in cells can be ascribed to inhibition and sometimes stimulation of a variety of enzymes by a mechanism dependent on the type of enzyme affected.^358−360^

Fluoride effects on cellular processes include the influence on gene expression, cell cycle, proliferation and migration, respiration, metabolism, ion transport, secretion, endocytosis, and oxidative stress, ultimately leading to necrosis or apoptosis.^358,359,361−365^ Necrosis has been observed as a primary mechanism of cell death after a short exposure (≈1 h) to fluoride at relatively high concentrations (≈100 mmol).^366^ At lower concentrations (a few mmol or even less) fluoride triggers apoptotic cell death from different tissues and organs by the activation of caspases in both intrinsic (mitochondrial) and extrinsic (death receptor) pathways, which converge on caspase-3 activation.^358,361,364,365,367−369^

**Figure 8:**
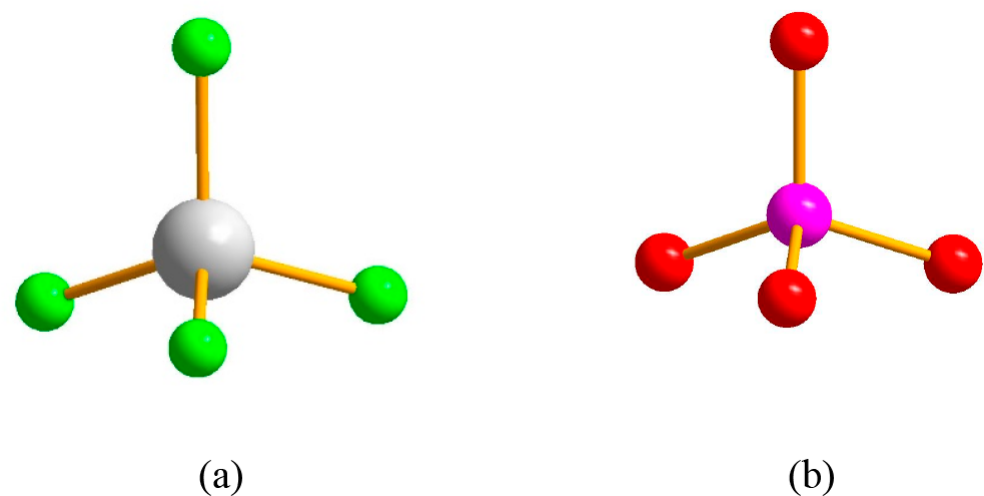
Schematic drawing of (a) AlF_4_^–^ and (b) PO_4_^3–^ ions.

Fluoride interferes with enzymes as the F^–^ ion or as aluminum fluoride (AlF_*x*_^3–x^, *x* = 1–6, abbreviated as AlF_*x*_) complexes, which are able to activate G-protein-coupled receptors (GPCRs) at several times lower concentrations than either Al^3+^ or F^–^ acting alone.^29^ The average stoichiometry of the complexes formed between fluoride in the presence of even trace amounts of aluminum is mainly dependent on the excess of fluoride and the pH.^370^ The AlF_*x*_’s activate a G-protein-signaling pathway by binding to the active site of guanosine diphosphate (GDP) as a tetracoordinate AlF_4_^–^^371,372^ or AlF_3_(OH)^−^^373,374^ ion. The spatial and structural similarities of AlF_4_^–^ and PO_4_^3–^ ions are schematically presented in Figure 8.

Initially, it was assumed that the AlF_4_^–^ occupies the γ-phosphate binding site on the protein and together with bound GDP makes the G protein act as if it has bound guanosine triphosphate (GTP).^371^ Later, the bound AlF_4_^–^ was concluded to mimic the PO_4_^3–^ in its pentavalent transition state during hydrolysis.^375^ In addition, beryllium forms soluble ionic complexes (BeF*_*x*_^x^*^–2^, *x* = 1–4) which were found to be as effective a cofactor as aluminum at μmol concentrations and in the presence of mmol fluoride concentrations.^376,377^ Effects of AlF_*x*_ on cell-signaling pathways can result in changes in gene expression, cytoskeletal reorganization, intracellular vesicle trafficking, and nucleocytoplasmic transport.^378^

The actual concentrations of fluoride in organisms may be lower (a few mmol) than those used in laboratory experiments. However, the fluoride dose–response relationship is not always monotonic. In certain studies, a paradoxical effect of fluoride with inhibitory or stimulatory impact being greater at a lower level of intake than at a higher level (hormesis effect) was observed.^358,379,380^ Therefore, stating a “safe” dose of fluoride is illusive—environmental exposure, dietary patterns, certain medical conditions, and genetic background all play a major role in influencing the risk to fluoride toxicity.^381−383^

### 7.3 Toxicity in Relation to the Exposure to Fluoride

The margin between the beneficial and deleterious effects of fluoride appears to be narrow. Therefore, the toxic effects of fluoride on human health should be evaluated considering factors such as (1) fluoridation of public water supplies at safe levels; (2) proven toxic effects in cells; (3) possible occurrence of adverse effects at the intakes lower than the AI; (4) awareness that adverse effects are often not recognized and are ascribed to other causes; (5) conflicting results of the studies; (6) cautious interpretation of the studies’ results; and (7) awareness that correlation is not necessarily a causation. Acute toxicity involves harmful effects in an organism through a single or short-term exposure to a toxic substance. On the other hand, chronic toxicity is a result of continuous or repeated exposure of an organism to a toxic substance.

#### 7.3.1 Acute Toxicity

The toxicity of fluoride depends on the solubility of the compound ingested—the more soluble salts of inorganic fluorides, such as sodium or potassium fluoride, are more toxic than weakly soluble or insoluble.^340^ Acute high oral exposure to fluoride may lead to (with increased seriousness of observed symptoms) nausea, vomiting, abdominal pain, diarrhea, drowsiness, headaches, polyuria and polydipsia, coma, convulsions, cardiac arrest, muscle paralysis, carpopedal spasm, spasm of the extremities, and death.^384−386^

The probable toxic dose (PTD) for children, defined as the dose of ingested fluoride that should trigger immediate therapeutic intervention and hospitalization, because of the likelihood of serious toxic consequences is set at 5.0 mg kg BW^–1^.^386^ Lower intakes than that should however not be regarded as eventually harmless. The most frequently cited estimate for a reasonable certainly lethal dose (CLD) of sodium fluoride is set between 32 and 64 mg kg BW^–1^ of fluoride, which corresponds to 5–10 g of sodium fluoride for a 70 kg person.^386,387^

#### 7.3.2 Chronic Toxicity

**Figure 9:**
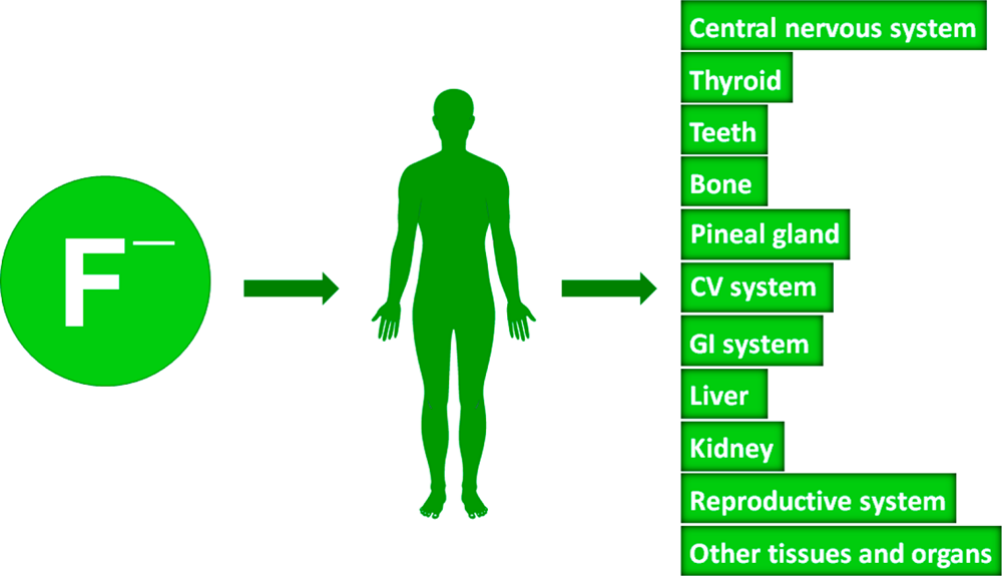
Fluoride effects different tissues and organs of a human body.

Excessive intake of fluoride during a prolonged period of time can result in (1) development of dental fluorosis in children; (2) skeletal fluorosis in both children and adults; and (3) damage to virtually all nonskeletal tissues. Environmental exposure, dietary patterns, certain medical conditions, and genetic background could play a major role in influencing the risk to fluoride toxicity.^381−383^ Symptoms of chronic fluoride intoxication include: (1) irritable bowel syndrome; (2) polyuria and polydipsia; (3) extreme fatigue/exhaustion/loss of muscle power; (4) insomnia; (5) low hemoglobin; (6) depression; (7) high cholesterol and high blood pressure; (8) joint pain; (9) frequent breaking of bones; (10) disabled children with bowleg, knock-knee, short stature, and mental retardation; and (11) pregnant women with anemia, low birth weight babies, preterm deliveries, intrauterine death, neonatal death, and infant mortality.^388,389^ Some of the fluoride effects on different tissues and organs of a human body are shown in Figure 9.

##### 7.3.2.1 Dental Fluorosis

Enamel fluorosis and primary dentin fluorosis are caused by excessive intake of fluoride during critical periods of amelogenesis of both primary and secondary teeth. Altered protein/mineral interactions are in part responsible for retention of amelogenins and the resultant hypomineralization that occurs in fluorosed enamel.^390,391^ Other indirect effects of fluoride on cells at different stages of formation are likely to be expected. Fluorosed enamel has a higher protein content than nonfluorosed enamel, resulting in increased porosity.^392−394^

There is a clear linear relationship between fluoride dose and enamel fluorosis regardless of whether fluoride is ingested from drinking water or diet, supplements, or other sources.^395,396^ This suggests that no threshold value exists below which the effect of fluoride on dental enamel will not be manifested.^136^ A threshold of 0.03 mg day^–1^ kg BW^–1^ has been suggested for the appearance of dental fluorosis.^136,396^

The “gold standard” for quantifying dental fluorosis is Dean’s index^397^ suggested in its final form in 1942, though other indexes including the widely used Thylstrup and Fejerskov Fluorosis Index,^398^ which has an expanded range for the more severe forms of dental fluorosis, were also developed. According to Dean’s index, dental fluorosis is classified on a scale from 0 to 4 into six classes from normal or unaffected enamel to very mild fluorosis with “small, opaque, paper white areas scattered irregularly over the tooth but not involving as much as approximately 25% of the tooth surface” to severe fluorosis, when “all enamel surfaces are affected and hypoplasia is so marked that the general form of the tooth may be affected”.

Dental fluorosis is due to fluoride prevention benefits often presented merely as a cosmetic problem and not as an adverse effect.^396^ Its prevalence is in increase.^399−401^ Endemic fluorosis is present in many countries, in particular in Africa and Asia due to contaminated drinking water, pollution from coal burning, and brick-tea drinking.^357,365,402^

##### 7.3.2.2 Skeletal Fluorosis

Skeletal fluorosis is a result of high chronic intake of fluoride, which accumulates progressively in the bone. Excessive fluoride intake disrupts a dynamic balance of bone turnover and metabolism by influencing different signaling pathways at a cellular level.^365,403^

Early symptoms of skeletal fluorosis include stiffness and pain in the joints. Crippling skeletal fluorosis is associated with osteosclerosis, calcification of tendons and ligaments, bone deformities, and neurological defects associated with compression of the spinal cord.^351^

Skeletal fluorosis with adverse changes in bone structure may be observed when drinking water contains 3–6 mg L^–1^ of fluoride.^114,404^ Crippling skeletal fluorosis develops at water fluoride concentrations higher than 10 mg L^–1^.^27,404^ Despite relatively shorter exposure, skeletal fluorosis can develop also in children living in fluoride endemic areas.^158,405^

##### 7.3.2.3 Gastrointestinal System

Symptoms associated with mild fluoride toxicity are nonspecific and may be attributed to colic or gastroenteritis.^406^ The primary gastrointestinal (GI) effects associated with chronic or acute exposure to fluoride include nausea, pain, and vomiting.^407^ GI symptoms are commonly observed in humans suffering from skeletal fluorosis or osteoporosis patients treated with fluoride.^27,408^ The irritation of gastric mucosa is due to HF formed under acidic conditions of the stomach lumen. The effects on the stomach resemble those of acetylsalicylic acid (p*K*_a_ 3.6), which causes structural and functional alterations of the gastric mucosa.^409−411^

The concentration of fluoride in drinking water of 4 mg L^–1^ was suggested as the concentration at which approximately 1% of the population might experience GI symptoms; as the fluoride concentration in drinking water increases, the percentage of the population with GI symptoms also increases.^407^

To our knowledge, no human studies have systematically studied the GI effects of low fluoride exposure.

##### 7.3.2.4 Kidney and Liver

Kidneys followed by the liver accumulate higher concentrations of fluoride than other organs and tissues.^347,351^ This suggests that the renal system and liver might be at a higher risk of fluoride toxicity than most soft tissues.^347,407^ The position statement regarding fluoridation developed in Australia says that “There is no evidence that consumption of optimally fluoridated drinking water increases the risk of developing chronic kidney disease (CKD), although only limited studies addressing this issue are available.”^412^ The statement was accepted with criticism that the “absence of evidence is not evidence of absence”.^413^ Of special concern should be patients with impaired renal failure, who may develop skeletal fluorosis even at 1 mg L^–1^ of F^–^ in drinking water.^414,415^ Recently, a direct correlation between CKD and the consumption of excess amounts of fluoride showing immediate adverse effects on the tubular area of the kidneys in animals and humans was suggested.^416,417^ In children, renal fluoride clearance rates are lower than in adults.^352^ Consumption of drinking water with fluoride concentration above 2.0 mg L^–1^ in children was suggested to cause damage to the kidney, which increases with the F^–^ concentration.^418^ The prevalence of urolithiasis in fluoride endemic areas with water containing between 3.5 and 4.9 mg L^–1^ of F^–^ was reported to be 4.6-fold higher than in the nonendemic area with water F^–^ below 0.5 mg L^–1^, probably also because of malnutrition.^419^ Recently, geogenic fluoride has been hypothezed as one of the causative factors in developing chronic kidney disease of multifactorial origin.^420^

Fluoride has been shown to induce morphological changes and liver dysfunction.^365^ The mechanism for understanding the dose and time dependency of the effects of fluoride in the liver has been proposed.^421^

Adverse renal and hepatic effects of fluoride were found in rats at early stages of fluoride intoxication, where alterations in liver enzyme activities were first observed and then followed by renal damage.^422^ In children, the dose–effect relationship between the concentration of fluoride in drinking water (*C*_F_^–^ > 2 mg L^–1^) and damage to liver and kidney functions was observed.^423^ The results of a recent study conducted in the US suggested that in adolescents exposure to drinking water generally containing less than 1 mg L^–1^ of F^–^ may contribute to complex changes in kidney and liver-related parameters.^424^

The liver is the primary organ for oxidative detoxification including the detoxification of organofluorides.^407^ Nephrotoxicity due to fluoride was reported in patients who received methoxyflurane anesthesia for surgery.^425^ The kidney and liver function of children and adults should be considered in relation to the recommended fluoride concentration of drinking water.

##### 7.3.2.5 Cardiovascular System

Calcification in the aorta of rabbits has been observed after 17–24 months of daily administration of 10 mg of NaF kg BW^–1^.^426^ The calcification of medial elastic fibers eventually contributes to increased arterial stiffness, resulting in cardiovascular diseases.^427^ Excessive exposure to fluoride could eventually increase plasma Endothelin-1 levels,^428^ which is suggested to play a crucial role in several cardiovascular diseases, like congestive heart failure (CHF), hypertension (HTN), atherosclerosis (AS), and renal disease.^429,430^ Already, early fluoride exposure may favor the atherosclerotic process, which could contribute to the development of cardiovascular diseases.^431^

Dental fluorosis in children living in the fluoride endemic area with water F^–^ concentration above 1.2 mg L^–1^ was suggested as a risk factor for some cardiovascular system dysfunctions such as arrhythmias or syncope.^432^ The severity of skeletal fluorosis in the fluoride endemic area with water F^–^ concentration between 4.1 and 8.6 mg L^–1^ has been reported to be related to the severity of abnormal cardiac function.^433^ In contrast, no effect on the electrocardiogram was reported in a study of patients with dental fluorosis living in a fluoride endemic area consuming water with fluoride concentration above 1.2 mg L^–1^.^434^ Aortic elasticity and left ventricular diastolic and global functions were impaired in patients with dental fluorosis living in the fluoride endemic region with natural water fluoride concentrations above 1.2 mg L^–1^.^435,436^ Fluoride exposure from drinking water with fluoride concentrations between less than 1.2 to more than 3.0 mg L^–1^ in adults living in fluoride endemic areas was related to the prevalence of carotid artery atherosclerosis.^437^ An increase of hypertension prevalence with the increase of fluoride levels in drinking water from low to endemic concentrations was observed.^438,439^

In nonendemic regions, naturally present fluoride in water was suggested to have a slight protective effect with respect to coronary heart disease.^440^ Natural contents of fluoride in water between less than 0.3 mg L^–1^ to 1.5 mg L^–1^ and above appear not to be associated with myocardial infarction.^441^

Association of fluoride with toxic or beneficial effects on the cardiovascular system needs more experimental and epidemiological studies, including adequate control of confounding cofactors.^442^

##### 7.3.2.6 Thyroid

Iodine is involved in the synthesis of the thyroid hormones, thyroxine (T4) and triiodothyronine (T3), which is controlled by thyroid-stimulating hormone (TSH).^443^ The thyroid hormones have multiple effects on metabolism and are associated with different systems and organs, e.g. cardiovascular, central nervous and digestion system, bone growth, and breathing. The parathyroid hormone has an important role in bone remodeling.^444^

In the 1950s, fluoride was used to treat hyperthyroidism with a daily dose between 2 and 5 mg of fluoride, which is within the range of estimated daily intake of fluoride in adults (see section 4.4.2).^357,445^ Much of the recent research has been devoted to the impact of fluoride on the thyroid.^446^

Based on the examination of the impact of fluoride on thyroid function, it was concluded that fluoride is an endocrine disruptor with the potential to disrupt the function of tissues that require iodine.^407,447^ The impact of iodine deficiency can be exacerbated by fluoride, which is of special concern in children.^448^ Daily exposures to fluoride between 0.05 and 0.13 mg kg BW^–1^ have been associated with adverse thyroid effects among iodine-sufficient people and between 0.01 and 0.03 mg kg BW^–1^ among iodine-deficient people.^407^

In England, the general practitioners that practice in areas with water fluoride concentration of 1 mg L^–1^ were nearly twice as likely to report high hypothyroidism prevalence in comparison to the nonfluoridated area.^449^ Impacts of fluoride on TSH and T3 hormones were suggested even at low water F^–^ concentrations below 0.5 mg L^–1^.^450^ In Canada, at the population level, fluoride exposure was reported not to be associated with impaired thyroid functioning in a time and place where multiple sources of fluoride exposure, including community water fluoridation, exist.^451^ Also in Canada, adults who had moderate-to-severe iodine deficiencies and higher levels of urinary fluoride were shown to be at an increased risk for underactive thyroid gland activity.^452^

Secretion of the parathyroid hormone (PTH) has been suggested to be directly influenced by fluoride.^453,454^ Higher PTH levels were observed in patients with endemic fluorosis than in controls.^455^

Further studies are needed to elucidate the mechanisms of fluoride impact on thyroid functions and the possible outcomes among different populations and settings.

##### 7.3.2.7 Central Nervous System (CNS)

Fluoride was identified as an industrial chemical known to cause developmental neurotoxicity in humans.^456^ A justified critique of the paper was that the authors should have made clear that listing fluoride as a neurotoxin does not apply to fluoridation at the recommended levels of 0.7–1.2 mg L^–1^.^457^ As a reply to this critique, the authors commented that “*the fact that a trace element has beneficial effects at low doses in specific tissues does not negate the possibility that neurotoxicity might also be occurring, especially at increased levels of exposure*”.^458^

According to the literature, fluoride is capable of crossing the placenta^459,460^ and brain barrier,^461^ and the toxicity of even low concentrations of F^–^ is enhanced in the presence of Al^3+^.^462^ Systematic reviews and meta-analysis of the studies published between 1989 and 2015 support the possibility of adverse effects of exposure to high levels of fluoride on children’s neurodevelopment.^463−465^ Research on a possible relationship between fluoride exposure and neurotoxic effects in children continues to receive attention. A lot of data come also from Mexico^466,467^ and Canada.^468−471^ Higher prenatal fluoride exposure (via fluoridated salt containing 250 mg kg^–1^ of F^–^ and naturally present fluoride in water with concentrations between 0.15 and 1.38 mg L^–1^ of F^–^), as measured by maternal urinary fluoride (MUF) during pregnancy, was associated with lower scores on tests of cognitive function in the offspring at the age of 4 years and between 6 and 12 years^466^ and more behavioral symptoms of inattention but not hyperactivity or impulse control between 3 and 12 years of age.^467^ Maternal exposure to fluoride through fluoridated water (*C*_F_^–^ = 0.59 mg L^–1^), as measured by MUF during pregnancy, was associated with lower IQ scores (assessed at the age 3–4 years) in offspring in comparison to a control group living in nonfluoridated areas (*C*_F_^–^ = 0.13 mg L^–1^).^468^ An exposure to fluoride in water (*C*_F_^–^ = 0.59 mg L^–1^) was correlated with diminished nonverbal intellectual abilities in children between 3 and 4 years of age with the effect being more pronounced among formula-fed children as opposed to breast-fed children (see section 8.1).^469^ Increased levels of fluoride in tap water were associated with a higher risk of an attention deficit hyperactivity disorder (ADHD) diagnosis as well as increased symptoms of hyperactivity and inattention in youth between 6 and 17 years of age, especially among adolescents.^470^ No association between urinary fluoride levels and a diagnosis of ADHD or hyperactive/inattentive symptoms in youth between 6 and 17 years of age^470^ and between urinary fluoride corrected for dilution and a diagnosis of a learning disability in children aged 3–12 years was found.^471^ The absence of correlation is likely to be related also to fluctuations in elimination kinetics of fluoride (see section 7.1.3). The hypothesis that chronic fluoride intake is involved in autism spectrum disorder (ASD) etiopathology needs additional research.^380^

In 1980, fluoride was suggested as having a protective effect against Alzheimer disease.^472^ In a recent longitudinal study conducted in Scotland, even relatively low levels of aluminum (*C*_Al_ = 0.0374 ± 0.0100 mg L^–1^, range: 0.0105–0.0928 mg L^–1^) and fluoride (*C*_F_^–^ = 0.0534 ± 0.016 mg L^–1^, range: 0.0238–0.0181 mg L^–1^) in drinking water—as compared to the guideline values for aluminum and fluoride in drinking water set by the WHO^113^—were associated with effects on dementia risk.^473^

At this point, it must be noticed that correlation is not necessarily a causation. Further research on possible neurotoxic effects of fluoride on children and adults is a prerequisite before firm conclusions or recommendations can be drawn. We should not avoid discussion and criticism—papers criticized in comments^453,454^ have high scientific impact^474^ and assist in pushing research to new landmarks.

##### 7.3.2.8 Pineal Gland

The pineal gland is a mineralizing tissue with the main and most conserved function being nighttime secretion of melatonin.^475^ Fluoride has been reported to readily accumulate in the human pineal gland and could affect pineal metabolism, in a similar way as it impacts the metabolism of other calcified tissues.^476^

In female gerbils, exposure to fluoride was suggested to have a role in accelerated sexual maturation. A fluoride-free diet was observed to stimulate pineal growth in aged male rats.^477^ In humans, findings on associations between fluoride concentration in water and its effect on the pineal gland in humans are not univoqual. Results of an early study in the fluoridated area with water F^–^ concentration between 1.0 and 1.2 mg L^–1^ suggested an earlier age at menarche in relation to fluoride exposure.^478^ In another study, no association in the onset of menarcheal age in the fluoridated area (*C*_F_^–^ = 1.1 mg L^–1^) as opposed to the nonfluoridated area was reported.^479^ Recently, possible association between an increase in peripubertal fluoride and later pubertal development in boys, but not girls, exposed to fluoride through fluoridated table salt (200–250 mg kg^–1^ of F^–^) was suggested.^480^

##### 7.3.2.9 Reproductive Function

The excessive intake of fluoride might have an effect on reproduction ability. In male animals, excessive exposure to fluoride was found to negatively affect the male sperm function, including its morphology, motility, capacitation, and acrosome reaction, and damage the structure of the testis, epididymis, and prostate and function of the reproductive endocrine system.^481−486^ Sperm mitochondrial DNA copy number has been suggested as a sensitive biomarker to reflect the sperm toxicity of fluoride.^487^

The exposure to fluoride in female animals was reported to negatively affect reproductive hormone activities, maturation capacity of oocytes, and embryonic development and to cause ovarian and uterine structural damage.^488−493^

In the US, an apparent connection between the consumption of drinking water containing at least 3 mg L^–1^ of fluoride and decreased total fertility rate was reported.^494^ Excessive intake of fluoride in occupationally exposed workers or men living in the fluoride endemic area was suggested to negatively affect the semen quality and hormone levels of the hypothalamus–hypophysis–testis axis.^495−499^ Excessive intake of fluoride in women living in the fluoride endemic area was found to affect the hypothalamus–pituitary–ovary axis.^500^

### 7.4 Mitigation of Fluoride Toxicity

The first intervention in acute fluoride intoxication is an oral administration of calcium gluconate, calcium chloride, or milk, if the former is not available, to slow and reduce the absorption of fluoride.^386,501,502^ The urinary excretion rate of fluoride should be increased by intravenous administration of calcium gluconate, glucose, sodium lactate, or sodium bicarbonate. Treatment might include oxygen therapy, artificial respiration, electrocardiac conversion, and hemodialysis and should continue until normalization of vital signs and serum chemistry values.^386,502^

The early signs of chronic fluoride intoxification (e.g., polyuria and polydipsia) can be treated by limiting fluoride intake.^503,504^ The symptoms return, if treatment is discontinued. Dental fluorosis is irreversible and is treated by esthetic dentistry. Skeletal fluorosis treatment is not standardized. The success of available treatments, which include western and Chinese traditional medicine approaches, is limited.^403,505^

The emerging issues related with fluoride intake are fluoride “linked disorders”. Fluoride effects on tissues and organs other than teeth and bone should be acknowledged as the milestones in the beginning of a new era, which need to be addressed urgently.^506^

## 8 Biomarkers of Fluoride Exposure and Their Status

The term biomarker (or biological marker) is generally used in a broad sense to include almost any measurement that reflects an interaction between a biological system and a potential hazard, whether physical, chemical, or biological.^507,508^ It is useful to classify biomarkers into three types: exposure, effect, and susceptibility. Biomarkers of fluoride exposure are of value primarily for identifying and monitoring deficient or excessive intakes of biologically available fluoride, including both dietary and nondietary sources. The fluoride ion does not produce any metabolites, and thus fluoride concentrations in biological fluids or tissues can be used as indices of an individual’s exposure.^114,407^ Present exposure to fluoride might be assessed by “contemporary” biomarkers, while more chronic exposure might be assessed by “recent” or “historic” biomarkers.^508^ Biomarkers of fluoride exposure were recently reviewed; therefore, only a brief summary is presented.^509−513^

### 8.1 Contemporary Biomarkers

Fluoride concentrations in plasma, urine and urine excretion rate, saliva, milk, sweat, and bone surface have been considered to access contemporary exposure to fluoride.

Plasma fluoride concentrations are highly variable and increase with fluoride intake and age.^348,349^ The mean resting plasma concentration varied between 0.49 and 1.26 μmol L^–1^ in nonfluoridated areas with water fluoride concentrations below 0.3 mg L^–1^ and between 0.91 and 1.16 μmol L^–1^ in areas with water fluoride concentration between 0.6 and 1.2 mg L^–1^ and reached 1.84 μmol L^–1^ in areas with high natural concentrations of fluoride in water of 9.6 mg L^–1^.^510^

Daily urinary fluoride excretion is generally recommended for the estimation of daily exposure to fluoride.^511^ A linear relationship between daily urinary fluoride excretion and total daily fluorine intake for both children and adults was suggested.^354^ The excretion of fluoride in urine is reduced in individuals with impaired renal function.^101^ Urine fluoride excretion was 0.79 mg day^–1^ in humans with normal renal function, 0.53 mg day^–1^ in those with questionable renal funcation, and 0.27 mg day^–1^ in those with impaired renal function.^348^

Fluoride concentrations in submandibular/sublingual duct saliva and parotid duct saliva are preferred over whole saliva as biomarkers of fluoride exposure because whole saliva can be contaminated with fluoride from the diet, dietary fluoride supplements, and dental products. Ratios of saliva to plasma fluoride concentrations, under resting conditions, varied from 0.32 to 0.55 for parotid saliva^514^ and from 0.61 to 0.88 for submandibular saliva.^515^

Breast milk fluoride concentrations are correlated to plasma fluoride levels and range between 0.002 and 0.073 mg L^–1^ with a trend for lower concentrations in regions with low fluoride concentrations in drinking water.^407,516^ Fluoride contents of breast milk of mothers with dental fluorosis in the high altitude were surprisingly high and ranged between 0.13 and 0.99 mg L^–1^. This suggests that factors affecting the level of fluoride in breast milk need further investigation.^517−519^

Sweat fluoride concentrations were reported to be comparable to those in plasma (1–3 μmol L^–1^).^520^ The bone surface has been suggested as a terminal biomarker of acute fluoride exposure.^510,520^

Fluoride concentrations in plasma, saliva, and urine fluids and urinary excretion rate give some indication of the contemporary exposure to fluoride for groups of people but not individuals.^510^ There is a lack of data to suggest the use of breast milk and sweat as viable biomarkers of exposure to fluoride. The suitability of bone surface to humans has not been evaluated so far.

### 8.2 Recent Biomarkers

The endogenous trace element composition of hair and nails is believed to reflect the metabolic milieu of the matrix cells, including circulating blood and lymph and extracellular fluids, during their formation.^521^ The measured content is cumulative and reflects the average intake of fluoride over an extended period taking into account the growth rate.

Nail fluorine contents are influenced by age, gender, geographical area and urban/rural class.^522^ Toenails were suggested as more appropriate biomarkers of subchronic exposure to fluoride than fingernails.^522,523^ In children from 3-to-7-year-old, who are at risk for dental fluorosis, a positive correlation between fluoride concentration in drinking water (0.09 or 2.3 mg l^–1^) and nail fluorine contents (1.56 or 7.52 μg g^–1^) was observed.^522,524^ In adults, the reported mean content of fluorine in nails usually ranges between 0.49–12.5 μg g^–1^^522,525,526^ and was highly increased under occupational exposure.^527^

The reported mean F content in hair is highly variable and ranges from a few hundredths to a few tens of μg g^–1^. Hair F content in children was correlated with dental fluorosis level.^528,529^ In children and adults, hair F content was correlated with fluoride content in drinking water.^528−532^ Hair F content can be used as an indicator of occupational exposure to fluoride.^527,533,534^

Fluorine concentrations in nail and hair give some indication on the contemporary exposure to fluoride for groups of people but not individuals. Samples can be obtained noninvasively, can be easily transported and stored for long periods without degradation.^526^ The main issues related to the use of nails and hair as bioindicators present external contamination and the sample preparation method resulting in a large variation of literature results.^535^

### 8.3 Historic Biomarkers

Fluorine contents of the nonexchangeable inner compartment of bone and dentin increase with age due to continuous fluoride uptake throughout life and may serve as historic biomarkers of systemic exposure to fluoride.^114^

Factors like level of fluoride intake, age, gender, genetic background, renal function, and bone type influence bone fluorine content.^509,536^ Thus, it is not possible to establish “normal” ranges in bone for individuals, yet fluorine content can serve as an indicator of chronic exposure to fluoride. The fluorine content of bones in the nonfluorinated area increased from 200 μg g^–1^ in the first decade of life to an average of 1250 μg g^–1^ in the ninth decade.^537^ Fluorine bone contents up to a few thousand of μg g^–1^ were reported in communities with water fluoride concentrations of 1 mg L^–1^ or higher.^414,537,538^ Bone tissue is for obvious reasons collected only rarely, and the use of primary or often extracted third molars emerged as a potential biomarker of exposure to fluoride.

The fluorine content of dentin of exfoliated primary teeth in relation to fluoride in water containing from less than 0.3 to 1 mg L^–1^ of F^–^ ranged from 106 to 2699 μg g^–1^.^539^ In communities with water containing between 0.2 and 1 mg L^–1^ of F^–^, a positive correlation between dentin (101–860 μg g^–1^) fluorine contents of third molars (101–860 μg g^–1^) and dental fluorosis but not enamel F contents (39–550 μg g^–1^) and dental fluorosis was found.^540^ The same authors reported a correlation between tooth F contents and F bone contents in mice but no correlation between enamel or dentin F contents and F bone contents in humans.^541^

Reported fluorine contents in biological materials are highly variable and give some indication of exposure to fluoride, but so far normal ranges were not established. The concerns related to the reported results are the same as those identified more than a decade ago; that is, “the results are difficult to compare because: (1) sample pre-treatment methods were used that do not necessarily ensure complete release of fluoride from the sample matrix; (2) adequate information as to how the studies were conducted is not always provided; and (3) although advances in analytical techniques for trace amounts have led to re-examination of many of the published data, the majority of the data on fluorin(d)e still comes from older studies”.^357^ Further research should be encouraged starting by developing suitable CRMs for fluorine in biological materials to support the results of measurements according to GUM principles.

## 9 Conclusion

Fluorine and its compounds have unique and useful properties. For example, hydrofluorocarbons (HFCs) are nontoxic and nonreactive, and their boiling point is perfect for their use as refrigerants. A more famous example, Teflon (the polymer of tetrafluoroethylene), has low coefficient of friction and high chemical inertness. In the case of pharmaceuticals and agrochemicals, introduction of fluorine atoms often enhances chemical and metabolic stability, potency, and bioavailability. Fluorination also affects lipophilicity. As a consequence, the fluorochemical industry underwent an explosive growth during the last century, and fluorinated drugs and agrochemicals became more and more common (25–30% of newly introduced drugs and 75% of herbicides commercialized between 2010 and 2016 are fluorine-containing molecules).

Unfortunately, a side effect of the above developments was increased pollution of the environment with inorganic and organic fluorine compounds. The problem is aggravated by two factors. First, fluorine is a xenobiotic element because fluoride (its sole natural source) is scarcely available, and fluorine incorporation into organic molecules under biological conditions is a very difficult task (only a handful of organofluorine compounds can be found in nature). Second, fluorine compounds were often used before their toxicities and environmental effects were fully understood.

Studying the effects of fluorine-containing substances on the biosphere and the environment uncovered some unpleasant surprises. The only well-defined beneficial effect of fluoride is reduction of dental caries, which led to fluoridation of community water in various countries. Notably, even this positive effect is negated by too high fluoride intake. All other bioactivities of fluoride are harmful: excess fluoride intake can result in dental or skeletal fluorosis, impaired thyroid and endocrine system function, or developmental neurotoxicity. In combination with Al^3+^ ions, fluoride readily forms aluminum(III) fluorocomplexes which can activate G-protein-coupled receptors at much lower concentrations than either Al^3+^ or F^–^ alone. It is also important that fluoride accumulates in the body: a large portion of the fluoride intake is quickly deposited in bones and teeth, from which it is released quite slowly. That is why fluoride content of the bones and teeth can be used as historic biomarkers of fluoride intake. Finally, even strongly bound fluoride sources can pose a danger (for example, strongly bound fluoride in soils can harm grazing livestock).

Within inorganic fluorides, sulfur hexafluoride is notable. This compound is a chemically robust nontoxic gaseous dielectric, but it has a strong greenhouse effect.

Organofluorine compounds also have various risks. A common problem is metabolism. These compounds (especially heavily fluorinated ones) are often highly resistant to degradation and can accumulate in the environment. Such compounds are persistent organic pollutants. The presence of polyfluoroalkyl substances, fluorinated drugs, and fluorine-containing agrochemicals in surface waters and biological samples corroborates this statement. In the case of fluorinated organic compounds, which undergo metabolism, a different danger arises: metabolic processes may produce toxic fluorine-containing metabolites, e.g., fluoride or fluoroacetate. It should not be forgotten that fluorinated drugs are in the most prescribed family, and an average patient may take several such drugs at the same time, increasing the risk. Many fluorinated drugs are known, where toxic fluoro-metabolites considerably contribute to side effects (for example, α-fluoro-β-alanine and fluoroacetate metabolites of the anticancer drug 5-fluorouracil), and some of them had to be withdrawn or restricted (for example, anesthetic agent methoxyflurane). Naturally, not all problems are metabolic in origin. Chlorofluorocarbons (CFCs), which were mainly used as refrigerants, were banned because they seriously damaged the ozone layer, producing chlorine radicals via their degradation in the stratosphere. Hydrofluorocarbons (HFCs), their replacements, have strong greenhouse effects.

Concerning the above problems, various actions should be taken. First of all, in light of more recent studies about the adverse effects of F^–^, fluoride intake should be decreased. To achieve this goal, it would be worth limiting the fluoride content of drinking water (which has a high influence on fluoride intake) more strictly. This would be the easiest way to reduce fluoride intake in countries where community water fluoridation is practiced. Fluoride pollution should be reduced too. Important sources of fluoride pollution are the aluminum industry [electrolysis of Al_2_O_3_ is performed in cryolite (Na_3_AlF_6_)] and fluoride impurity of phosphate fertilizers (superphosphate can contain 1–3% fluoride). Currently, only the first source is regulated. Further thorough studies on the physiological effects of fluoride and aluminum(III) fluorocomplexes are also welcomed. Finally, reliable and standardized methods are required to assess fluoride intake more accurately from fluoride biomarkers.

Within organofluorine compounds, CFCs possessing ozone-depleting effects are already banned, and some perfluoroalkylated surfactants (perfluorooctanesulfonic acid and perfluorooctanoic acid) were phased out because of their harmful effects (for example, carcinogenicity). Various fluorinated drugs were also withdrawn because of their side effects. However, further studies are needed on the metabolism of various fluorine-containing groups and substances to identify derivatives, which are dangerous to human health or to the environment. Such studies would be especially important in the case of fluorine-containing drugs and agrochemicals. In addition, to reduce environmental pollution, organofluorine compounds should only be used when it is truly necessary. For example, many effective fire-fighting foams are now free from polyfluoroalkyl substances.

From a green chemistry viewpoint, recycling and recovery of fluorine compounds would be beneficial too. Currently, such processes are not widespread, but they do have potential. For example, the fluoride content of phosphate fertilizers and the phosphogypsum byproduct can be easily recovered as hexafluorosilicic acid (H_2_SiF_6_). This would not only reduce fluoride pollution but also provide a valuable intermediate, which can be used to produce various fluorine compounds (e.g., cryolite). In theory, taking into account the volume of phosphate fertilizer production, a sufficient quantity of H_2_SiF_6_ can be produced to considerably reduce the need for fluorspar (CaF_2_) mining.

## Abbreviations

5′-FDA phosphorylase: 5′-fluoro-5′-deoxyadenosine phosphorylase
5-FDRP isomerase: 5-fluoro-5-deoxy-d-ribose-1-phosphate isomerase
5-FDRulP aldolase: (3*R*,4*S*)-5-fluoro-5-deoxy-d-ribulose-1-phosphate aldolase
ACP: acyl carrier protein
ADHD: attention-deficit/hyperactivity disorder
AI: adequate intake
AS: atherosclerosis
ASD: autism spectrum disorder
ATP: adenosine triphosphate
BW: body weight
CDC: Centers for Disease Control and prevention
CKD: chronic kidney disease
CLD: certainly lethal dose
CHF: congestive heart failure
CNS: central nervous system
CoA: coenzyme-A
CRM: certified reference material
DW: dry weight
EFSA: European Food Safety Authority
EU: European Union
F-ISE: fluoride-ion selective electrode
F: fluorine as element or fluorine in any of its forms
F^–^: fluoride anion
fluorinase: 5′-fluoro-5′-deoxyadenosine synthase
GDP: guanosine diphosphate
GI: gastrointestinal
GPCR: G protein-coupled receptor
GUM: guide to the expression of uncertainty in measurement
HTN: hypertension
IOM: Institute of Medicine
MUF: maternal urinary fluoride
NAD^+^: nicotinamide adenine dinucleotide
NADH: reduced form of nicotinamide adenine dinucleotide
NADP^+^: nicotinamide adenine dinucleotide phosphate
NADPH: reduced form of nicotinamide adenine dinucleotide phosphate
PLP: pyridoxal phosphate
PTD: probable toxic dose
PTH: parathyroid hormone
UDP-glucose: uridine diphosphate glucose
UK: United Kingdom
US: United States
USDA: United States Department of Agriculture
TSH: thyroid-stimulating hormone
WHO: World Health Organization
